# Isocyanate-Free Polyurea Synthesis via Ru-Catalyzed Carbene Insertion into the N–H Bonds of Urea

**DOI:** 10.1021/acs.macromol.2c01457

**Published:** 2022-10-17

**Authors:** Felix J. de Zwart, Petrus C. M. Laan, Nicole S. van Leeuwen, Eduard O. Bobylev, Erika R. Amstalden van Hove, Simon Mathew, Ning Yan, Jitte Flapper, Keimpe J. van den Berg, Joost N. H. Reek, Bas de Bruin

**Affiliations:** † Homogeneous, Supramolecular and Bio-Inspired Catalysis Group, van ’t Hoff Institute for Molecular Sciences (HIMS), University of Amsterdam, Science Park 904, 1098 XH Amsterdam, The Netherlands; ‡ Amsterdam Institute for Life and Environment, Environmental and Health, Free University of Amsterdam, 1081 HV Amsterdam, The Netherlands; § Akzo Nobel Decorative Coatings B.V., Rijksstraatweg 31, 2171 AJ Sassenheim, The Netherlands; ∥ Akzo Nobel Car Refinishes B.V., Rijksstraatweg 31, 2171 AJ Sassenheim, The Netherlands

## Abstract

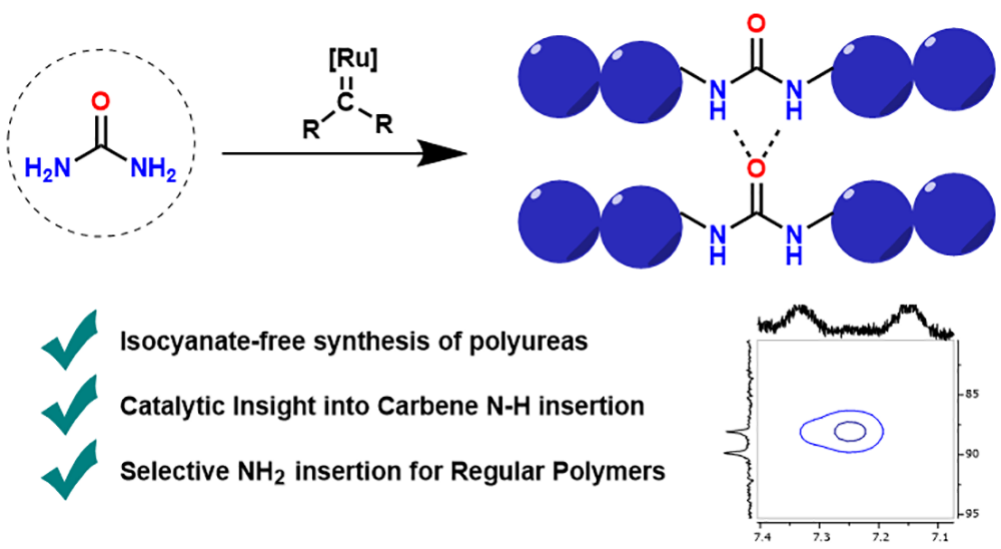

Polyureas have widespread applications due to their unique material properties. Because of the toxicity of isocyanates, sustainable isocyanate-free routes to prepare polyureas are a field of active research. Current routes to isocyanate-free polyureas focus on constructing the urea moiety in the final polymerizing step. In this study we present a new isocyanate-free method to produce polyureas by Ru-catalyzed carbene insertion into the N–H bonds of urea itself in combination with a series of bis-diazo compounds as carbene precursors. The mechanism was investigated by kinetics and DFT studies, revealing the rate-determining step to be nucleophilic attack on a Ru–carbene moiety by urea. This study paves the way to use transition-metal-catalyzed reactions in alternative routes to polyureas.

## 1 Introduction

**Scheme 1:**
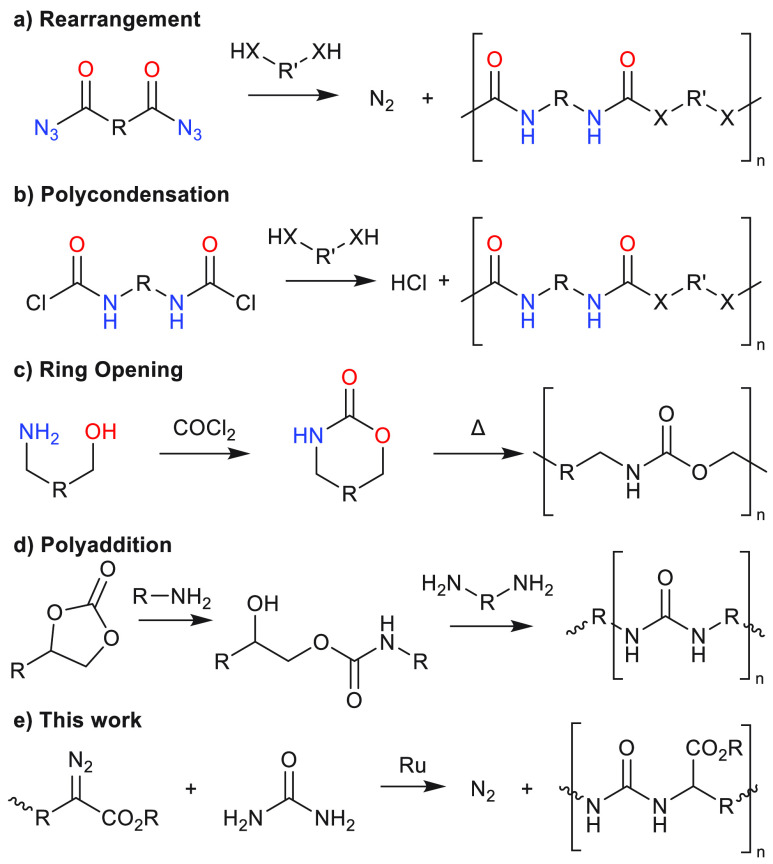
Isocyanate-Free Synthetic Approaches to Polyurethanes (X = O) and Polyureas (X = NH)

Polyurea-based elastomers find widespread applications in foams and coatings due to their unique material properties, such as tensile strength and tear resistance.^1^ Routes toward isocyanate-free polyureas and polyurethanes which make use of more benign monomers are of interest due to the toxicity of isocyanates and difficulties in handling these reactive and humidity-sensitive reagents.^2,3^ The currently available isocyanate-free routes toward polyureas and polyurethanes can be divided into four broad categories, shown in Scheme 1.^4−6^ In rearrangement pathways (Scheme 1a) isocyanates are formed in situ through Curtius (depicted), Hoffman, or Lossen rearrangements.^7^ In polycondensation, amines are prefunctionalized with either phosgene or another carbonyl source to furnish a blocked isocyanate, which can then react with an amine or alcohol to yield the desired polyurea (Scheme 1b). Ring-opening pathways use cyclic carbamates or ureas that are then polymerized through transcarbamoylation under high temperatures (Scheme 1c). Polyaddition pathways use cyclic carbonates as precursors for polyurethanes, which can be aminolyzed to polyureas using excess amine (Scheme 1d).^8−11^ Most of these isocyanate-free routes have their drawbacks; for example, in the formation of isocyanates in situ (Scheme 1a), acyl azides are used as precursors which are also harmful substances. The polycondensation route (Scheme 1b) releases side products during curing which limits industrial usage, which in the case of ring-opening is limited by the high temperatures required (Scheme 1c). The most promising route currently utilizes amines and carbonates (Scheme 1d) as precursors with low toxicity.^12,13^

Interestingly, the use of (transition-metal) catalysis to arrive at polyurea and polyurethane structures has not been developed as much. Transition-metal-catalyzed polymerizations using diazo compounds provide a powerful alternative synthetic tool to otherwise difficult to access structures. For example, a variety of diazo compounds can be used as monomers in single-component polymerization, leading to highly functionalized polymers.^14,15^ Polyesters and polyethers can be synthesized from their respective alcohols and acids in a copolymerization reaction with diazo compounds through O–H insertion reactions.^16−18^ Using amines as precursor, a recent article by Ihara and co-workers has reported on the synthesis of polyamines through ruthenium-catalyzed N–H insertion, a continuation of the research on the synthetic utility of C–N bond formation using diazo compounds.^19−24^ Ureas have been used as nucleophiles in transition-metal catalysis such as Pd-catalyzed carboaminations and more recently as nitrene precursors in Ru-catalyzed C–H amination.^25−28^ On the basis of this rationale, we wondered if it would be possible to use N–H insertions to synthesize polyureas as shown below (Scheme 1e). Whereas in all the known approaches (Scheme 1a–d) the urea moiety is constructed in the final step, this approach differs fundamentally with urea itself operating as a nucleophile to react with a ruthenium-centered carbene. Furthermore, with the tunability of diazo precursors a variety of pendant side groups can be attached to influence the material properties of polymers. In this proof-of-concept study we present a new strategy to prepare polyureas by utilizing Ru-catalyzed N–H insertions on urea.

## 2 Results and Discussion

### 2.1 Bisfunctional Diazo Compound Synthesis

**Scheme 2:**
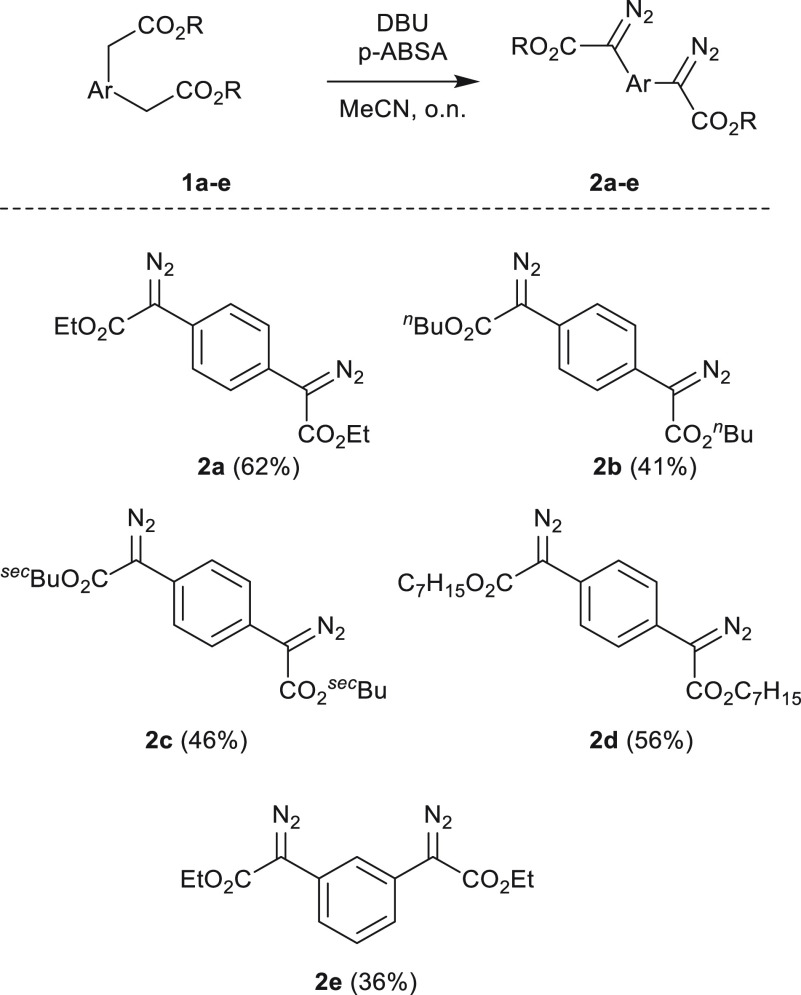
Synthesis of Bis-Diazo Compounds **2a**–**e** (Yield after Column Chromatography in Parentheses)

The bisfunctional diazo compound diethyl 1,4-phenylenebis(diazoacetate), **2a**, has been described in the literature recently, and we decided to expand on the scope of phenylenebis(diazoacetate) compounds by changing the ester substituents.^29^ These donor–acceptor type diazo compounds were specifically chosen because carbene dimerization is known to be greatly suppressed when compared to other diazo compounds.^30^ With phenylenediacetic acid as a starting substrate, five different bisfunctional diazo compounds were synthesized in a two-step procedure. The esters are readily synthesized under Dean–Stark conditions in toluene with catalytic amounts of sulfuric acid. The active methylene carbon can be functionalized with a diazo moiety through a Regitz diazo transfer using *p*-acetamidobenzenesulfonyl azide (*p*-ABSA) as a diazo transfer reagent; products **2a**–**e** were obtained in fair to moderate yields (62–36%) as pure compounds after column chromatography (Scheme 2). The diazo compounds were characterized by ^1^H and ^13^C NMR and HR–MS (see the Supporting Information). Compared to the more often used tosyl azide, *p*-ABSA has the advantage of being less explosive and impact sensitive and is therefore a safer preferred alternative.^31^

### 2.2 Polymerization

After a successful test reaction using urea and ethyl diazo(phenyl)acetate which provided the desired product (molecular structure determined by X-ray diffraction shown in Figure S1, we focused on optimization of the polymerization reaction. The initial exploration of polymerization conditions was started by polycondensation of **2b** with urea in the presence of dichloro(*p*-cymene)ruthenium(II) dimer. This provided a polymeric material, which could be separated from the reaction mixture by precipitation from diethyl ether. The polymer was analyzed by SEC, which revealed a weight-averaged molecular weight (*M*_w_) of 7.1 kDa. Increasing the ruthenium catalyst loading led to a higher yield (entry 2, Table 1), while increasing the diazo stoichiometry resulted in a lower yield (entries 3–5, Table 1). These two observations point to a step-growth polymerization process. Decreasing the reaction temperature or changing the concentration also led to a lower yield or molecular weight (entries 6–8, Table 1). With the optimized polymerization conditions at hand, the scope of the reaction was explored by changing the diazo substituent (Table 2), where diazos **2a**–**e** were used to synthesize polymers **3a**–**e**. As a solvent for precipitation, hexane was used instead of diethyl ether, as diethyl ether was polar enough to dissolve polymers **3c**, **3d**, and **3e**. This provided polymeric materials in good to moderate (65–45%) yields. The slight differences in obtained yields are presumably a result of the precipitation procedure, as the more soluble *sec*-butyl-substituted polymer **3c** provided the lowest yield. The acquired polymers are soluble in solvents such as DCM, chloroform, and dimethyl sulfoxide and poorly soluble in hexane and methanol and insoluble in water. Through size exclusion chromatography (SEC) analysis based on linear polystyrene standards, weight-average molar masses (*M*_w_) and dispersities (*Đ*) of the produced polyureas were estimated to be in the ranges of 3.9–7.1 kDa and 1.3–1.6, respectively (Table 2). We then continued to use polymer **3a** as a starting point for polymer characterization.

**Table 1:**
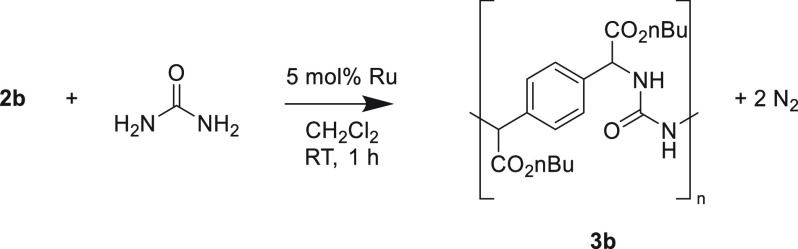
Polycondensation of **2b** with Urea^a^

No.	Deviations	Yield^b^ (%)	*M*_w_ (kDa)^c^	*Đ*^c^
1	none	65	7.1	1.50
2	10 mol % Ru	94	6.3	1.61
3	1.1 equiv diazo	55	5.3	1.53
4	1.2 equiv diazo	25	6.1	1.57
5	1.3 equiv diazo	0		
6	0 °C	22	2.8	1.38
7	25 mL DCM	30	7.4	1.58
8	1 mL DCM	77	3.6	1.24

a Conditions: urea (100 μmol), **2b** (100 μmol), dichloro(*p*-cymene)ruthenium(II) dimer (2.5 μmol), DCM (2.5 mL), room temperature, 1 h.

b Determined by gravimetry after precipitation.

c *M*_w_/*M*_n_ as determined by SEC in DCM.

**Table 2:** Polycondensation of Urea with Different Bisfunctional Compounds^a^

Polymer	Yield (%)^b^	*M*_w_ (kDa)^c^	DOP (SEC)^c^	*Đ*^c^	*T*_g_ (°C)^d^
**3a**	56	4.9	16.2	1.5	141
**3b**	65	7.1	18.6	1.6	97
**3c**	45	4.0	13.1	1.4	131
**3d**	57	4.3	9.6	1.3	82
**3e**	54	3.9	11.0	1.5	89

a Conditions: urea (100 μmol), diazo **2a**–**e** (100 μmol), dichloro(*p*-cymene)ruthenium(II) dimer (2.5 μmol), DCM (2.5 mL), room temperature, 1 h.

b Determined by gravimetry after precipitation.

c Determined by SEC in DCM.

d Determined by DSC.

### 2.3 Characterization

**Figure 1:**
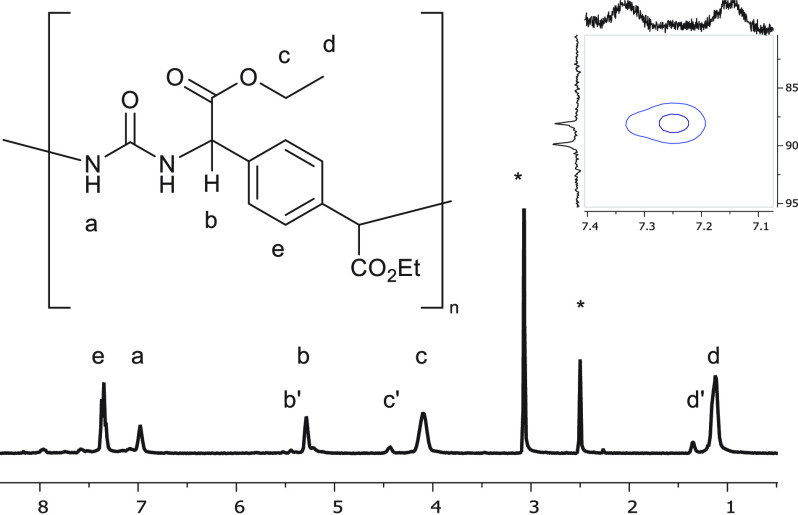
^1^H NMR spectra of 2a (∗: solvent). Inset: ^1^H–^15^N HSQC using ^15^N-labeled urea.

**Figure 2:**
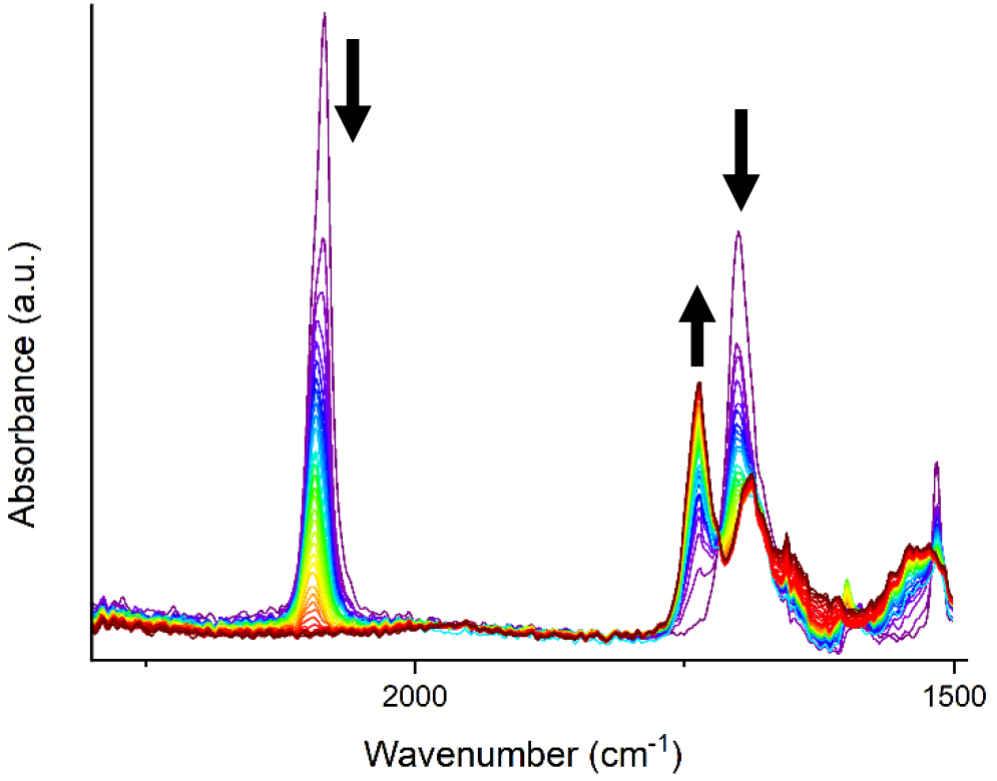
Operando FT–IR monitoring of ruthenium-catalyzed polymerization of **2a** and urea. One trace corresponds to 15 s.

The FT–IR spectrum (Figure 2) of polyurea **3a** reveals bands located at 3367, 2981, 1732, and 1643 cm^–1^, which are related to urea N–H, aromatic C–H, ester C=O, and urea C=O stretching vibrations, respectively. These bands support the incorporation of both functional groups in the polymer chain, and the disappearance of the diazo stretch vibration of the starting material shows full conversion of the bis-diazo compound. The ^1^H NMR spectrum in DMSO-*d*_6_ at 80 °C (Figure 1) shows characteristic signals for the ethyl chain introduced with comonomer **2a** at 1.11 and 4.10 ppm (H_d_ and H_c_) and the aromatic protons at 7.35 ppm (H_e_). Two new signals at 6.98 and 5.29 ppm are allocated to the urea H_a_ and benzylic H_b_ protons. This assignment was confirmed by ^15^N-labeling of the urea prior to catalysis, providing a ^15^N-labeled polymer **3a**. The acquired ^1^H–^15^N HSQC displays a negative cross-peak, indicative of a secondary amine, between H_a_ and a doublet in ^15^N NMR at 89 ppm with a coupling of 91 Hz, supporting the assignment of H_a_ as the urea N–H proton (inset of Figure 1; see also Figures S51 and S52). To confirm the anticipated polymer structure containing alternating urea and phenylene–diester repeating units, ^13^C NMR analysis was performed, revealing both the ester and urea carbons at 170 and 156 ppm, respectively (Figure S50). The ^13^C NMR signals belonging to the aromatic ring were located at 137 and 127 ppm. The carbons of the ethyl chain resonate at 60 and 13 ppm, and the benzylic carbon reveals a signal at 56 ppm. The NMR interpretation of polymers **3b**–**3e** is similar to that of **3a** and in accordance with the proposed structure. When using chiral diazo **3c**, the benzylic carbon is observed as two signals of equal intensity at 57.3 ppm. This indicates that chiral secondary structures of the resulting polyureas could be accessible when using chiral catalysts capable of enantioselective N–H insertion.

### 2.4 End-Group Analysis by Mass Spectrometry

To further understand the chemical structure of the polymers, the end groups were studied by mass spectrometry. The relatively low molecular weights obtained for these polyureas could possibility arise from cyclization of the polymers. Indeed, when polymer **3b** was subjected to ESI–MS analysis, we observed only signals corresponding to oligomers without end groups (see Figures S24–S30). The absence of end groups strongly indicates the presence of cyclic structures in the polymer.^32^ Polymers **3a**, **3b**, and **3e** were further studied by MALDI–ToF–MS, which also provided signals in accordance with polymers without an end group (Figures S31–S33). While the data point to formation of mainly cyclic polymers, we cannot fully exclude that some of the higher molecular weight chains (*n* > 8) could be partially linear though (in MALDI–ToF–MS for *n* > 8 solvent/water and multiple Na^+^ adduct formation leads to broad, overlapping peaks which complicates chain-end assignment). Formation of mainly cyclic polymers explains the relatively low molecular weights obtained for the polymers in this study, as cyclization is an effective termination pathway (see Scheme S1).

### 2.5 Material Properties

To investigate the effect of the ester substituent on the material properties, the glass transition temperature (*T*_g_) was measured. Incorporation of soft segments within polyurea segments is known to lead to a decrease in glass transition temperature.^33,34^ Indeed, the heptyl-substituted polymer **3d** has the lowest *T*_g_ and ethyl-substituted diazo **3a** has the highest *T*_g_ (Table 2). Furthermore, 1,3-phenylene-substituted polymer **3e** is observed to have an intermediate *T*_g_ of 89 °C while having the same molecular formula as **3a**, showing that main-chain connectivity can be used as an effective tool to control the material properties.

### 2.6 Kinetics

**Scheme 3:**
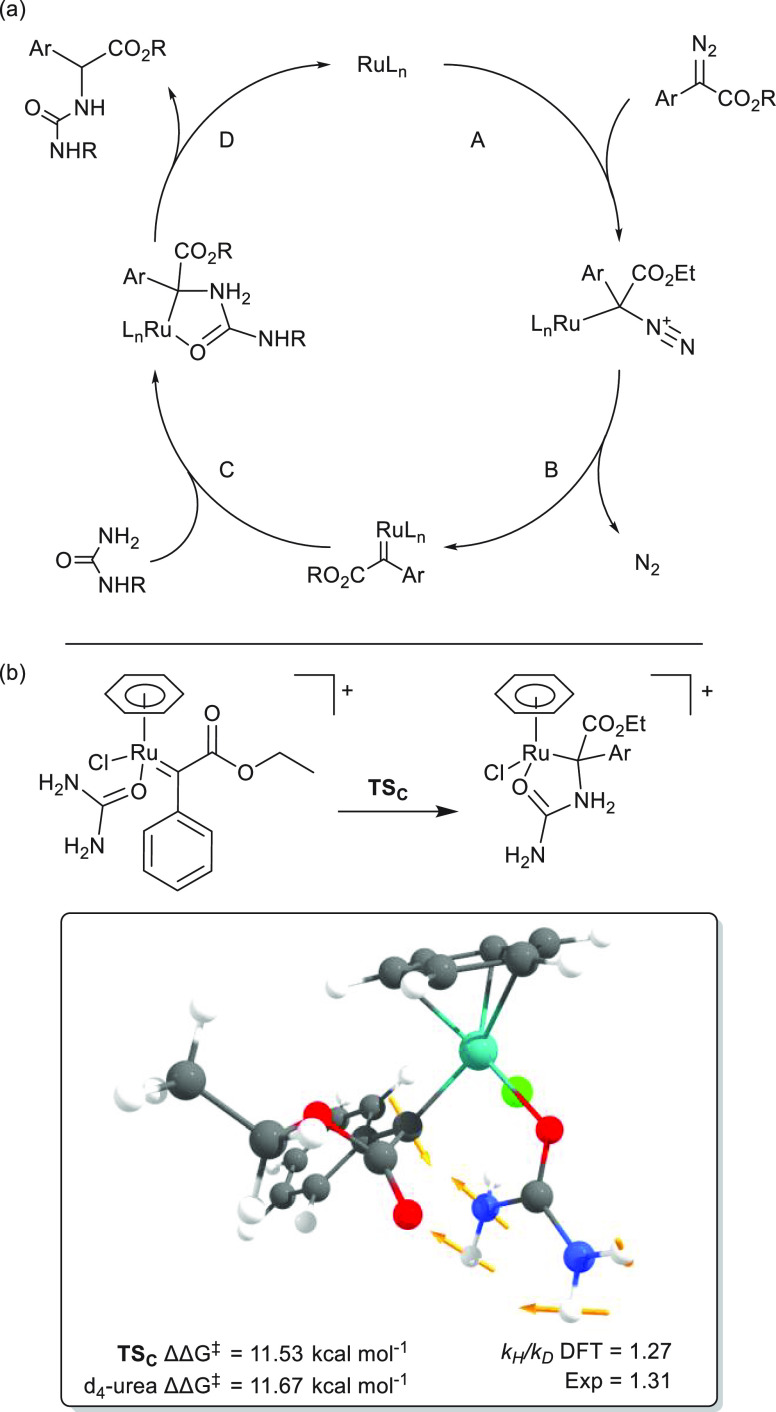
(a) Catalytic Cycle for Ruthenium Catalyzed Carbene N–H Insertion: (A) Diazo Coordination, (B) Carbene Formation, (C) Nucleophilic Attack, and (D) Proton Shift; (b) Transition State Structure for Nucleophilic Attack by Urea (CPK Coloring)

To obtain more insight into the mechanism of polymerization, the polycondensation of **2a** with urea was followed by operando IR spectroscopy, as shown in Figure 2. A clear decrease in the signal at 2091 cm^–1^_,_ corresponding to the disappearance of the diazo moiety, of the monomer was observed. Furthermore, a new signal corresponding to the C=O stretch vibration of the polymer is observed at 1734 cm^–1^. Using these two signals the conversion and yield over time was monitored to provide insight into the kinetics of this reaction. When using 1,3-substituted diazo **2e**, the conversion decreased linearly over time, indicating zeroth order in diazo monomer concentration (Figure S7). On the other hand, with 1,4-substituted diazo **2a** some rate dependence on conversion was observed. To investigate this further with improved time resolution, kinetic experiments were undertaken to derive the rate law for both diazo **2a** and **2e** by monitoring the gas production of the reaction with a bubble counter.^35^ For diazo **2a**, the diazo order dependence in the range of 5–10% catalyst loading was found to not clearly fit to a reaction order (Figures S16–S21). We believe this to be an effect of the *para* substitution on the diazo, causing the electronics of one diazo moiety to be influenced by reactions at the other, thereby complicating the reaction profile. Therefore, the rest of the kinetics were undertaken using *meta*-substituted diazo **2e**, for which the catalytic profile is more clear. As such, for diazo **2e** a first-order rate dependence in catalyst concentration was found in the range of 5–10% catalyst loading, excluding any multinuclear pathways (Figures S8–S10). For diazo **2e** a zeroth-order dependence of rate upon diazo concentration was found (Figures S11–S13). The low solubility (0.2 mg/mL, see the Supporting Information for details) of urea in dichloromethane prevents us from determining the order in urea. The zeroth-order kinetic in diazo **2e** suggests that diazo activation is fast, and presumably the follow-up reaction with urea is the rate-limiting step. This contrasts with previous work on rhodium X–H insertions, for which carbene formation was shown to be rate-limiting.^36^ The kinetics in this case should (at least in part) be influenced by the low solubility of urea, which causes the concentration of urea to remain low and constant throughout. Transition-metal-catalyzed X–H insertions are generally understood to proceed via a four-step mechanism (Scheme 3a, *vide infra*), consisting of (A) diazo coordination, (B) carbene formation, (C) nucleophilic attack, and (D) proton shift.^37,38^ Having excluded A and B as rate determining, kinetic isotope effect studies were undertaken using *d*_4_-urea and diazo **2e**. Using the initial rates until 20% conversion the kinetic isotope effect (KIE) for urea was found to be *k*_H_/*k*_D_ = 1.31 (Figures S14 and S15), corresponding to a secondary kinetic isotope effect.^39^ This KIE strongly indicates the rate-determining step to be nucleophilic attack, as step C involves rehybridization of the urea nitrogen for which a larger KIE would be expected. This result is different from known copper and rhodium systems,^37^ for which fast (non-rate-limiting) nucleophilic attack of amines and alcohols to the carbene was observed. The low solubility and poor nucleophilicity of urea explain why nucleophilic attack is rat-determining in this case.

### 2.7 Density Functional Theory

To further investigate the catalytic mechanism, we pursued DFT calculations to shed light on the nature of the kinetic isotope effect (gas phase, BP86, def2-TZVP, D3). As previous experiments indicated no involvement of the diazo compound in the rate-determining step, we first investigated steps A and B to confirm this. As chlorido ligands are known to be displaced from similar ruthenium arene species by carboxamide donors, an intramolecular mechanism in which urea first coordinates to ruthenium (via its carbonyl group) was investigated (Scheme 3b).^40^ Coordination of phenyl-2-diazoacetate to the starting complex (step A, Scheme 3a) was found to be downhill by −2.3 kcal mol^–1^. Then, release of dinitrogen from the diazo adduct to form the ruthenium carbene was found to have a small barrier of 6 kcal mol^–1^ (step B, Scheme 3a). This step is strongly exergonic, and release of dinitrogen was found to be downhill by −39.0 kcal mol^–1^. The barrier Δ*G*^‡^ for nucleophilic attack from urea on the carbene (step C, Scheme 3a) was found to be 11.53 kcal mol^–1^, in accordance with the experimentally observed fast reaction at room temperature.

Interestingly, a slight dependence of the transition state energy on the orientation of the carbene was found if the aryl moiety is pointing toward the urea the barrier was slightly increased to 12.3 kcal mol^–1^. By visualizing the displacement vectors, a N–H bending vibration can be observed in the intrinsic reaction coordinate of the transition state (Scheme 3b), pointing toward a secondary kinetic isotope effect for deuteration of this position. Furthermore, by recalculating the vibrational correction to the Gibbs free energy of the starting material and the transition state, a DFT estimate of the kinetic isotope effect can be calculated. The transition state barrier for *d*_4_-urea ΔΔ*G*^‡^ was found to be 11.67 kcal mol^–1^ corresponding to *k*_H_/*k*_D_ = 1.27, in accordance with the experimentally derived value. The DFT results therefore support nucleophilic attack to be rate-determining for the ruthenium-catalyzed copolymerization of urea and bis-diazos. The subsequent proton transfer through a 1,5-proton shift toward the ester moiety was calculated to have a DFT barrier of 13.5 kcal mol^–1^; however, as this is presumably substrate or solvent assisted, the experimental barrier in solution is expected to be lower.

### 2.8 Copolymerization

Commercial polyureas get their unique properties from having a mixture of soft and hard segments within their polymer backbone.^41^ This is generally achieved by mixing different polyamines with isocyanates, and as such we were interested in whether the developed Ru-catalyzed polycondensation was suitable for copolymerization. To incorporate a soft segment, a poly(tetramethylene oxide) (PTMO) oligomer (*M*_n_ = 1000 g/mol) was end-capped with phenyldiazoacetate units in a two-step procedure.

First, phenylacetyl chloride was used to introduce phenylacetyl groups after a similar Regitz diazo transfer procedure was used to introduce diazo groups on the end, providing telechelic polymer **2f** (*M*_n_ = 1500 g/mol, determined by SEC). When reacted with urea under the optimized polycondensation conditions as described above (Table 3, entry **3f**), a polymer with a molecular weight of 8.3 kDa was obtained indicating successful copolymerization, with a *T*_g_ observed at 34 °C (Figure S23). To incorporate more hard blocks within the polymer, copolymerization with diazo **2a** was performed. When telechelic polymer **2f** was copolymerized with **2a**, a material with molecular weight 12.2 kDa and a glass transition temperature of 39 °C was obtained (Table 3, entry **3g**). NMR integration of **3g** indicates that the ratio of incorporation is consistent with the feed ratio (Figure S67). Furthermore, the molecular weight distributions obtained from SEC in all cases displayed monomodal distributions (Figure S23), showing the efficacy to use multiple diazo compounds in copolymerization. Further studies are directed at further tuning the material properties to not only emulate the molecular structure of polyureas but also gain the desirable features present in commercial polyureas.

**Table 3:**
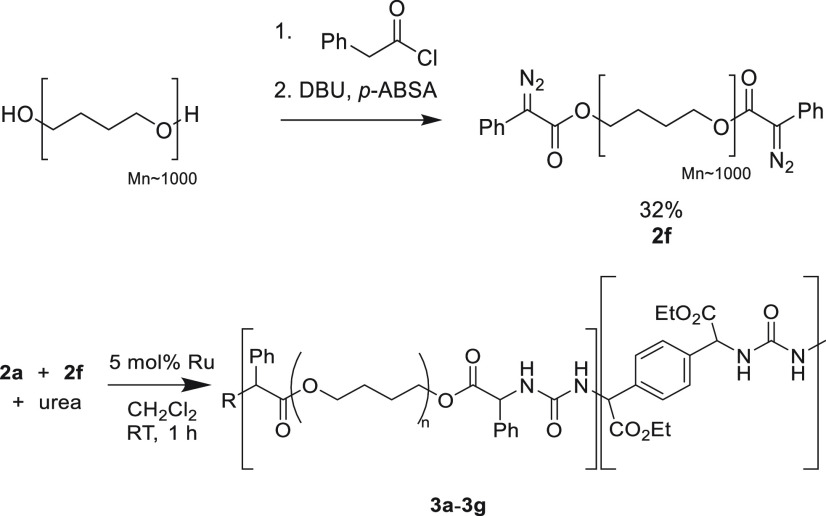
Copolymerization of an End-Group Functionalized Poly(tetramethylene oxide) (PTMO), Diazo **2a**, and Urea^a^

Polymer	**2a** (mmol)	**2f** (mmol)	Urea (mmol)	*M*_w_ (kDa)^b^	*Đ*^b^	*T*_g_^c^
**3a**	100	0	100	4.9	1.54	141
**3f**	0	100	100	8.3	2.1	34
**3g**	100	100	200	12.2	2.09	39

a Conditions: urea (100 μmol), **2b** (100 μmol), dichloro(*p*-cymene)ruthenium(II) dimer (2.5 μmol), DCM (2.5 mL), room temperature, 1 h.

b Determined by SEC in DCM.

c Determined by DSC.

## 3 Conclusion

In this work, we have demonstrated the capability to synthesize polyureas by ruthenium-catalyzed N–H insertion reactions, wherein urea uniquely functions as a nucleophile. With this protocol, polyurea moieties are accessible through a route completely free of isocyanate. The formed polymers were found to have material properties tunable through side-chain or main-chain substitution. The mechanistic investigations show nucleophilic attack of urea on the formed carbene to be rate-determining. This work shows the possibility of using diazo compounds in combination with transition-metal catalysis to furnish novel routes toward isocyanate-free polyureas.

## Supporting Information Available

Synthetic procedures; NMR characterization of compounds and polymers; kinetics data; DSC profiles; MS analysis of polymers; DFT details and coordinates of all stationary points (PDF)
Crystallographic details for **5** (CIF)

## Supplementary Material

ma2c01457_si_002.pdf

ma2c01457_si_001.cif
