# Supplementary material for: Isocyanate-Free Polyurea Synthesis via Ru-Catalyzed Carbene Insertion into the N–H Bonds of Urea
Source: Macromolecules. 2022 Oct 17;55(21):9690–6. doi: 10.1021/acs.macromol.2c01457 (PMC9648342; doi:10.1021/acs.macromol.2c01457)
Supplement: Supplementary file 1 — ma2c01457_si_002.pdf [file ma2c01457_si_002.pdf]

Supplementary Material for:

Isocyanate-free Polyurea Synthesis via Ru-Catalyzed Carbene Insertion into the N–H Bonds of Urea

Felix J. de Zwart,[1] Petrus C. M. Laan,[1] Nicole S. van Leeuwen,[1] Eduard O. Bobylev,[1] Erika R. Amstalden van Hove,[2] Simon Mathew,[1] Ning Yan,[1] Jitte Flapper,[3] Keimpe J. van den Berg,[4] Joost N. H. Reek[1] and Bas de Bruin[1*]

[1]Homogeneous, Supramolecular and Bio-Inspired Catalysis Group, van 't Hoff Institute for Molecular Sciences (HIMS), University of Amsterdam, Science Park 904, 1098 XH Amsterdam, The Netherlands. [2]Amsterdam Institute for Life and Environment, Environmental and Health, Free University of Amsterdam, The Netherlands. [3]Akzo Nobel Decorative Coatings B.V., Rijksstraatweg 31, 2171 AJ Sassenheim, The Netherlands. [4]Akzo Nobel Car Refinishes B.V., Rijksstraatweg 31, 2171 AJ Sassenheim, The Netherlands.

Contents

General Considerations....3
General procedures. 4
  Esterification. 4
  Diazo transfer reactions.[3] 5
  Synthesis of end-group functionalized polytetramethylene oxide 2f. 7
  Ruthenium catalyzed polycondensation of ethyl diazo(phenyl)acetate with urea. 8
  Single Crystal XRD 9
  Ruthenium catalyzed polycondensation of bis-diazos 2a-e with urea. 11
Gravimetric determination of the solubility of urea in DCM....12
Kinetics by in-situ ReactIR. 13
Kinetics by bubble counting....16
  Method 16
DSC Data....27
SEC traces and DSC data of copolymers 28
End-group analysis by Mass Spectrometry....29
ESI-MS 31
MALDI MS 35
NMR Spectra 38
DFT 57
  Coordinates of all structures....60
References 72

General Considerations.

Materials. Toluene (VWR Chemicals), 1,4-phenylenediacetic acid (Tokyo Chemical Industries; >98.0%), n-butanol (Sigma-Aldrich), *tert*-butyl alcohol (Sigma-Aldrich), cyclohexanol (VWR Chemicals), *n*-icosanol (Sigma-Aldrich), heptane (VWR Chemicals), sulphuric acid (Sigma-Aldrich), sodium bicarbonate (Sigma-Aldrich), sodium hydroxide (Sigma-Aldrich), sodium sulphate (Sigma-Aldrich), *p*-acetamidobenzenesulfonyl azide (Sigma-Aldrich; 97%), 1,8-diazabicyclo[5.4.0]undec-7-ene (DBU; Sigma-Aldrich; 98%), ammonium chloride (VWR Chemicals), dichloromethane (VWR Chemicals), acetonitrile (VWR Chemicals), n-hexane (VWR Chemicals), n-pentane (VWR Chemicals), ethanol (VWR Chemicals), diethyl-(1,4-phenylene)diacetate (Tokyo Chemical Industries; >98.0%), silica-60 (VWR Chemicals), ethyl acetate (VWR Chemicals), dichlorido(*p*-cymene)ruthenium(II) dimer ([$RuCl_2$(*p*-cymene)]$_2$; SEQENS; 98%), diethyl ether (VWR Chemicals), chloroform (Sigma-Aldrich), urea (Alfa Aesar; 99.0 – 100.5%, crystalline), diethyl 2,2'-(1,4-phenylene)diacetate (**1a**, Tokyo Chemical Industries; >98.0%) were used as received. Diethyl 2,2'-(1,3)phenylenebisacetate (**1e**) was prepared according to a previously published procedure.[1] Diethyl 2,2'-(1,4)phenylenebis(2-diazoacetate) (**2a**) was prepared according to a previously published procedure.[2] Ethyl (phenyl)diazoacetate (**4**) was prepared according to a previously published procedure.[3] ^{1}H (500, 400 MHz) and ^{13}C (125, 101 or 75 MHz) spectra were recorded on a Bruker DRX 500 MHz or a Bruker AVANCE 400 MHz spectrometer or on a Bruker DRX 300 MHz spectrometer. NMR spectra of polymers **3a-h** were measured at 80 °C. ^{1}H and ^{13}C spectra were referenced against residual solvent signal, while 15N spectra were externally calibrated against urea in DMSO-d_6. FD-MS spectra were collected on an AccuTOF GC v 4g, JMS-T100GCV Mass spectrometer (JEOL, Japan) equipped with a Carbotec emitter or a LiFDi probe (FD) equipped with an FD Emitter, Linden CMS GmbH. A typical current rate of 51.2 mA/min over 1.2 min and a flashing current 40 mA on every spectrum of 30 ms was used. Size exclusion chromatography (SEC) was used to determine the polymer number-average molecular weight (M_w) and degree of dispersion ($M_\mathrm{w}/M_\mathrm{n}$). SEC measurements were performed on a Shimadzu LC-20AD system with two PLgel 5 µm MIXED-C columns (Polymer Laboratories) in series and a Shimadzu RID-10A refractive index detector. DCM was used as mobile phase at a flow rate of 1 mL/min and T = 35 °C. Polystyrene standards in the range of 760 – 1 880 000 g mol^{-1} (Aldrich) were used for calibration. The glass transition temperature ($\mathrm{T_g}$) of the polymers was determined by differential scanning calorimetry (DSC). Measurements were performed on a Perkin Elmer Jade DSC. Samples were heated from 20°C to 180°C at a heating rate of 10°C/min followed by an isothermal step for 5 min. A cooling cycle to 20°C at a rate of 10°C/min was performed prior to a second heating run to 180°C at the same heating rate. The $\mathrm{T_g}$ was defined as the temperature of the midpoint of a heat capacity change on the second heating run. The Universal Analysis 2000 software was used for data acquisition.

General procedures.

Esterification. Toluene (40 mL), 1,4-phenylenediacetic acid (1.60 g, 8.24 mmol, 1.00 eq.) and 1-butanol (1.602 g, 21.6 mmol, 2.62 eq.) were added to a round-bottom flask. A catalytic amount (3 drops) of concentrated sulfuric acid was added. The round-bottomed flask was attached to a Dean-Stark apparatus. The mixture was heated to vigorous reflux for 24 h. Afterwards, the mixture was cooled to room temperature and washed with equal amounts of sodium bicarbonate (sat.), sodium hydroxide solution (aq., 1 M) and water. The organic layer was dried over Na_2SO_4 and evaporated under reduced pressure. The product was obtained as a yellow oil and purified by column chromatography.

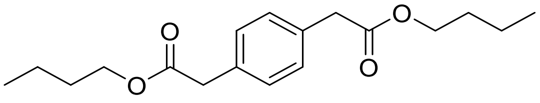

Dibutyl 2,2'-(1,4-phenylene)bisacetate **1b**. Yield 89%. ^{1}H NMR (300 MHz, Chloroform-*d*) δ 7.24 (s, 4H), 4.08 (t, *J* = 6.7 Hz, 4H), 3.59 (s, 4H), 1.67 – 1.51 (m, 4H), 1.42 – 1.27 (m, 4H), 0.91 (t, *J* = 7.3 Hz, 6H). ^{13}C NMR (75 MHz, $CDCl_3$) δ 171.77, 133.08, 129.55, 64.92, 41.22, 30.74, 19.22, 13.82. HRMS (FD, *m/z*): calculated for $C_{18}H_{26}O_4$: 306.1831, found: 306.1838.

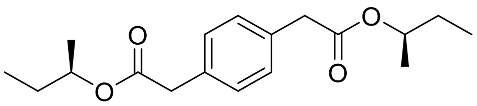

Di R-(-)-2-butyl 2,2'(1,4-phenylene) bis acetate **1c**. Procedure as above but using R-(-)-2-butanol. Yield 65%. ^{1}H NMR (300 MHz, Chloroform-*d*) δ 7.24 (s, 4H), 4.84 (h, *J* = 6.4 Hz, 2H), 3.57 (s, 4H), 1.66 – 1.42 (m, 4H), 1.18 (d, *J* = 6.3 Hz, 6H), 0.84 (t, *J* = 7.5 Hz, 6H). ^{13}C NMR (75 MHz, $CDCl_3$) δ 171.40, 133.20, 129.48, 72.87, 41.57, 28.90, 19.55, 9.75. HRMS (FD, *m/z*): calculated for $C_{18}H_{26}O_4$: 306.1831, found: 306.1872.

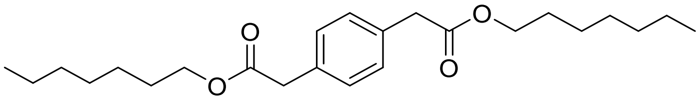

Diheptyl 2,2'-(1,4-phenylene)bisacetate **1d**. Procedure as above but using 1-heptanol. Yield 78%. ^{1}H NMR (400 MHz, Chloroform-*d*) δ 7.24 (s, 4H), 4.07 (t, *J* = 6.7 Hz, 5H), 3.59 (s, 4H), 1.60 (p, *J* = 6.9 Hz, 4H), 1.42 – 1.19 (m, 16H), 0.87 (t, *J* = 7.1 Hz, 6H). ^{13}C NMR (101 MHz, $CDCl_3$) δ 171.75, 133.11, 129.56, 65.22, 41.25, 31.85, 29.01, 28.71, 25.95, 22.71, 14.21. HRMS (FD, *m/z*): calculated for $C_{24}H_{38}O_4$: 390.2770, found: 390.2803.

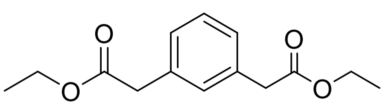

Diethyl 2,2'-(1,3)phenylenebisacetate **1e**. Prepared according to previously published procedure.[1] ^{1}H NMR (400 MHz, Chloroform-*d*) δ 7.37 – 7.26 (m, 1H), 7.23 – 7.20 (m, 3H), 4.17 (q, *J* = 7.1 Hz, 4H), 3.62 (s, 4H), 1.27 (t, *J* = 7.1 Hz, 6H).

Diazo transfer reactions.[3]

An acetonitrile solution (15 mL) of *p*-acetamidobenzenesulfonyl azide (2.1 eq.), DBU (2.1 eq.) and dibutyl 2,2'-(1,4-phenylene)bisacetate **1a** (0.851 g, 3.40 mmol) were added to a round bottom flask. The mixture was stirred at room temperature for 16 h. A saturated ammonium chloride (20 mL) solution was added. The mixture was extracted with DCM (3x15 mL). The organic layer was dried over Na_2SO_4 and evaporated under reduced pressure. The crude product was further purified using column chromatography (Silica-60) with a 98:2 heptane/ethyl acetate eluent. The product was obtained as an orange solid.

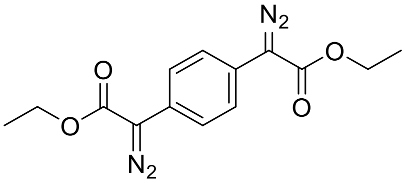

Diethyl 2,2’-(1,4)phenylenebis(2-diazoacetate) **2a**. Yield 62%. ^{1}H NMR (400 MHz, Chloroform-*d*) δ 7.51 (s, 4H), 4.33 (q, *J* = 7.4 Hz, 4H), 1.35 (t, *J* = 7.0 Hz, 6H).

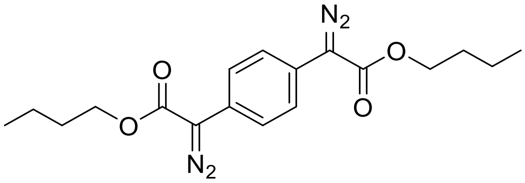

Dibutyl-(1,4-phenylene)bis(2-diazoacetate) **2b.** Yield 41%. ^{1}H NMR (300 MHz, Chloroform-*d*) δ 7.50 (s, 4H), 4.28 (t, *J* = 6.6 Hz, 4H), 1.69 (dq, *J* = 8.4, 6.7 Hz, 4H), 1.51 – 1.33 (m, 4H), 0.96 (t, *J* = 7.4 Hz, 6H). ^{13}C NMR (75 MHz, $CDCl_3$) δ 165.40, 124.53, 122.99, 65.06, 30.99, 19.28, 13.87. HRMS (FD, *m/z*): calculated for $C_{18}H_{22}N_4O_4$: 358.1641, found: 358.1646.

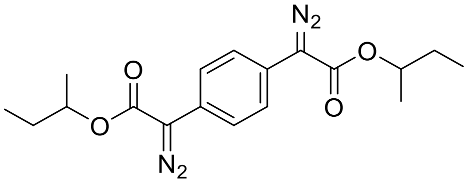

Di-R-(-)-2-butyl-(1,4-phenylene)bis(2-diazoacetate) **2c**. Yield 46%. ^{1}H NMR (300 MHz, Chloroform-*d*) δ 7.50 (s, 4H), 5.04 (h, *J* = 6.3 Hz, 2H), 1.82 – 1.56 (m, 4H), 1.30 (d, *J* = 6.3 Hz, 6H), 0.94 (t, *J* = 7.4 Hz, 6H). ^{13}C NMR (75 MHz, $CDCl_3$) δ 165.08, 124.51, 124.51, 123.07, 73.41, 29.08, 19.86, 9.79. HRMS (FD, *m/z*): calculated for $C_{18}H_{22}N_4O_4$: 358.1641, found: 358.1651.

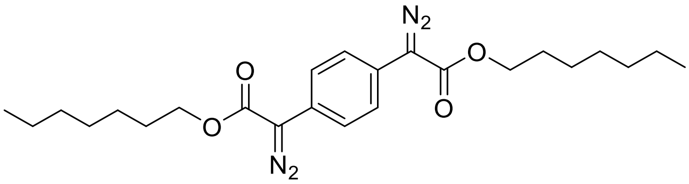

Diheptyl-(1,4-phenylene)bis(2-diazoacetate*)* **2d**. Yield 56%. ^{1}H NMR (300 MHz, Chloroform-*d*) δ 7.50 (s, 4H), 4.27 (t, *J* = 6.7 Hz, 4H), 1.70 (p, *J* = 7.0 Hz, 4H), 1.42 – 1.23 (m, 16H), 0.88 (t, *J* = 6.9 Hz, 6H).^{13}C NMR (75 MHz, $CDCl_3$) δ 165.38, 124.51, 122.98, 65.35, 63.48, 31.83, 29.02, 28.92, 25.96, 22.70, 14.19. HRMS (FD, *m/z*): calculated for $C_{18}H_{22}N_4O_4$: 442.2580, found: 442.2763.

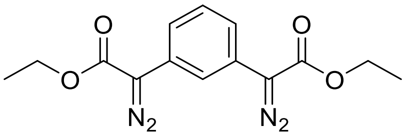

Diethyl 2,2'-(1,3-phenylene)bis(2-diazoacetate) (**2e**). Yield 36%. ^{1}H NMR (300 MHz, Chloroform-*d*) δ 7.76 (t, *J* = 1.9 Hz, 1H), 7.40 (dd, *J* = 8.7, 7.0 Hz, 1H), 7.32 – 7.24 (m, 2H), 4.35 (q, *J* = 7.1 Hz, 4H), 1.36 (t, *J* = 7.1 Hz, 6H). ^{13}C NMR (75 MHz, $CDCl_3$) δ 165.07, 129.45, 126.84, 121.07, 119.23, 61.18, 14.58. HRMS (FD, *m/z*): calculated for $C_{18}H_{22}N_4O_4$: 302.1015, found: 302.1003.

Synthesis of end-group functionalized polytetramethylene oxide 2f.

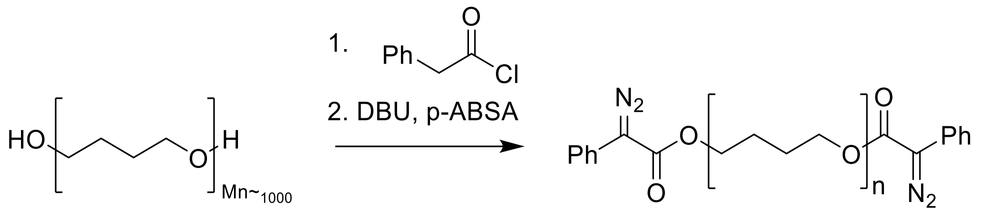

32%
Mn ~900
2f

Polytetramethylene oxide (10 g, 1000 g/mol, 10 mmol), was dissolved in DCM (15 mL) at 0 °C and triethylamine (7 mL, 50 mmol) was added. 2-Phenylacetyl chloride (3.28 mL, 25 mmol) was diluted in DCM (10 mL) and added dropwise. The ice bath was removed, and the mixture was stirred overnight. The mixture was diluted with water and pentane (25 mL) and extracted with $NaHCO_3$ (sat., 2x), NH_4Cl (sat., 2x), dilute hydrochloric acid (0.5 M, 2x) and brine after which the organic layers were dried on Na_2SO_4. The organic layers were filtered, and solvent was removed in vacuo to yield 4 g of phenylacetyl functionalized polytetramethylene oxide.

^{1}H NMR (300 MHz, $CDCl_3$) δ 7.34 – 7.24 (m, 5H), 4.11 (t, *J* = 6.4 Hz, 4H), 3.61 (s, 2H), 3.46 – 3.36 (m, polyTHF alpha), 1.62 – 1.54 (m, polyTHF beta). ^{13}C NMR (75 MHz, $CDCl_3$) δ 129.38, 128.69, 70.77, 64.90, 41.59, 26.66.

To a solution of phenylacetyl functionalized polytetramethylene oxide (*vide supra,* 1 g) and *p*-acetamidobenzenesulfonyl azide (700 mg) in anhydrous acetonitrile (10 mL) was added a solution of DBU (4.5 mL) in acetonitrile (10 mL) dropwise at 0 °C. The ice bath was removed, and the mixture was stirred overnight. Water (10 mL) was added and the resulting mixture was extracted with diethyl ether (25 mL). The organic layers were washed with $NaHCO_3$ (sat., 3x), NH_4Cl (sat., 3x), dilute hydrochloric acid (0.5 M, 3x) and brine after which the organic layers were dried on Na_2SO_4. The organic layers were filtered, and solvent was removed in vacuo to yield 2-diazo-2-phenylacetyl functionalized polytetramethylene oxide **2f** (800 mg).

^{1}H NMR (400 MHz, Chloroform-*d*) δ 7.48 (d, *J* = 7.6 Hz, 2H), 7.38 (t, *J* = 7.9 Hz, 2H), 7.18 (t, *J* = 7.4 Hz, 1H), 4.30 (t, *J* = 6.5 Hz, 2H), 3.47 – 3.37 (m, polyTHF alpha), 1.78 (dt, *J* = 8.5, 6.6 Hz, 2H), 1.72 – 1.52 (m, polyTHF beta). ^{13}C NMR (101 MHz, $CDCl_3$) δ 129.07, 125.92, 124.11, 70.88, 70.77, 70.29, 65.00, 26.66, 26.38, 25.92.

Ruthenium catalyzed polycondensation of ethyl diazo(phenyl)acetate with urea.

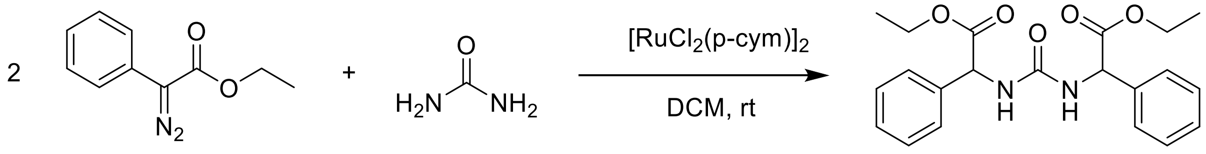

4 **5**

98% yield
60:40 dr

In a flame-dried Schlenk flask under nitrogen, urea (6 mg, 100 µmol), ethyl diazo(phenyl)acetate **4** (39 mg, 205 µmol), and dichloro(*p*-cymene)ruthenium(II) dimer (0.6 mg, 1 µmol, 1%) were suspended in DCM (2.5 mL). The solution was stirred at 800 rpm at room temperature overnight. The resulting solution was filtered over a short silica plug and eluted with ethyl acetate (5 mL). The organic layers were washed with water (5 mL), dried on Na_2SO_4 and collected in vacuo. This provided 38 mg of a 60:40 mixture of (RR/SS and RS/SR) diastereomers of **5**.

^{1}H NMR (400 MHz, DMSO) δ 7.45 – 7.30 (m, 10H), 7.11 (d, 2H), 7.03 (d, 2H), 5.26 (d, 2H), 4.08 (m, 4H), 1.13 (t, 6H) 1.10 (t, 6H). ^{13}C NMR (75 MHz, DMSO) δ 171.70, 156.63, 137.91, 137.75, 129.25, 129.21, 128.64, 128.57, 127.51, 127.45, 61.40, 61.34, 57.20, 14.37. HRMS (ESI, *m/z*): calculated for $[C_{21}H_{24}N_2O_5 + H^+]$: 358.1641, found: 358.1646, calculated for $[C_{21}H_{24}N_2O_5 + Na^+]$: 407.1577, found: 407.1591.

The diastereomers can be separated by fractional crystallization from ethyl acetate/heptane, and single crystals of the RR/SS diastereomers were obtained in this fashion and studied by SC-XRD which provided the following structure (Figure S1).

Single Crystal XRD

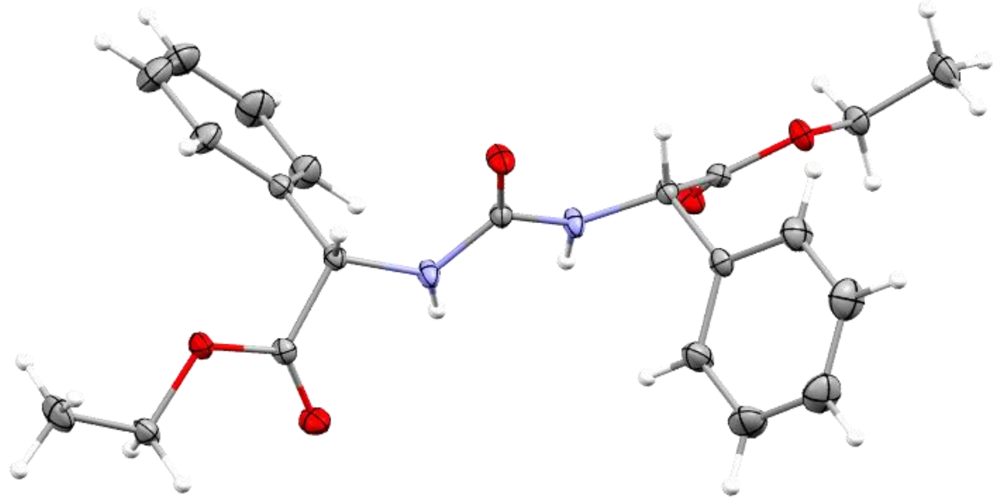

Figure S1. ORTEP diagram of single-crystal structure of disubstituted urea 5. Thermal ellipsoids drawn at 50% probability level.

SC–XRD data were collected from a shock-cooled single crystal at 100(2) K on a Bruker D8 Quest ECO three-circle diffractometer with a Sealed Tube using a TRIUMPH graphite monochromator as monochromator and a Bruker PHOTON III detector. The diffractometer was equipped with an Oxford Cryostream 700 low temperature device and used MoKα radiation (λ = 0.71073 Å). All data were integrated with SAINT and a multi-scan absorption correction using SADABS was applied.[4,5] The structure was solved by direct methods using SHELXT and refined by full-matrix least-squares methods against F2 by SHELXL-2018/3.[6,7] All non-hydrogen atoms were refined with anisotropic displacement parameters. All hydrogen atoms were refined with isotropic displacement parameters. The urea N-H was refined freely, with the rest on calculated positions using a riding model with their Uiso values constrained to 1.5 times the Ueq of their pivot atoms for terminal sp^3 carbon atoms and 1.2 times for all other carbon atoms. Bijvoet-pair analysis by PLATON[8] (version 100822) confirmed the presence of a perfect inversion twin. Validation by CheckCIF revealed no A- or B-level alerts. Crystallographic data for the structures reported in this paper have been deposited with the Cambridge Crystallographic Data Centre. CCDC 2206396 contain the supplementary crystallographic data for this paper. These data can be obtained free of charge from The Cambridge Crystallographic Data Centre via www.ccdc.cam.ac.uk/structures

Table S1. Crystal data and structure refinement for 2206396

CCDC number	2206396
Empirical formula	$C_{21}H_{24}N_2O_5$
Formula weight	384.42
Temperature [K]	100(2)
Crystal system	monoclinic
Space group (number)	$C2$ (5)
a [Å]	18.007(4)
b [Å]	4.7823(9)
c [Å]	11.658(2)
α [°]	90
β [°]	105.701(8)
γ [°]	90
Volume [Å^3]	966.4(3)
Z	2
ρ_{calc} [gcm^{-3}]	1.321
μ [mm^{-1}]	0.095
F(000)	408
Crystal size [mm^3]	0.631×0.095×0.076
Crystal colour	colourless
Crystal shape	prism
Radiation	MoK_α (λ=0.71073 Å)
2θ range [°]	6.67 to 66.42 (0.65 Å)
Index ranges	−27 ≤ h ≤ 27<br>−7 ≤ k ≤ 7<br>−17 ≤ l ≤ 17
Reflections collected	88428
Independent reflections	3709<br>R_{int} = 0.0558<br>R_{sigma} = 0.0192
Completeness to θ = 25.242°	99.4 %
Data / Restraints / Parameters	3709/1/132
Goodness-of-fit on F^2	1.116
Final R indexes [I≥2σ(I)]	R_1 = 0.0349<br>wR_2 = 0.0915
Final R indexes [all data]	R_1 = 0.0378<br>wR_2 = 0.0936
Largest peak/hole [eÅ^{-3}]	0.34/-0.24
Flack X parameter	0.5

Ruthenium catalyzed polycondensation of bis-diazos 2a-e with urea.

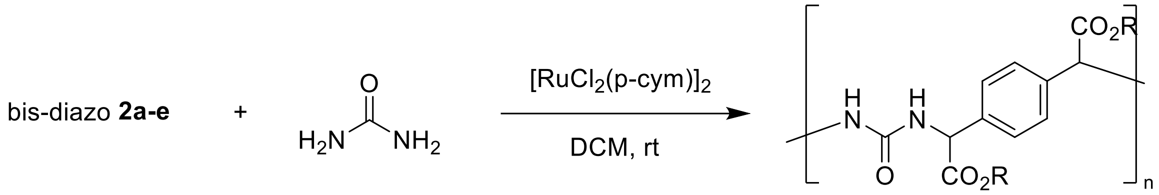

In a flame-dried Schlenk flask under nitrogen, urea (6 mg, 100 µmol), bis-diazo (100 µmol), and dichloro(*p*-cymene)ruthenium(II) dimer (1.6 mg, 2.5 µmol, 5% Ru/bis-diazo) were suspended in DCM (2.5 mL). The solution was stirred at 800 rpm at room temperature for 1 hour. The clear mixture was added to hexane (20 mL), resulting in precipitation of the polymer, and the suspension was centrifuged. The supernatant was decanted and the solid was resuspended by sonicating in water (20 mL) after which the suspension was centrifuged again. The supernatant was decanted and the solid was resuspended by sonicating in diethyl ether (20 mL) after which the suspension was centrifuged again. The supernatant was again decanted and the solid was dried under vacuum at 90 °C for 16 hours. *Note: as this reaction is heterogeneous the stirring speed, reaction vessel and urea particle size influence the total reaction time.*

1,4-ethyl polyurea **3a.** Yield 56%. ^{1}H NMR (500 MHz, DMSO-d_6) δ 7.35 (m, 4H), 6.98 (d, *J* = 90.3 Hz, 2H), 5.29 (s, 2H), 4.10 (s, 4H), 1.13 (s, 6H). ^{13}C NMR (126 MHz, DMSO-d_6) δ 170.45 (-CO_2-), 155.79 (NH-*CO*-NH), 137.07 (C_{Ar}-CH-), 126.91 (C_{Ar}-H), 60.49 (-CO_2CH_2-), 56.22 (C_{Ar}-*CH*-), 13.40 (CH_2-CH_3).

1-Butyl polyurea **3b**. Yield 65%. ^{1}H NMR (500 MHz, DMSO-d_6) δ 7.34 (m, 4H), 6.99 (d, *J* = 90.3 Hz, 2H), 5.29 (s, 2H), 4.04 (s, 4H), 1.48 (s, 4H), 1.22 (s, 4H), 0.80 (s, 6H). ^{13}C NMR (126 MHz, DMSO-d_6) δ 170.59 (-CO_2-), 155.73 (NH-*CO*-NH), 137.09 (C_{Ar}-CH-), 126.84 (C_{Ar}-H), 64.16 (-CO_2CH_2-), 56.29 (C_{Ar}-*CH*-), 29.63 (CH_2-CH_2-CH_2-CH_3), 17.91 (CH_2-CH_2-CH_2-CH_3), 12.78 (CH_2-CH_2-CH_2-CH_3). ^{15}N NMR (51 MHz, DMSO-d_6) δ 88.74 (d, *J* = 89.9 Hz).

2-butyl polyurea **3c**. Yield 45%. ^{1}H NMR (300 MHz, DMSO-d_6) δ 7.38, 7.36, 6.99, 5.27, 4.75, 2.51, 1.53, 1.40, 1.17, 1.01, 0.82, 0.61. ^{13}C NMR (75 MHz, DMSO) δ 171.00, 156.67, 137.09, 130.54, 130.23, 127.66, 127.59, 126.45, 76.04, 75.63, 73.36, 57.35, 57.29, 29.35, 28.52, 19.52, 19.36, 19.11, 9.55, 9.27.

1-heptyl polymer **3d**. Yield 57%. ^{1}H NMR (400 MHz, DMSO-d_6) δ 7.35, 7.33, 7.31, 6.99, 6.99, 6.97, 5.29, 4.03, 1.51, 1.21, 0.85. ^{13}C NMR (101 MHz, DMSO-d_6) δ 170.48, 155.74, 137.09, 126.79, 64.41, 56.24, 30.58, 27.60, 24.62, 21.41, 13.23.

1,3-ethyl polymer **3e**. Yield 82%. ^{1}H NMR (300 MHz, DMSO-d_6) δ 7.36, 7.33, 7.31, 7.06, 5.31, 4.13, 4.10, 1.17, 1.16, 1.14, 1.13, 1.13, 1.11, 1.10. ^{13}C NMR (75 MHz, DMSO-d_6) δ 170.54, 170.47, 155.82, 137.71, 128.69, 126.27, 125.50, 60.55, 56.44, 13.41.

^{1}H NMR (400 MHz, Chloroform-*d*) δ 7.48 (d, *J* = 7.6 Hz, 2H), 7.38 (t, *J* = 7.9 Hz, 2H), 7.18 (t, *J* = 7.4 Hz, 1H), 4.30 (t, *J* = 6.5 Hz, 2H), 3.47 – 3.37 (m, polyTHF alpha), 1.78 (dt, *J* = 8.5, 6.6 Hz, 2H), 1.72 – 1.52 (m, polyTHF beta).

Copolymer **3g** ^{1}H NMR (500 MHz, DMSO) δ 7.35 (aromatic), 7.04 (N-H), 5.28 (benzylic C-H), 4.05 (CH_2-O), 3.35 (polyTHF alpha), 1.53 (polyTHF beta), 1.22 (CH_3).

Gravimetric determination of the solubility of urea in DCM

Urea (100 mg) was suspended in anhydrous or water-saturated DCM (10 mL) and sonicated for 2 hours to provide a saturated solution of urea in DCM. The suspension was filtered, and 5 mL of the filtrate was added to a pre-weighed volumetric flask and evaporated *in vacuo*, after which the flask was further dried for 2 hours at 80 °C in a vacuum oven. The flask was weighed, and this process was repeated three times. This provided a lower limit (anhydrous DCM) and upper limit (water-saturated DCM) for the solubility of urea in DCM. The solubility range of urea in DCM was determined to be 0.22–2.8 mg/mL, depending on the water content of DCM.

Kinetics by in-situ ReactIR.

In-situ infrared spectra were collected with a ReactIR 15 (Mettler–Toledo, 1400003) equipped with an air-cooled Art Photonics FlexiSpec® diamond in-situ FTIR-ATR Silicon (Mettler–Toledo, 14472000) probe for harsh conditions. Conversion was followed by the normalised peak area of the $\nu_{N=N}$ of the diazo moiety (2085 cm^{-1}) with a two-point baseline from 2045 to 2120 cm^{-1}. Yield was followed by the normalised peak area of the $\nu_{C=O}$ of the ester (1737 cm^{-1}) with a two-point baseline from 1715 to 1790 cm^{-1}. One trace in the differential spectra corresponds to 2 minutes and 45 seconds.

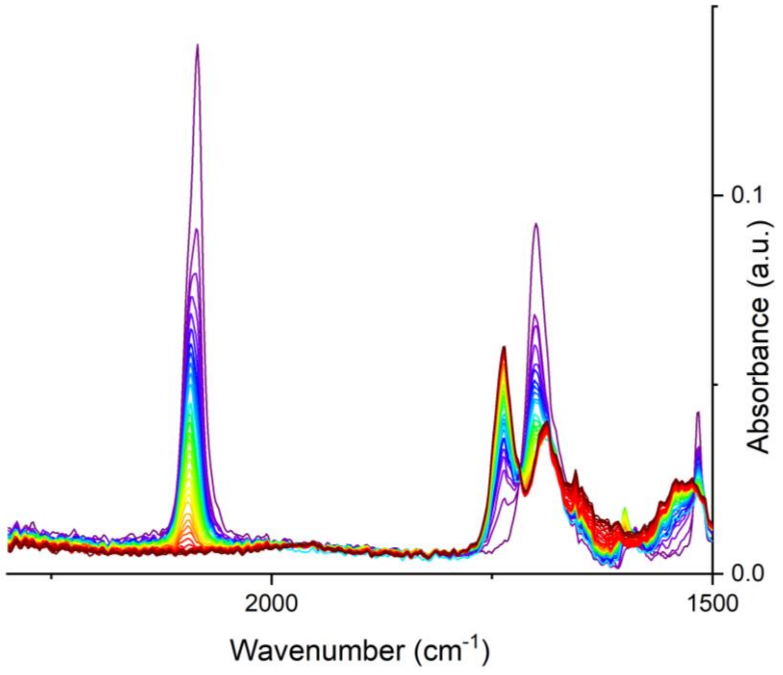

Figure S2. Differential spectra of in-situ IR monitoring of the polycondensation of 2a.

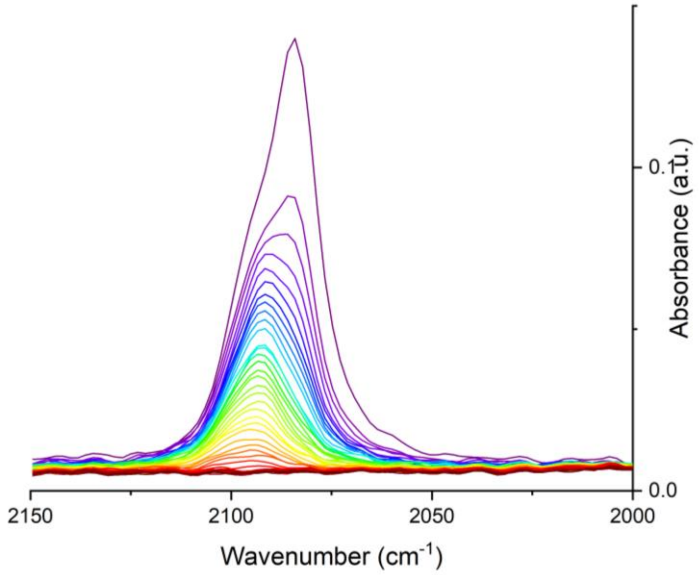

Figure S3. Zoom of diazo region during in-situ IR monitoring of the polycondensation of 2a.

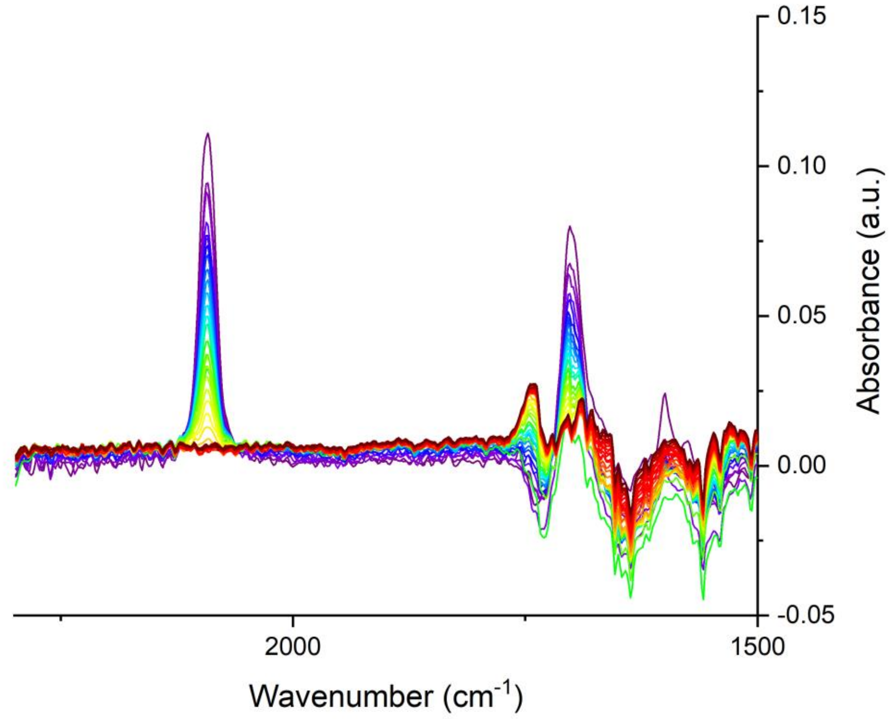

Figure S4. Differential spectra of in-situ IR monitoring of the polycondensation of 2e.

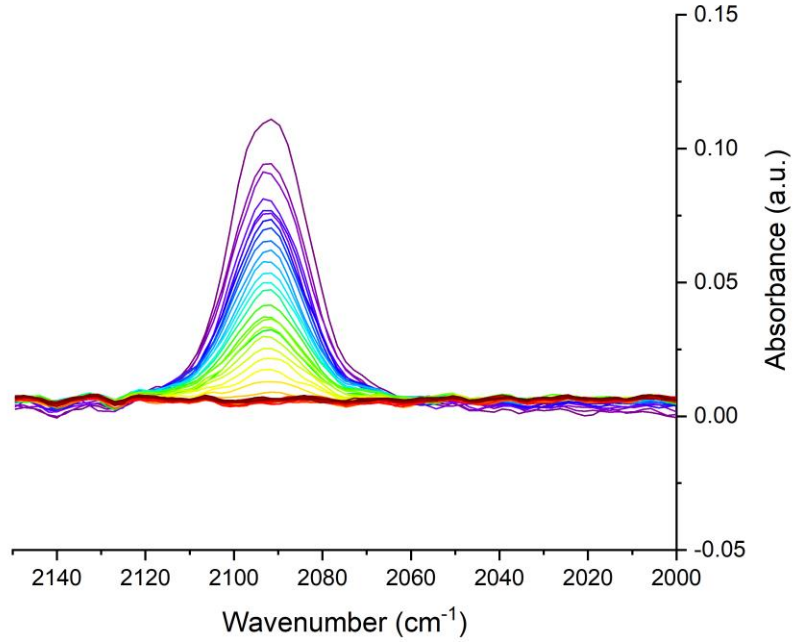

Figure S5. Zoom of diazo region during in-situ IR monitoring of the polycondensation of 2e.

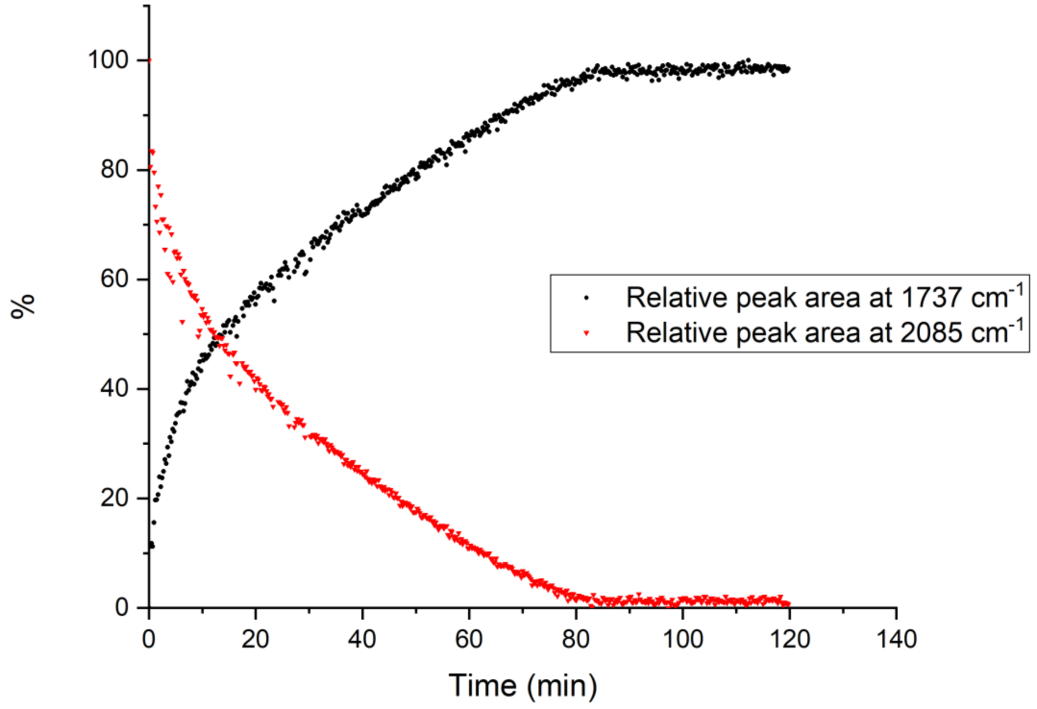

Figure S6. Relative intensity over time of in-situ IR monitoring of the polycondensation of 2a.

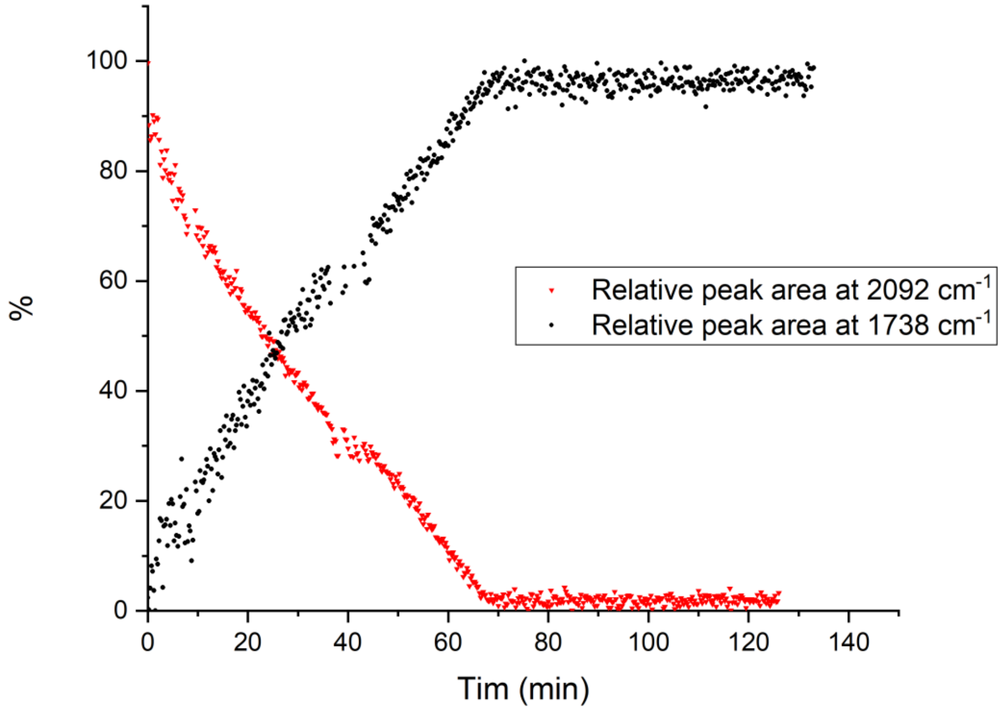

Figure S7. Relative intensity over time of in-situ IR monitoring of the polycondensation of 2e.

Kinetics by bubble counting

Method

Reaction kinetics of **2a** and **2e** were studied using a homebuilt bubble counter of which the design[9] and data processing[10] is described in detail elsewhere. Briefly, after stirring the reaction mixture at temperature, the gastight reactor was closed off and a catalyst solution was directly injected into the reaction mixture causing a small volume displacement. This, and any further gas evolution caused by dinitrogen release from the diazo monomer was detected by analysing bubble formation from a DCM medium. Bubbles were detected with the aid of a laser and translated into an evolved volume of gas.

A screwcap vial (10.0 mL) was charged with a stirring bean (8.0 x 3.0 mm), bis-diazo **2a** or **2e** (30.2 mg, 100 µmol), urea (6 mg, 100 µmol) and DCM (2.0 mL) and mounted on the reactor head. A catalyst solution (0.5 mL, 1.6-3.2 mg [Ru(*p-cym*)Cl_2]$_2$, 5-10 mol%) was loaded into a gastight syringe (1.0 mL) that was connected to the reactor head via a PEEK needle port and PEEK tubing (1/16", l = 10 cm) allowing for direct injection into the reaction mixture. Then, the other three remaining syringe ports were closed off and the catalyst solution was injected. Gas production was monitored until reaction completion.

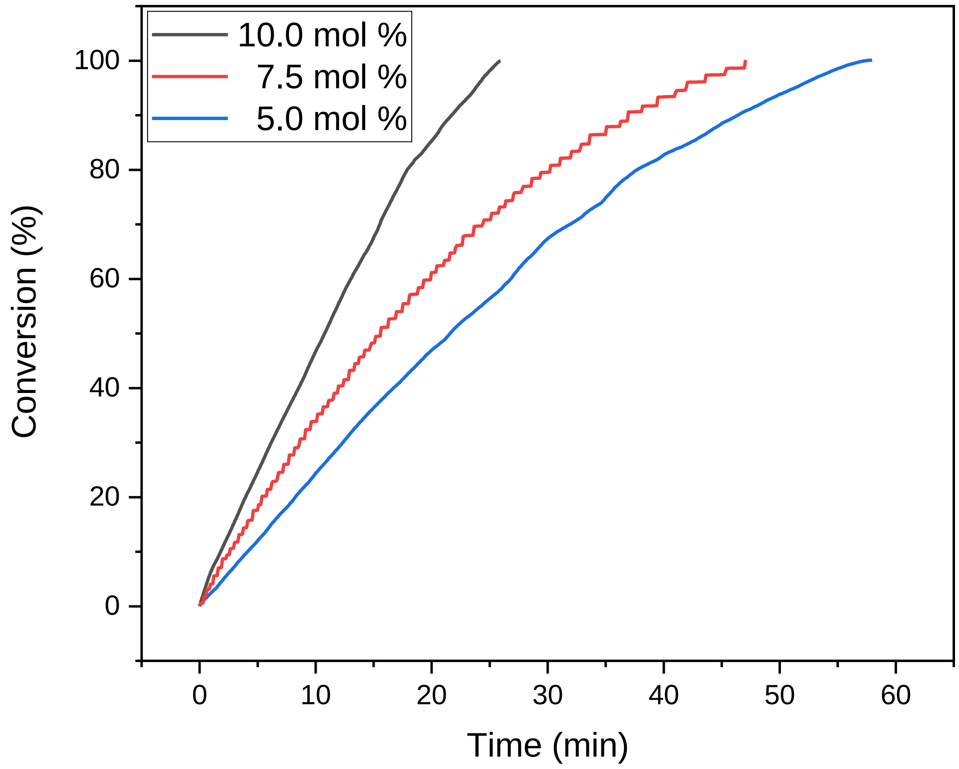

Figure S8. Conversion of diazo over time as measured by bubble counting of the polycondensation of 2e at various catalyst concentrations.

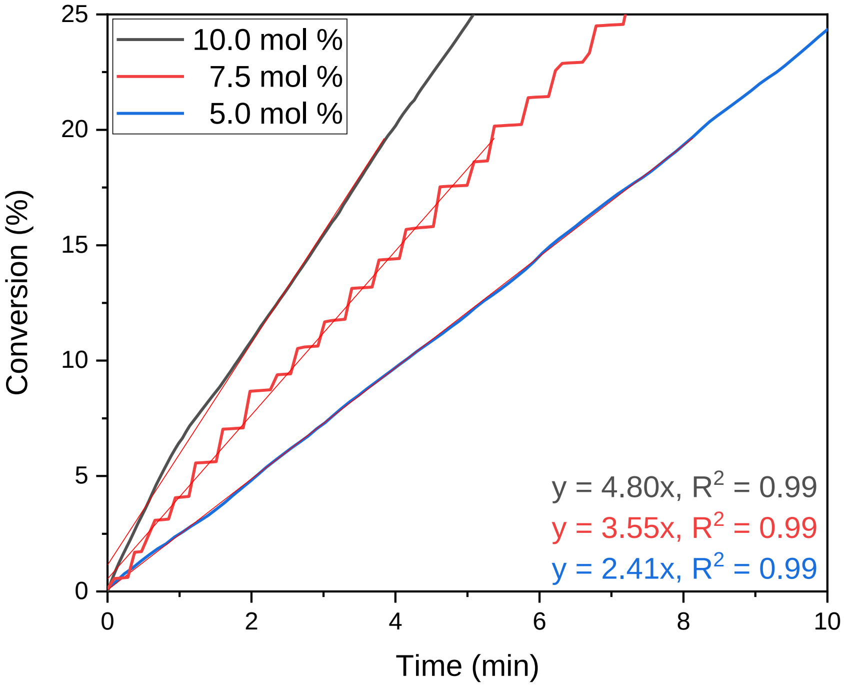

Figure S9. Zoom of initial conversion of diazo over time as measured by bubble counting of the polycondensation of 2e at various catalyst concentrations.

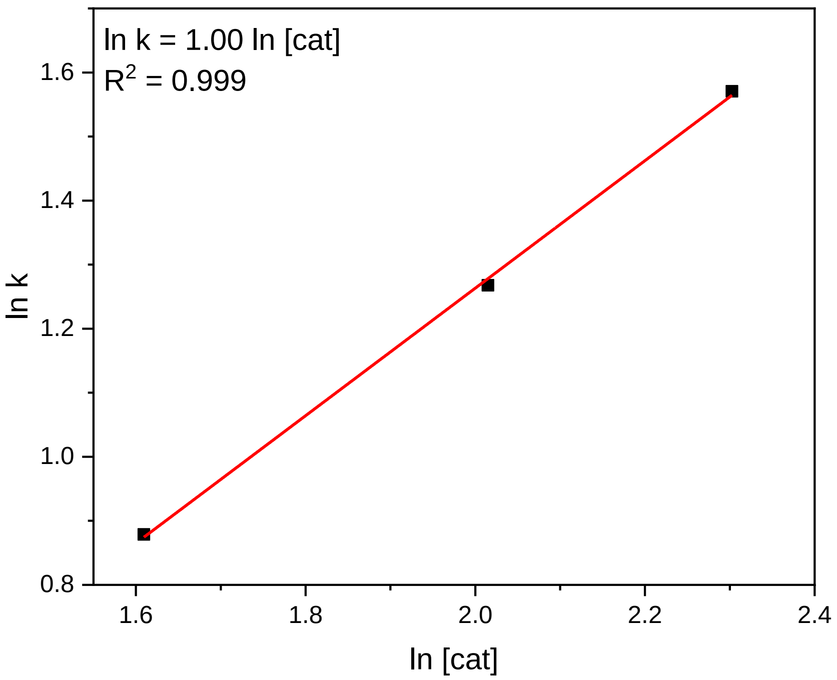

Figure S10. Log-Log plot of initial rates versus catalyst concentration of polycondensation of 2e.

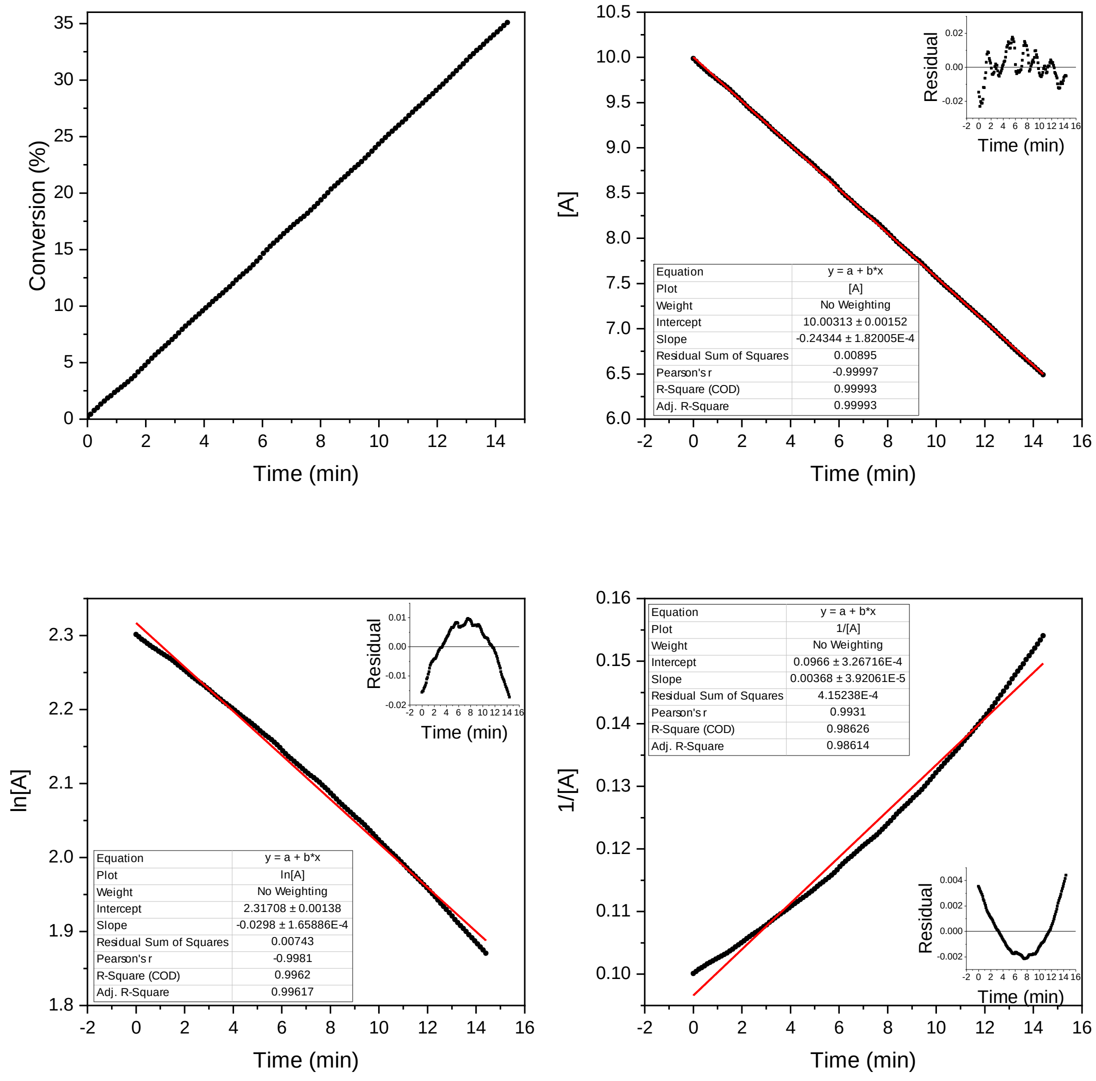

Figure S11. Zero, first and second order plots to determine the order in substrate 2e in polycondensation at 5.0 mol% catalyst, insets show residuals.

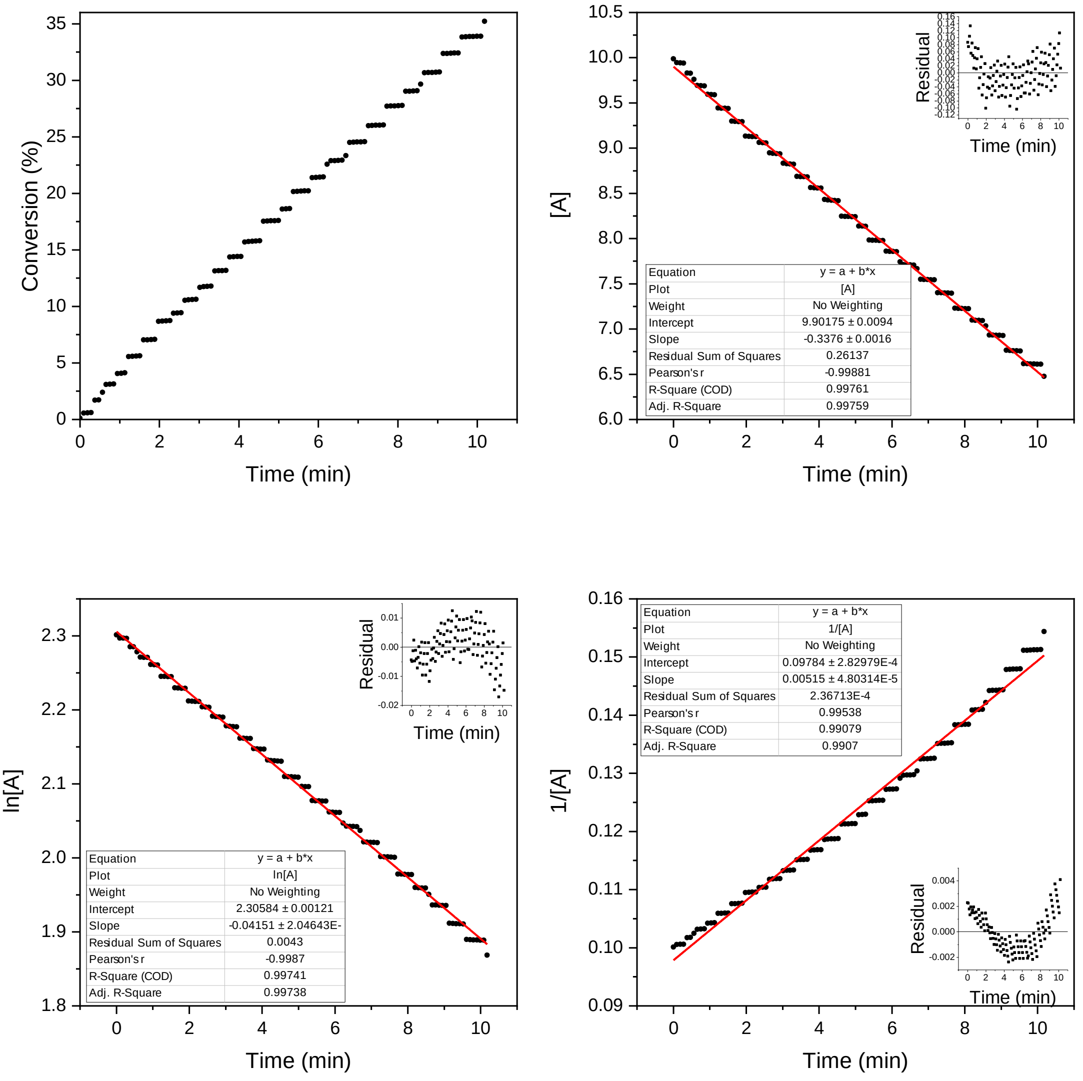

Figure S12. Zero, first and second order plots to determine the order in substrate 2e in polycondensation at 7.5 mol% catalyst, insets show residuals.

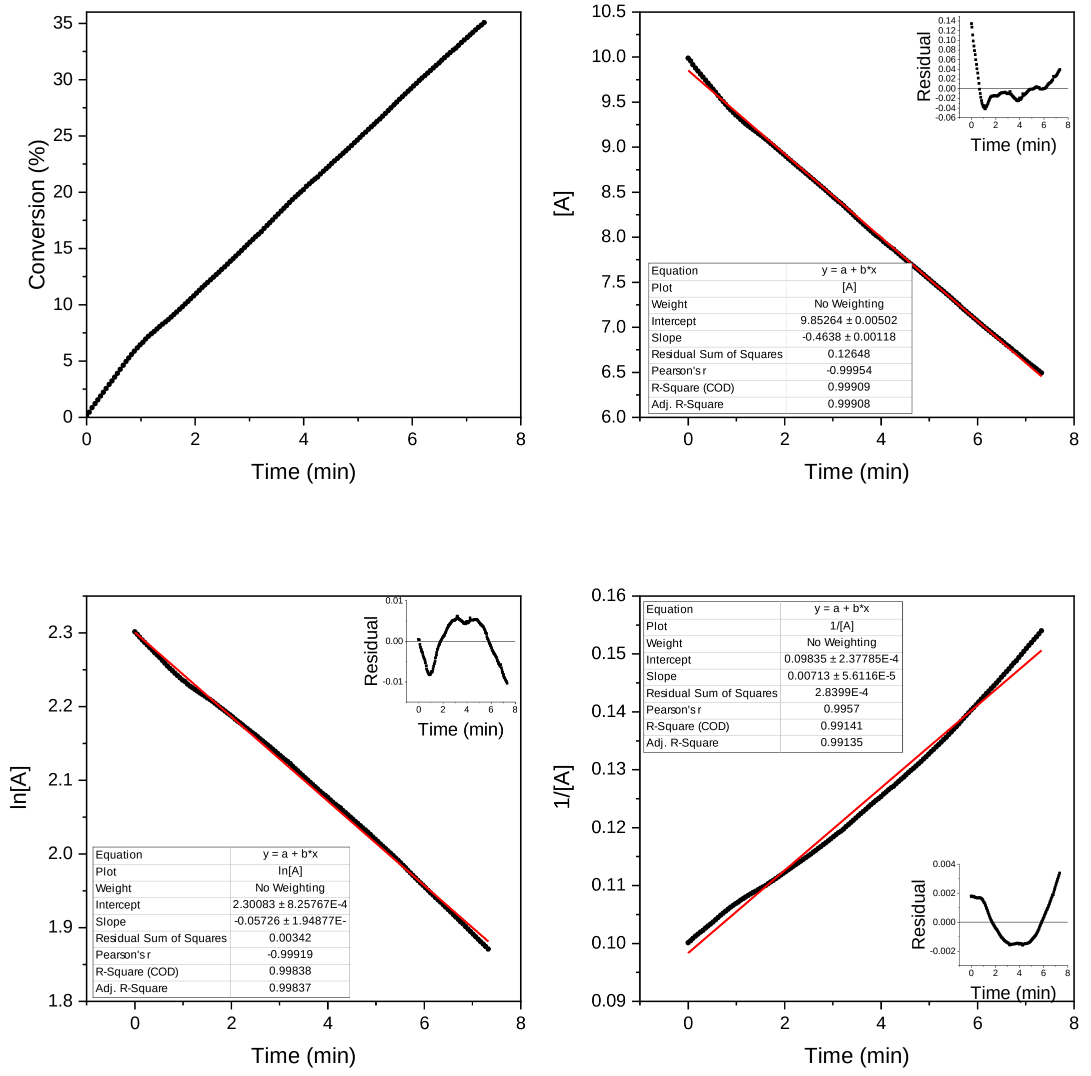

Figure S13. Zero, first and second order plots to determine the order in substrate 2e in polycondensation at 10 mol% catalyst, insets show residuals.

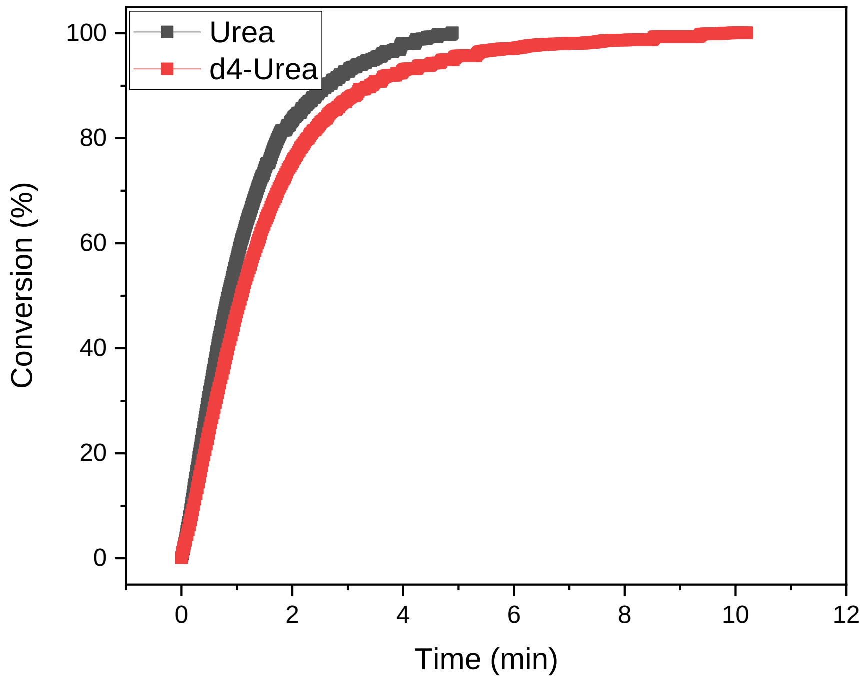

Figure S14. Conversion of diazo over time as measured by bubble counting of the polycondensation of 2e at 5% catalyst loading, using deuterated and non-deuterated urea.

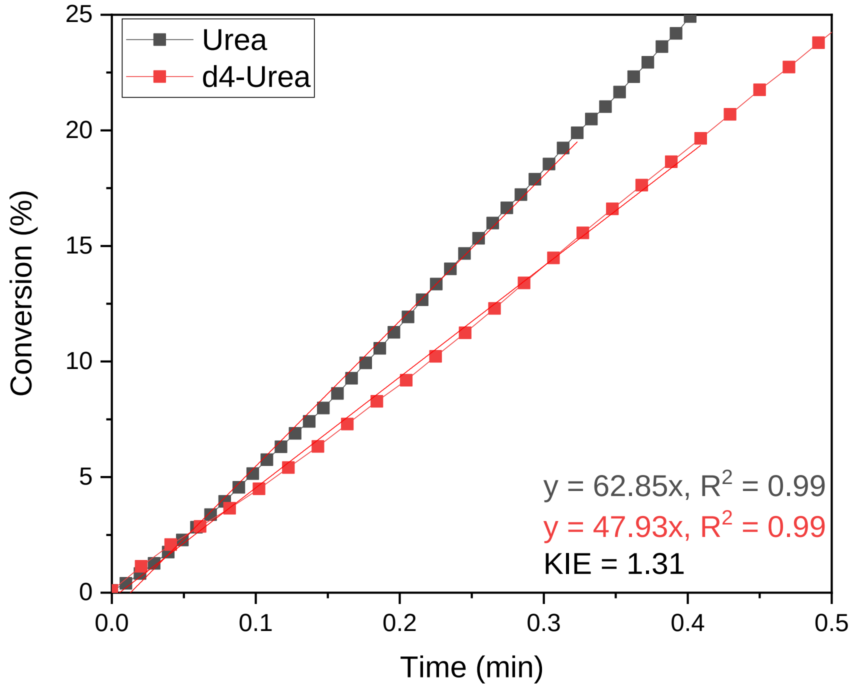

Figure S15. Initial conversion of diazo over time as measured by bubble counting of the polycondensation of 2e at 5% catalyst loading, using deuterated and non-deuterated urea.

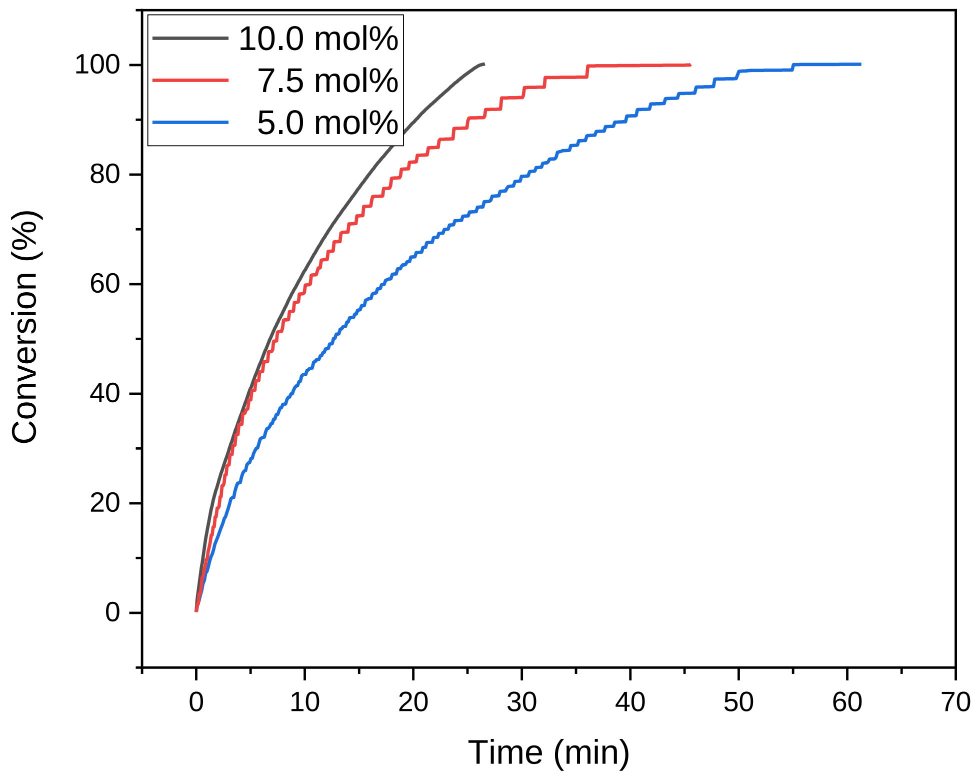

Figure S16. Conversion of diazo over time as measured by bubble counting of the polycondensation of 2a at various catalyst concentrations.

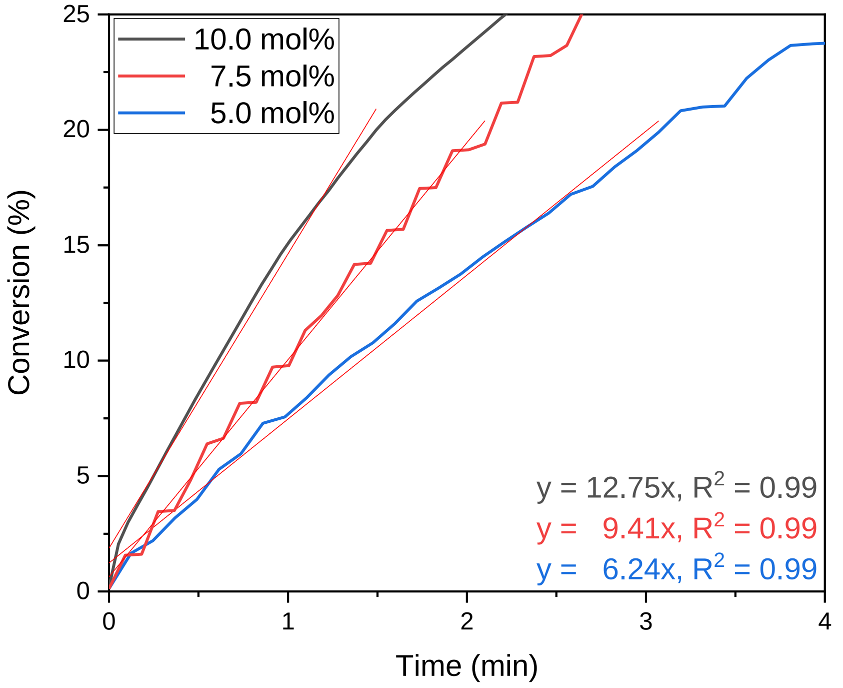

Figure S17. Zoom of initial conversion of diazo over time as measured by bubble counting of the polycondensation of 2a at various catalyst concentrations.

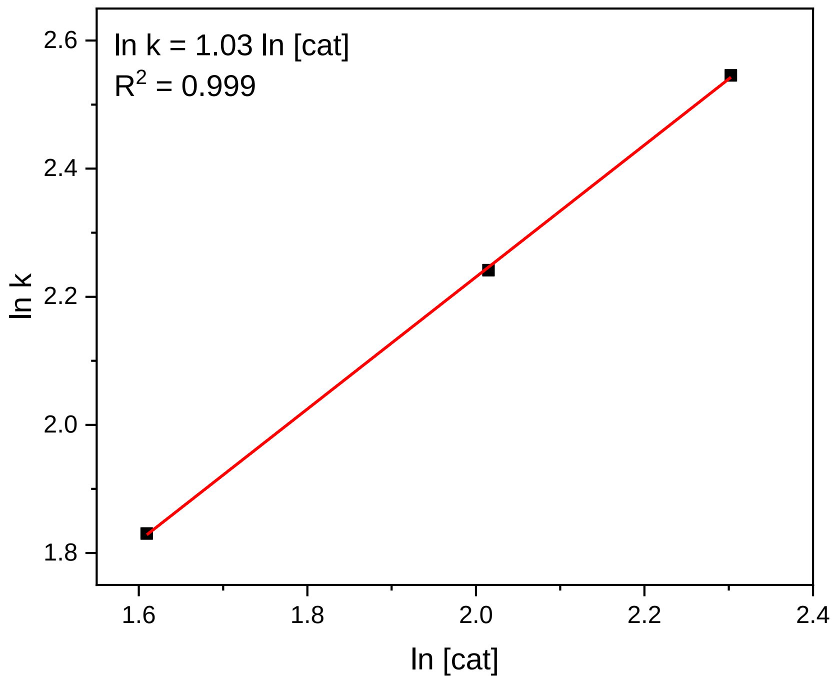

Figure S18. Log-Log plot of initial rates versus catalyst concentration of polycondensation of 2a.

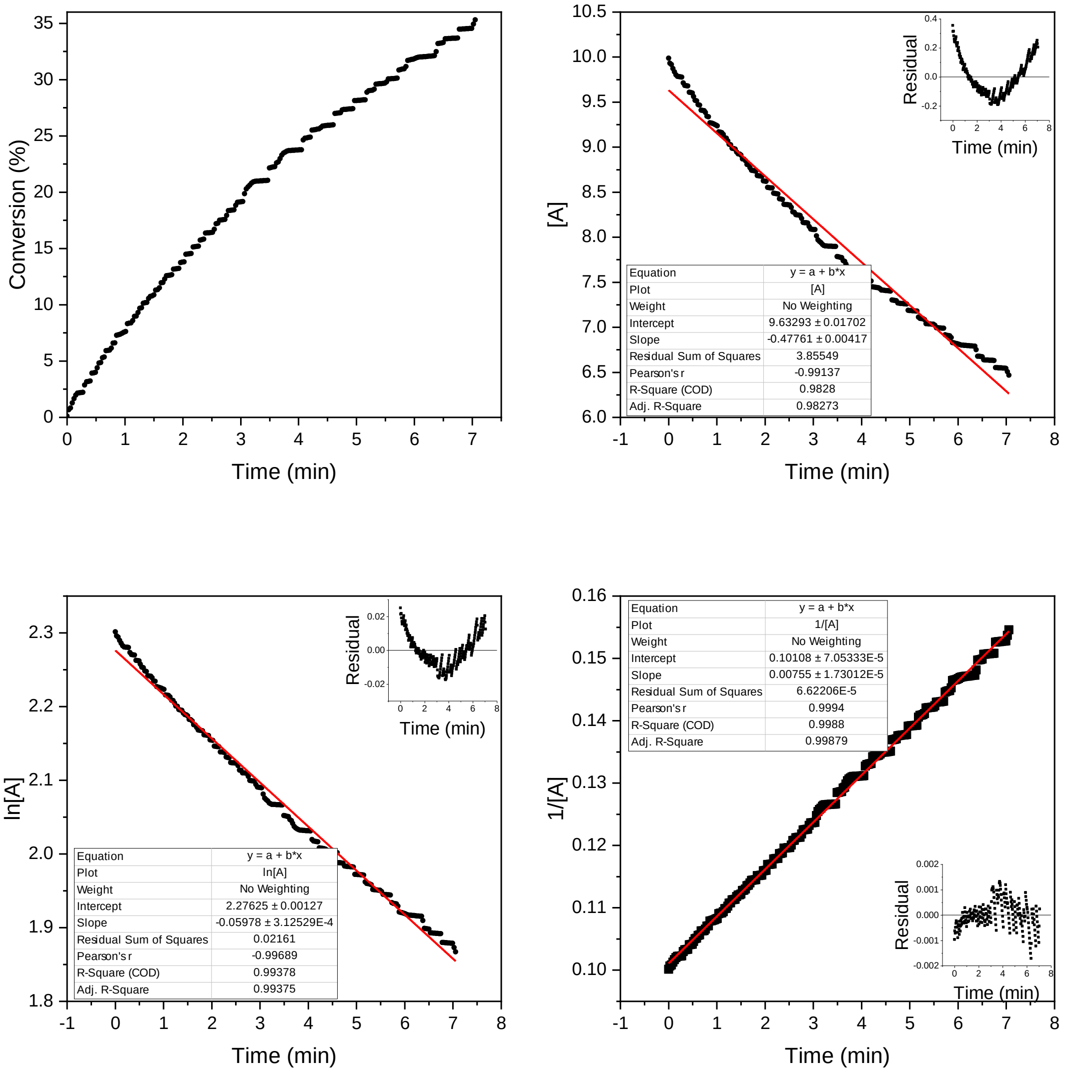

Figure S19. Zero, first and second order plots to determine the order in substrate 2a in polycondensation at 5 mol% catalyst, insets show residuals.

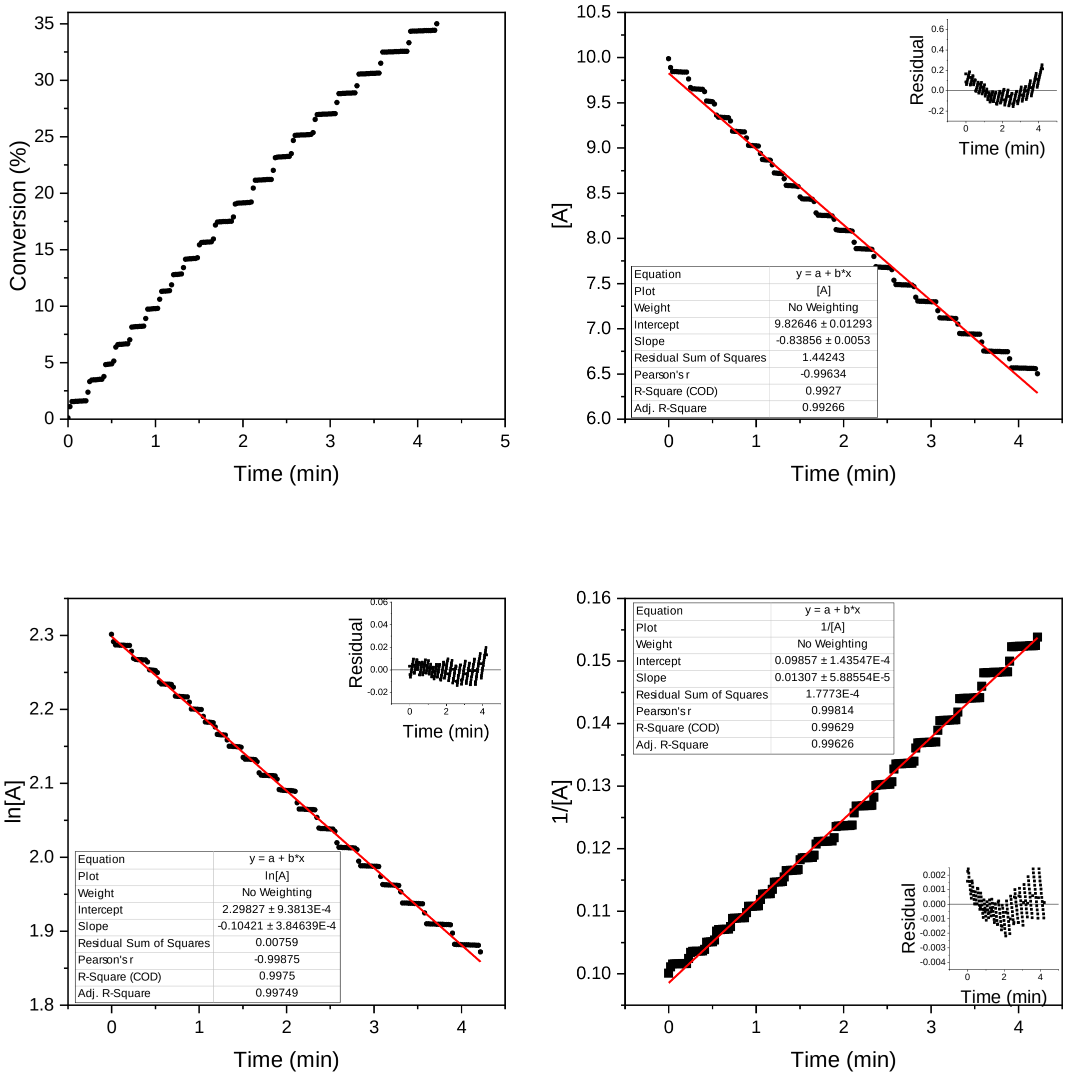

Figure S20. Zero, first and second order plots to determine the order in substrate 2a in polycondensation at 7.5 mol% catalyst, insets show residuals.

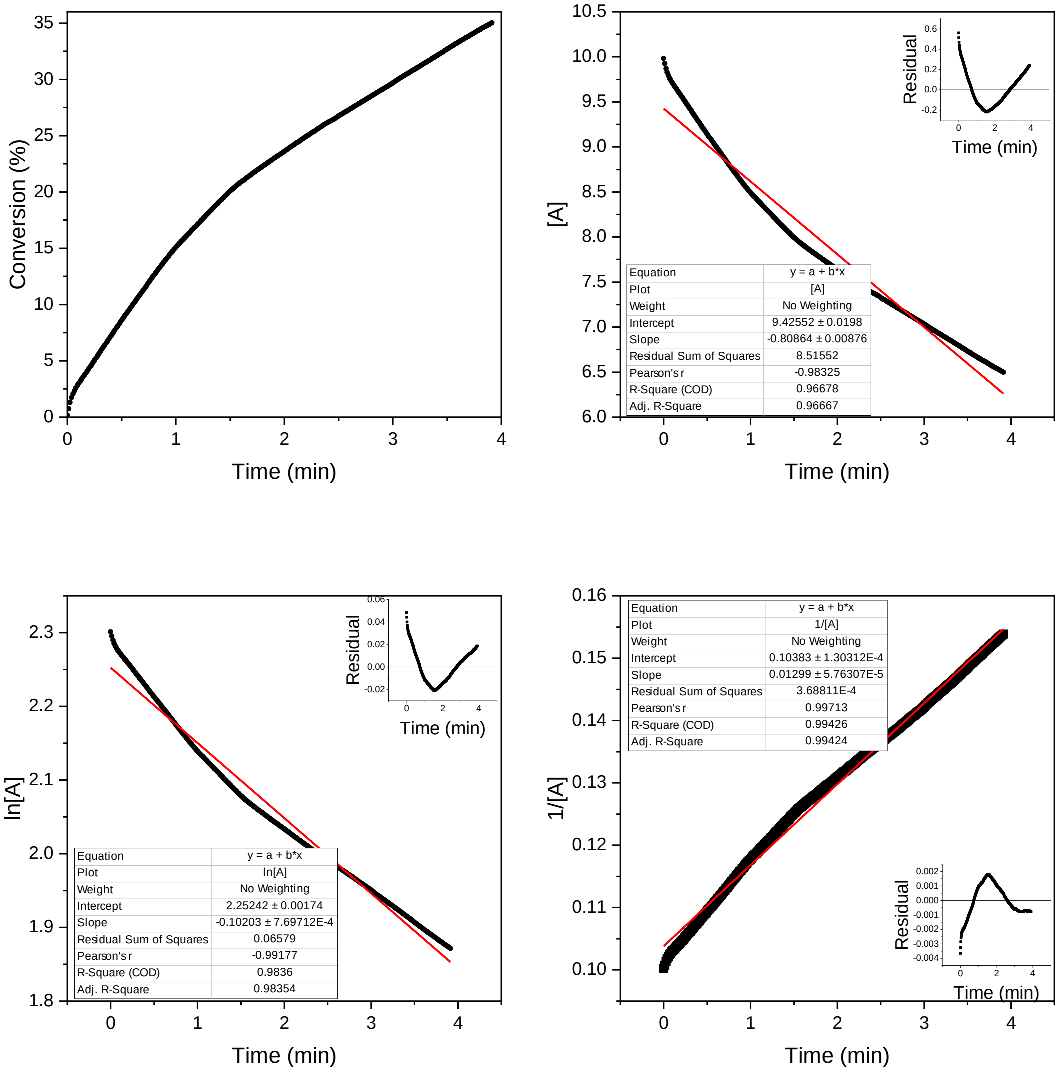

Figure S21. Zero, first and second order plots to determine the order in substrate 2a in polycondensation at 10 mol% catalyst, insets show residuals.

DSC Data

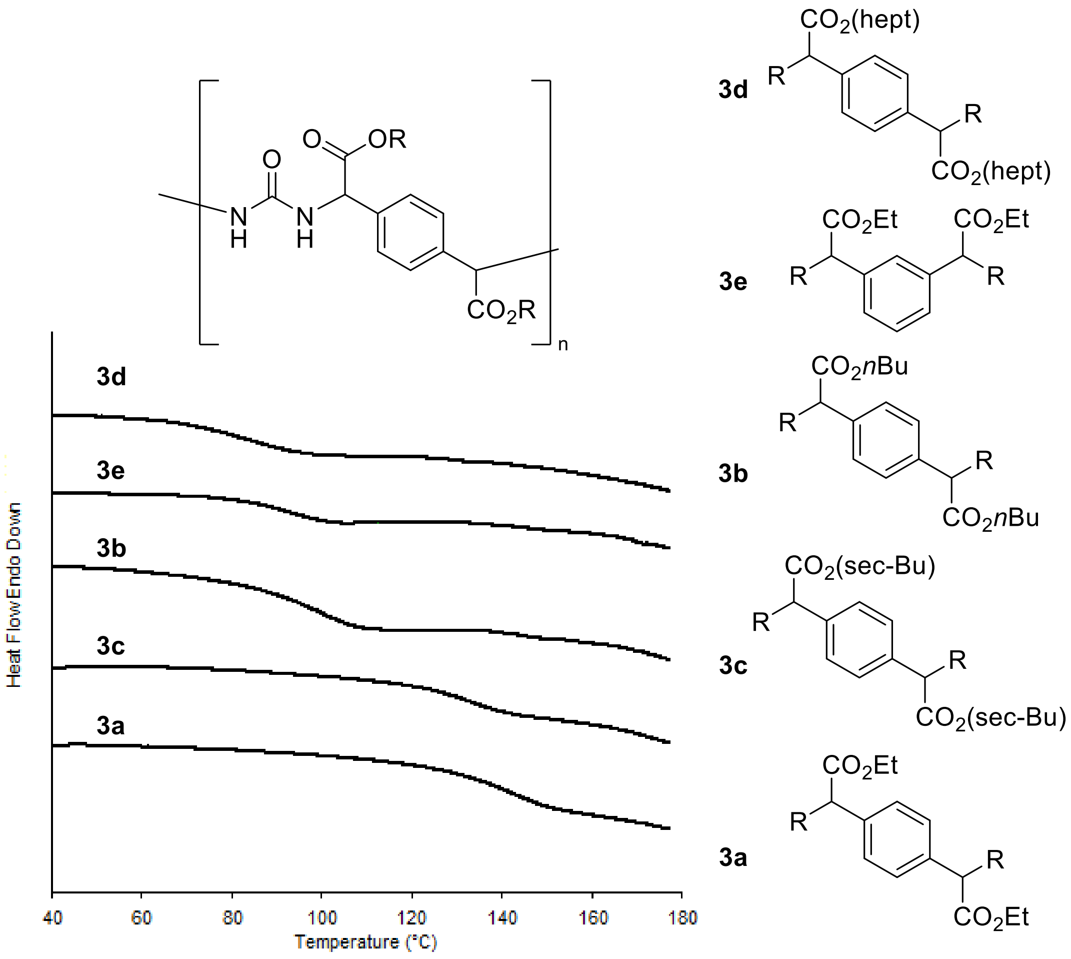

Figure S22. Second heating DSC traces of polymers 3a-e.

SEC traces and DSC data of copolymers

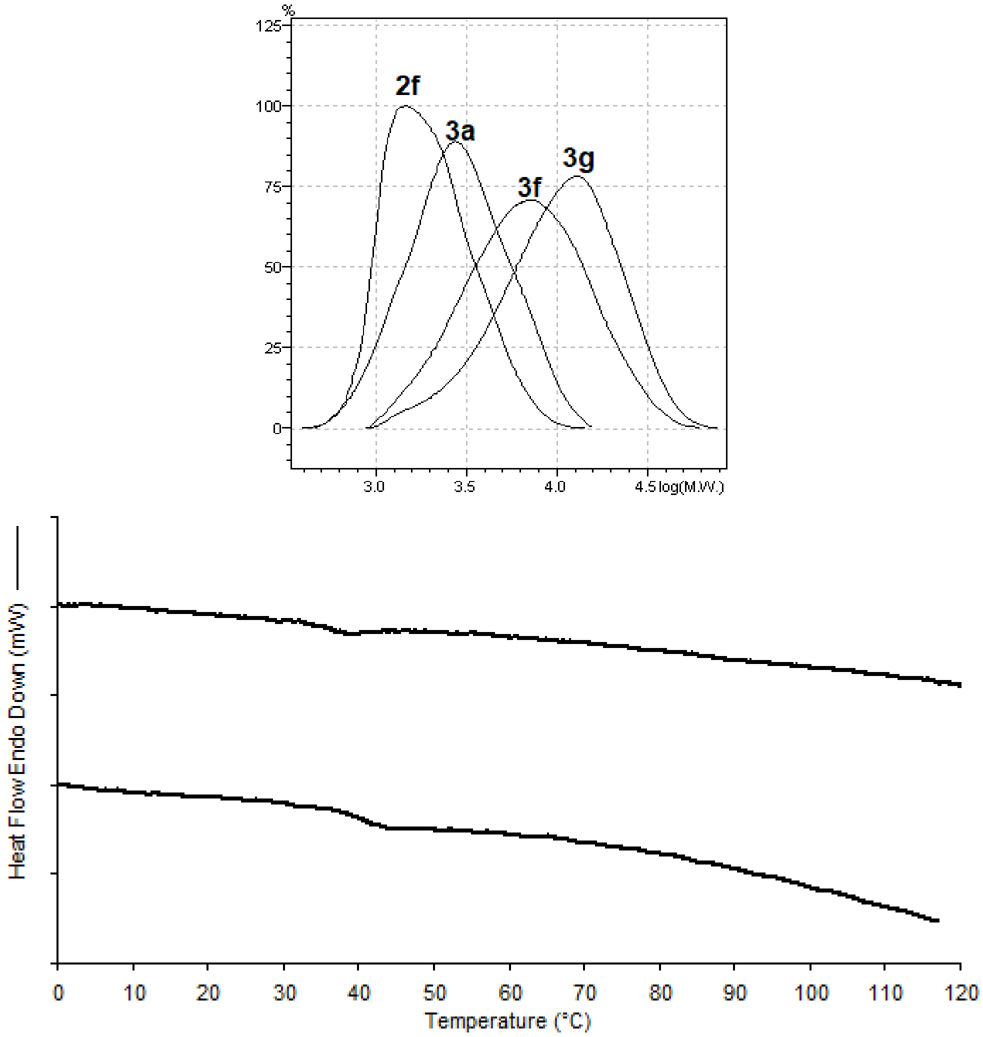

Figure S23. GPC traces of polymers 2f, 3a, 3f and 3g (top) and DSC traces of 3f and 3g (bottom).

End-group analysis by Mass Spectrometry

In order to further understand the chemical structure of the polymers, the end-groups were studied by mass spectrometry. The relatively low molecular weights obtained for these polyureas could arise from cyclization of the polymers. Indeed, when polymer **3b** was subjected to ESI-MS analysis, we observed signals corresponding to oligomers without end-groups. With increasing molecular weight (repeating unit n=5 and higher) the signals corresponding to solvent adducts of the polymers became more prevalent. MS-MS analysis confirms that these signals indeed correspond to solvent clusters of the polymers (see figure S24, S25), which still contain no end-group. The absence of end-groups strongly indicates the presence of cyclic structures in the polymer.[11] This can possibly explain the relatively low molecular weights obtained for the polymers in this study, as cyclization is a termination pathway. We envision the cyclization to happen through two possible pathways, either by direct insertion of a ruthenium carbene to a free urea on the other chain end (**A** to **C**, vide infra), or by oxidation of the ruthenium carbene and subsequent imine formation (**A** to **B** to **D**). Oxidation of diazos to ketones is known with various catalysts, and the presence of imine linkages as shown in structure **D** is supported by the observed ^{1}H-NMR signals at 4.45 ppm and 1.36 ppm. [12,13] Furthermore, the presence of these imine linkages is also supported by the observation of the n*(Repeating Unit)−2 signal in ESI-MS. However, under catalytic conditions the presence of oxygen should be minimal especially considering the constant gas evolution of nitrogen. Therefore, either this oxidation happens during workup, or the imine linkages are formed by (ruthenium-catalyzed) dehydrogenation of the polymer chain (**C** to **D**, vide infra).[14] As the ESI-MS only provided satisfactory results until around 3000 m/z, we utilized MALDI-TOF to further study the composition of our material. Indeed, until 5000 m/z the observed mass signals are in accordance with polymers containing no end-group for polymers **3a**, **3b** and **3e** (Figure S31–33). This supports the presence of cyclization as a termination pathway for polymerization.

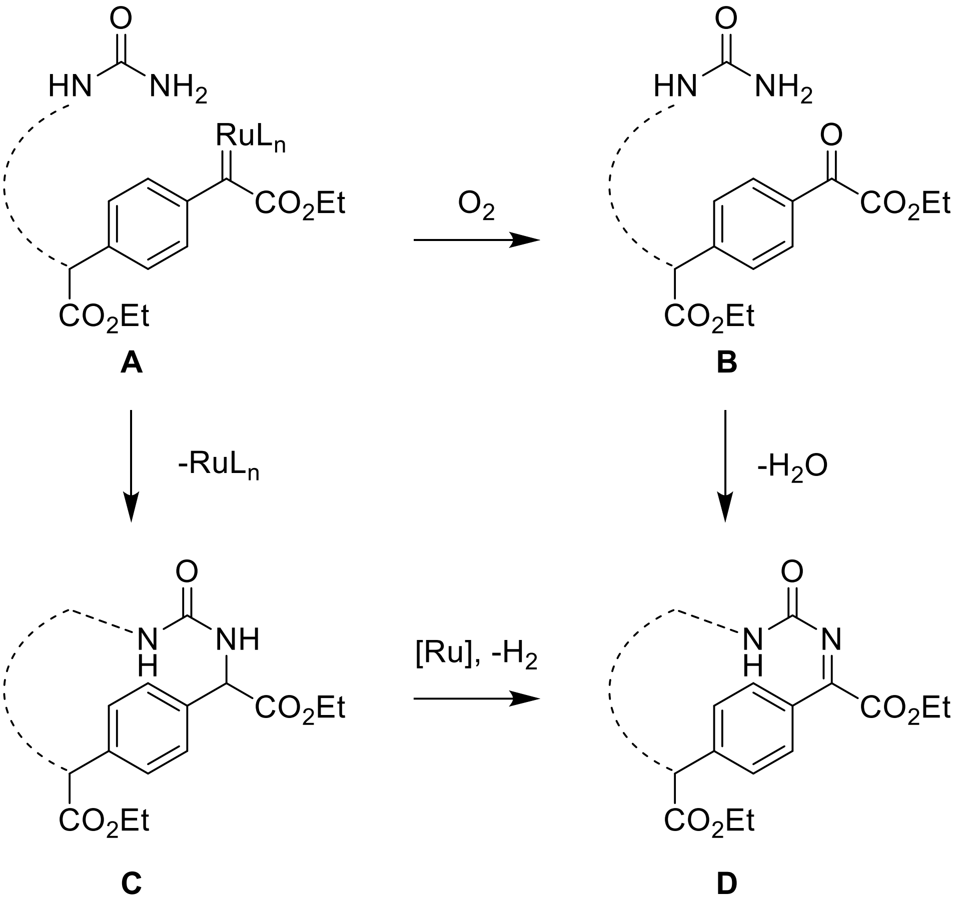

Scheme S1. Possible termination pathways through cyclization of the polymer chain.

ESI-MS

Electrospray-ionization MS (ESI-MS): Mass spectra were collected on a HR-ToF Bruker Daltonik GmbH (Bremen, Germany) Impact II, an ESI-ToF MS capable of resolution of at least 40000 FWHM. Detection was in positive-ion mode and the source voltage was between 4 and 6 kV. The sample was introduced in acetonitrile with a syringe pump at a flow rate of 18 ml/hr. The drying gas (N_2) was held at 100°C and the spray gas was held at 110°C. The machine was calibrated prior to every experiment via direct infusion of a TFA-Na solution, which provided a m/z range of singly charged peaks up to 3500 Da. Software acquisition Compass 2.0 for Otof series. Software processing m-mass.

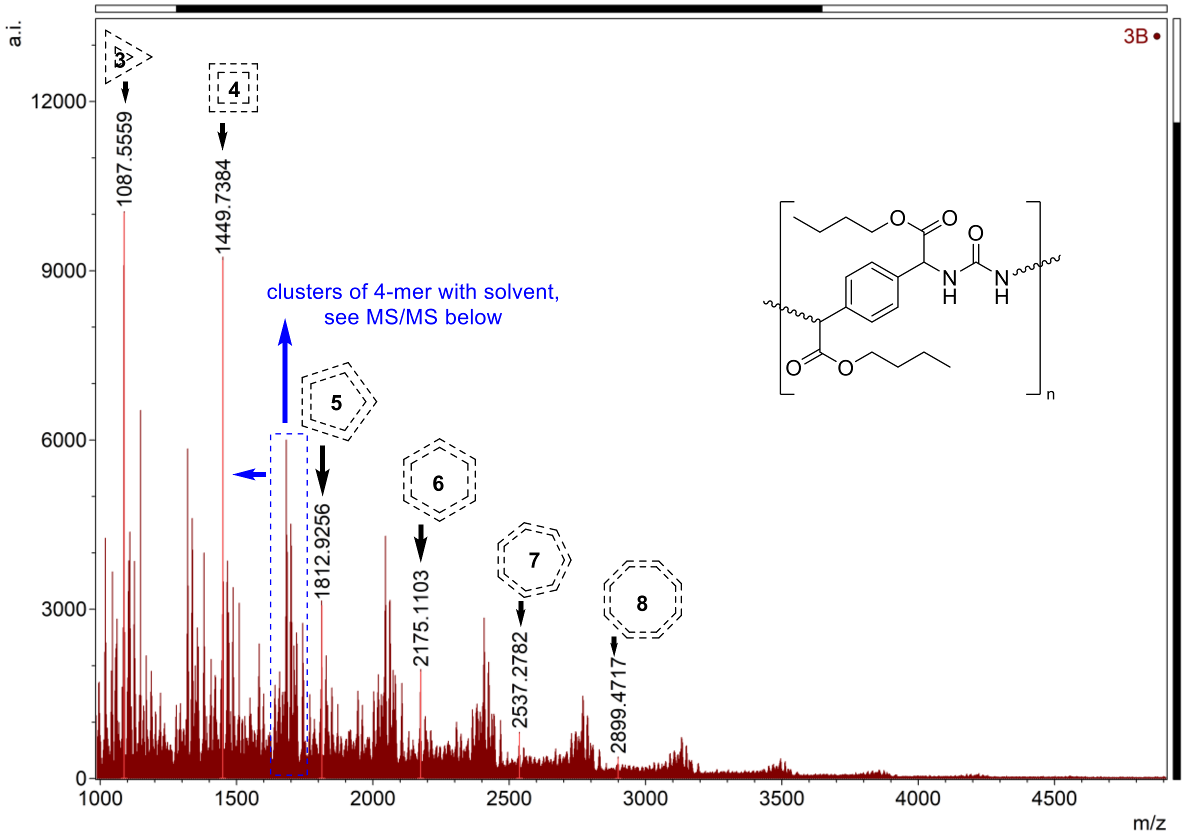

Figure S24. ESI-MS spectrum of polymer 3b.

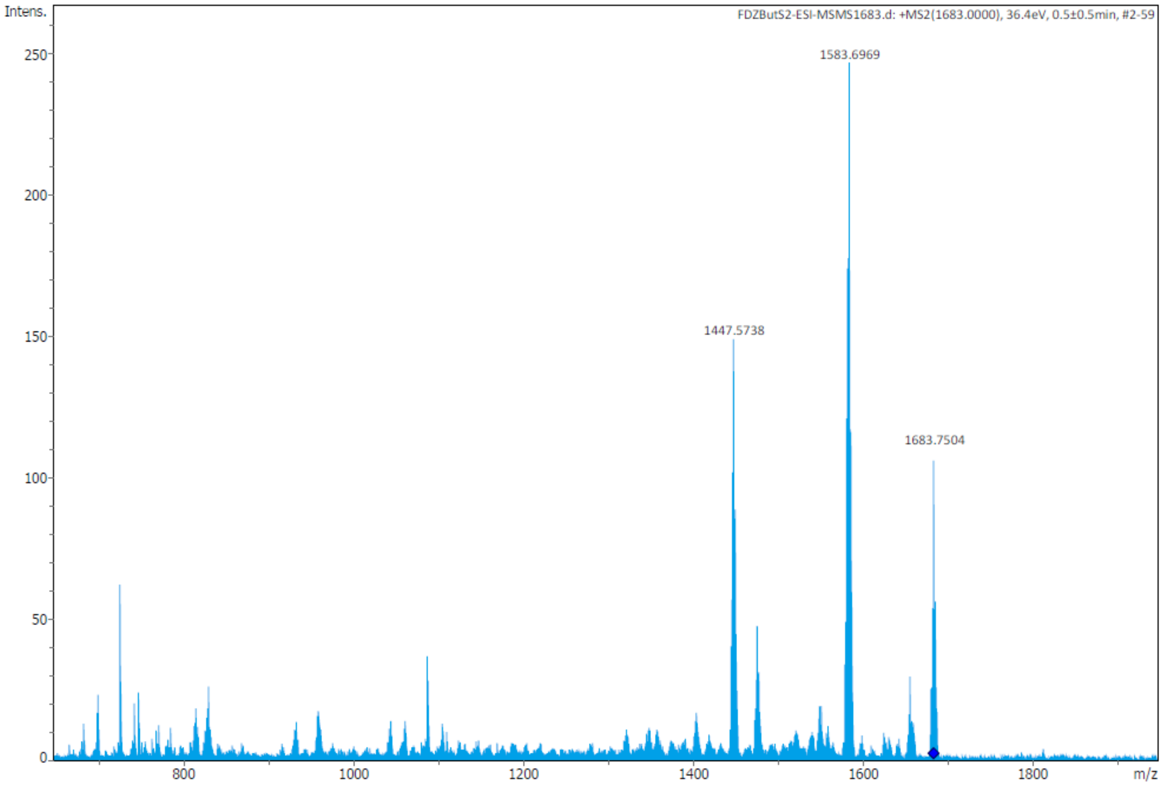

Figure S25. MS-MS analysis of solvent-polymer cluster at m/z = 1683.7504.

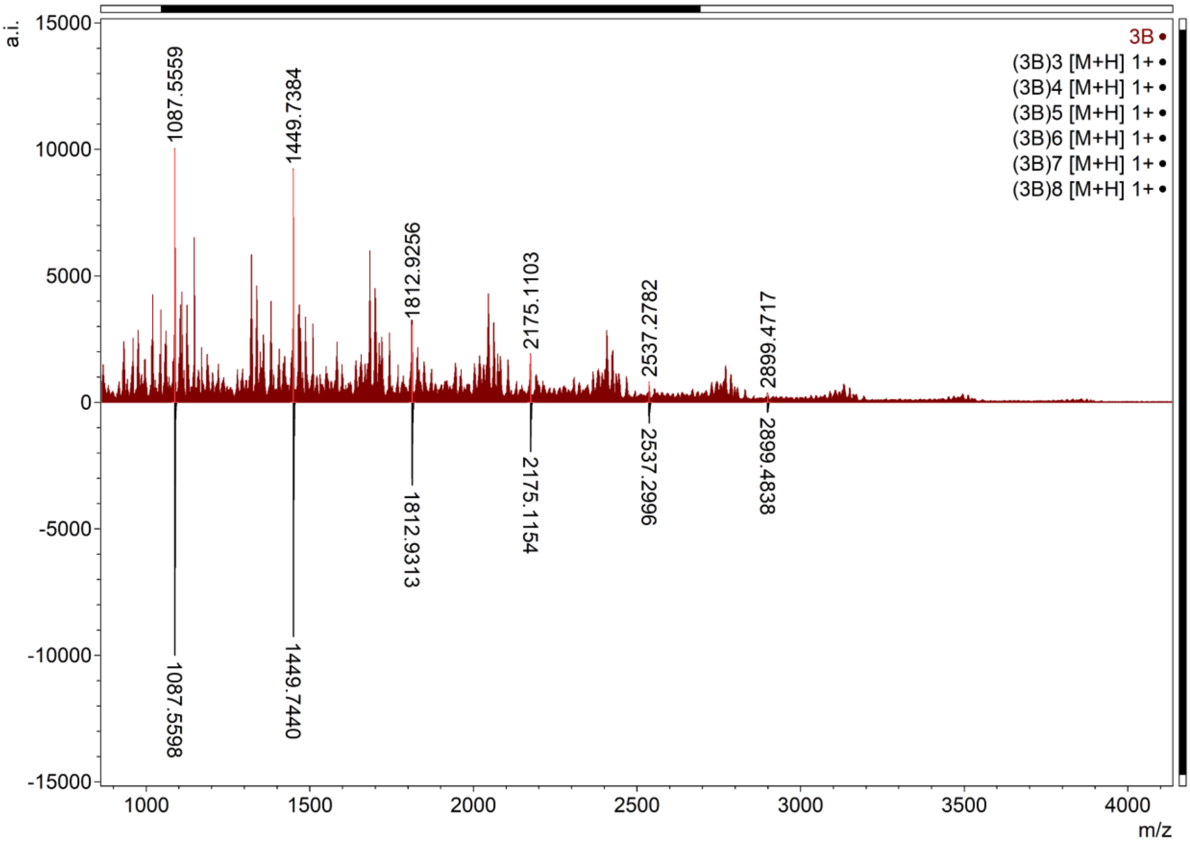

Figure S26. ESI-MS spectrum of polymer 3b (up) and calculated (down) m/z for n*($C_{19}H_{26}N_2O_5$).

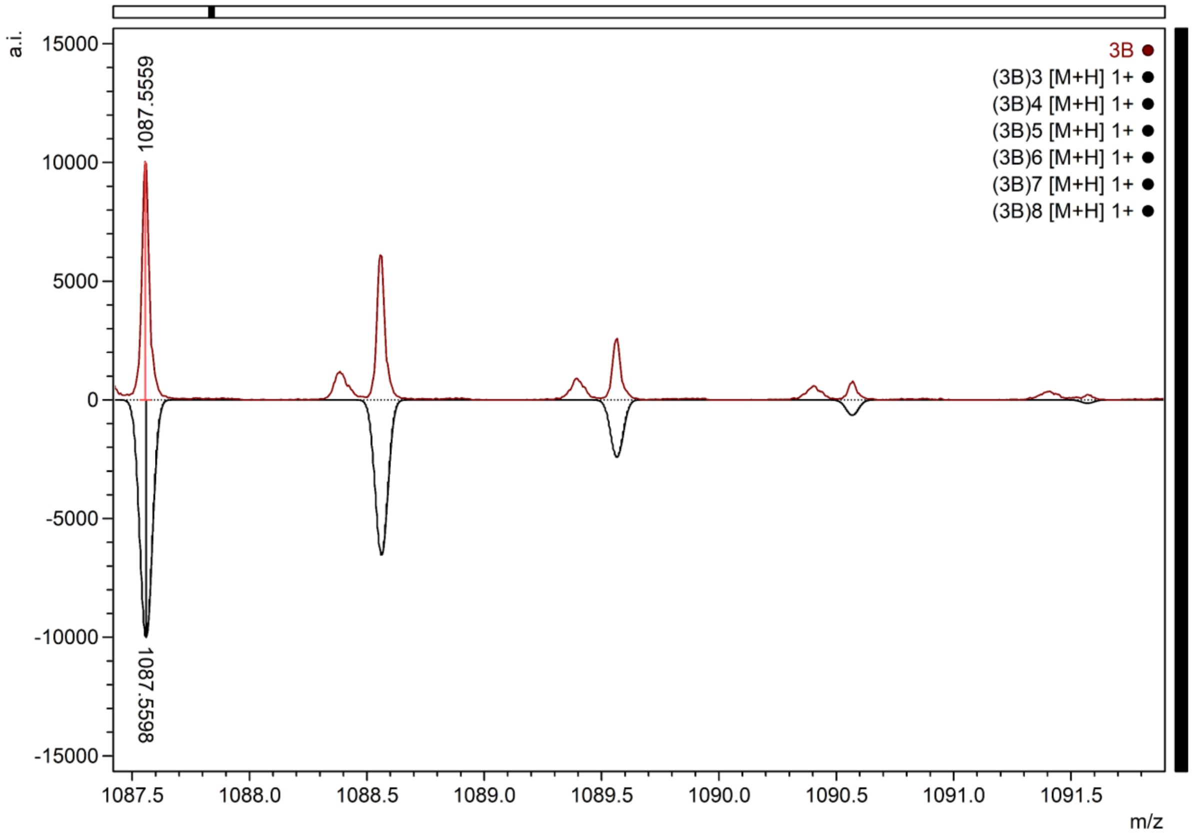

Figure S27. ESI-MS spectrum of polymer 3b (up) and calculated (down) m/z for 3*($C_{19}H_{26}N_2O_5$).

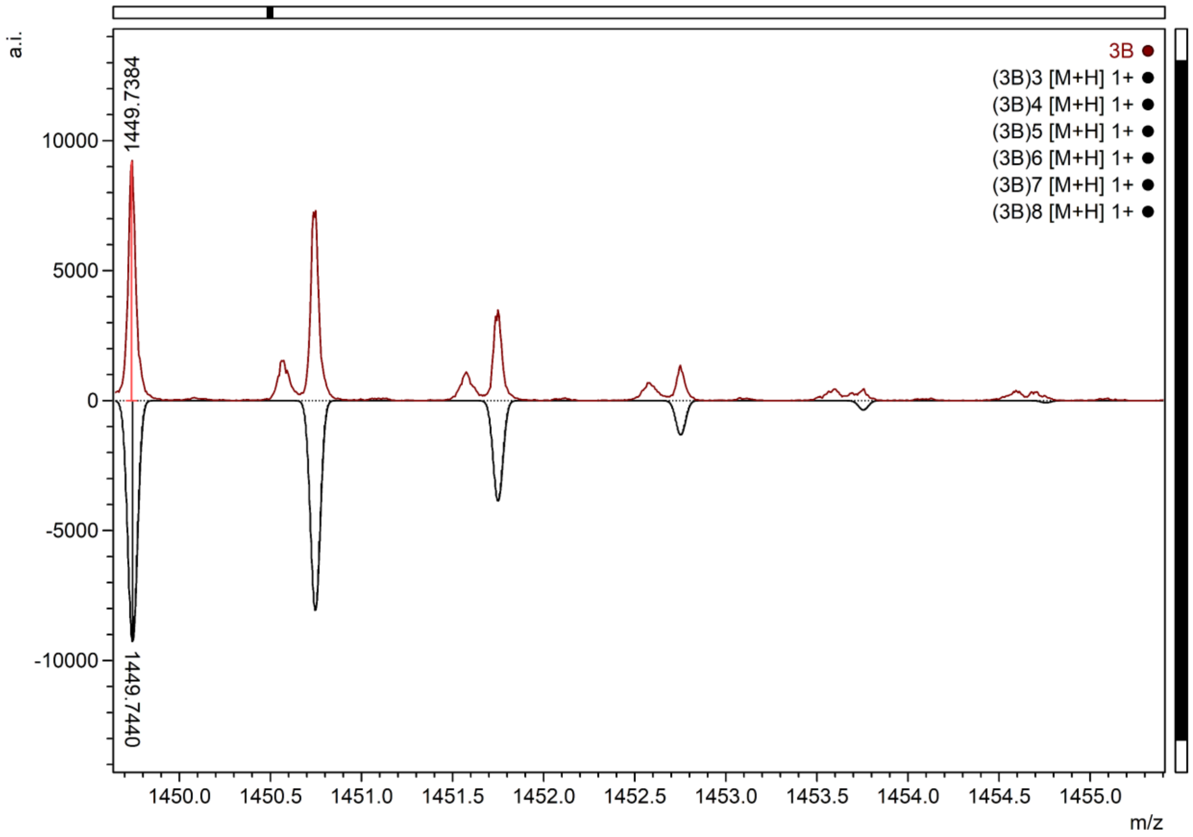

Figure S28. ESI-MS spectrum of polymer 3b (up) and calculated (down) m/z for 4*($C_{19}H_{26}N_2O_5$).

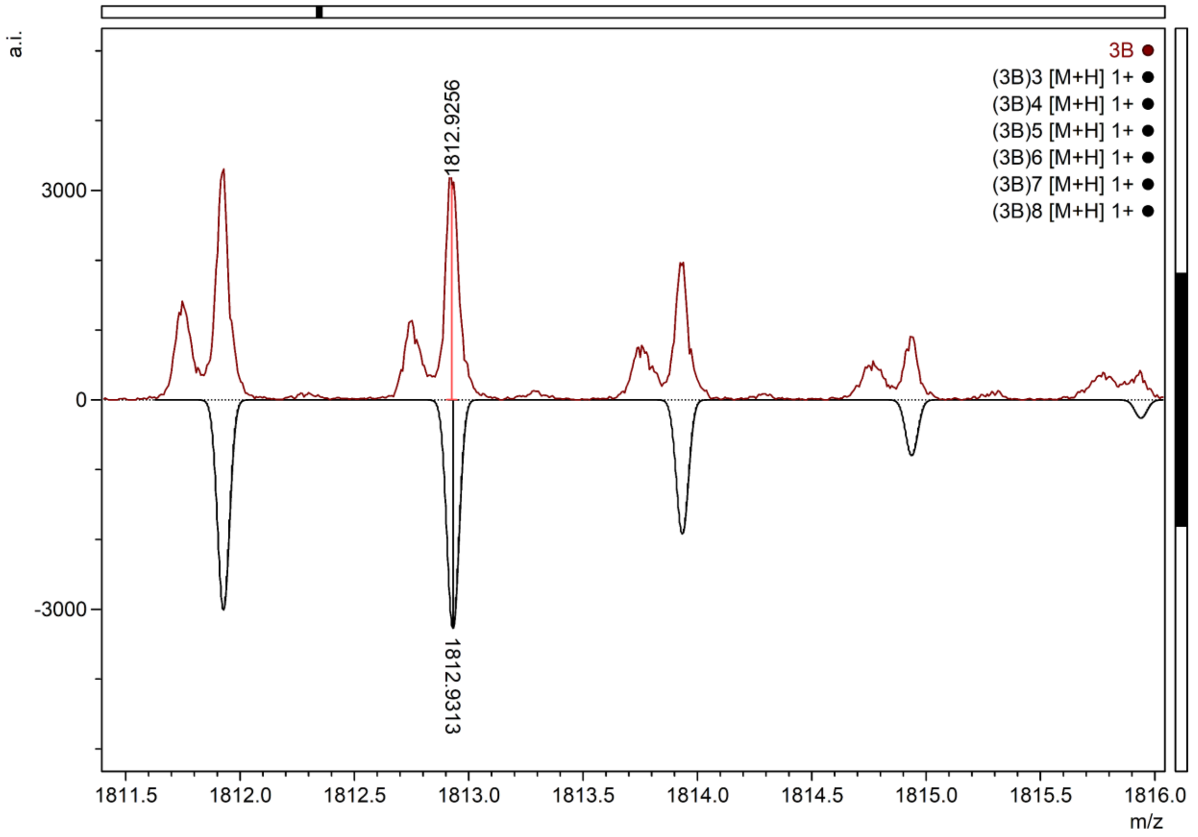

Figure S29. ESI-MS spectrum of polymer 3b (up) and calculated (down) m/z for 5*($C_{19}H_{26}N_2O_5$).

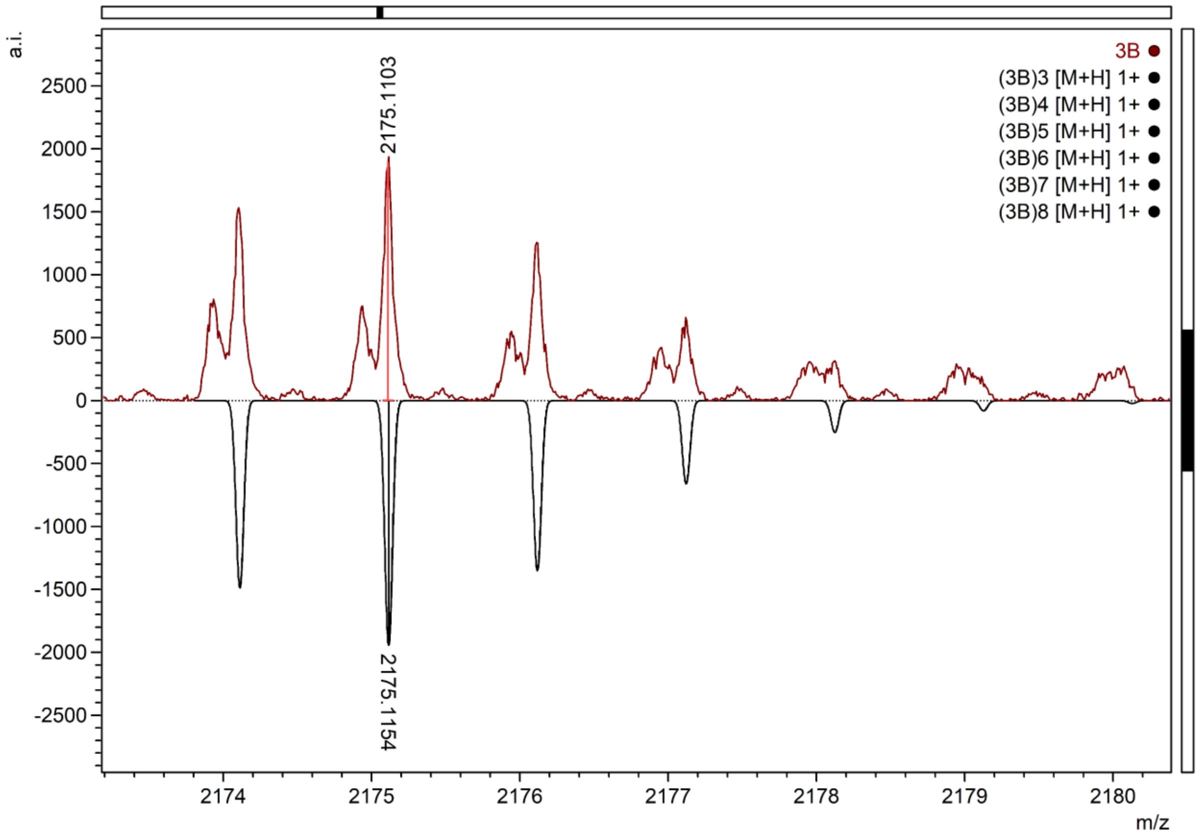

Figure S30. ESI-MS spectrum of polymer 3b (up) and calculated (down) m/z for 6*($C_{19}H_{26}N_2O_5$).

MALDI MS

Matrix-assisted laser desorption/ionization MS. Stock solutions of polymers **3a**, **3b** and **3e** were prepared in acetonitrile and diluted to 1 µg/mL for analysis. 1 µL of this dilution was spotted in duplicate onto a polished steel MALDI target plate, and mixed with 1 µL of matrix solution [10mg/ml α-Cyano-4-hydroxycinnamic acid (HCCA) in 70% acetonitrile / 30% of 0.1% trifluoracetic acid (TFA)] directly on the spot. Matrix Assisted Laser Desorption/Ionization (MALDI) time-of-flight (TOF) MS was performed on a Bruker UltrafleXtreme MALDI TOF-TOF instrument (Bruker Daltonics, Bremen, Germany) using ImageFlex (version 3.4, Bruker 6 Daltonics). Reflector positive MALDI-TOF spectra were recorded between m/z 1000 and 20000 under the following conditions: 320 ns delayed extraction; signal deflection up to m/1000; 2 kHz Smartbeam-II UV laser (Nd:YAG; λ = 355 nm) operating with the "4_large" parameter set; 1 GS/s digitizer sampling rate; ion source 1, 2, and lens voltages of 25.00, 2.60, and 9.00 kV, respectively, and reflector 1 and 2 26.46 and 13.45V.

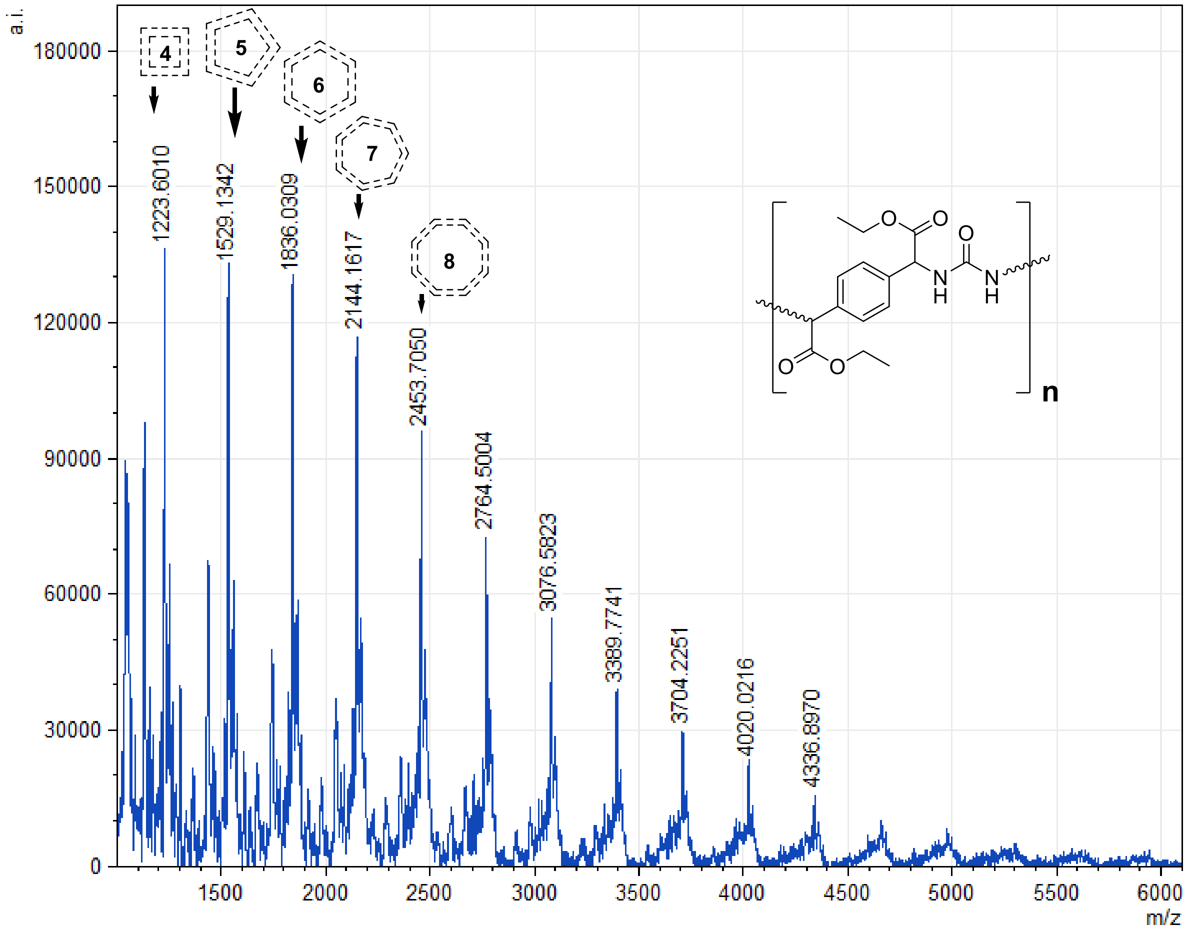

Figure S31. MALDI-TOF Mass spectrum of polymer 3a, arrows indicating m/z = n*($C_{15}H_{18}N_2O_5$).

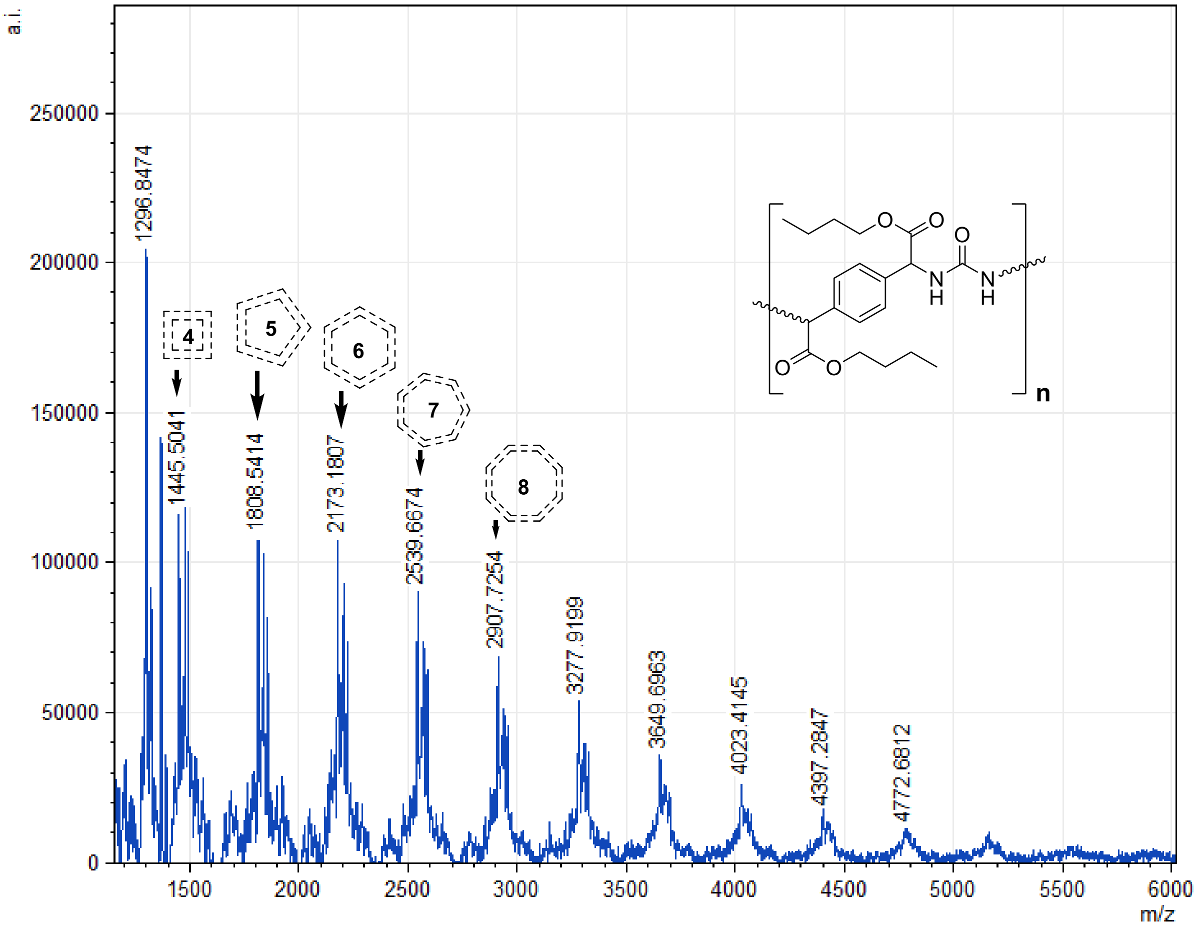

Figure S32. MALDI-TOF Mass spectrum of polymer 3b, arrows indicating m/z = n*($C_{19}H_{26}N_2O_5$).

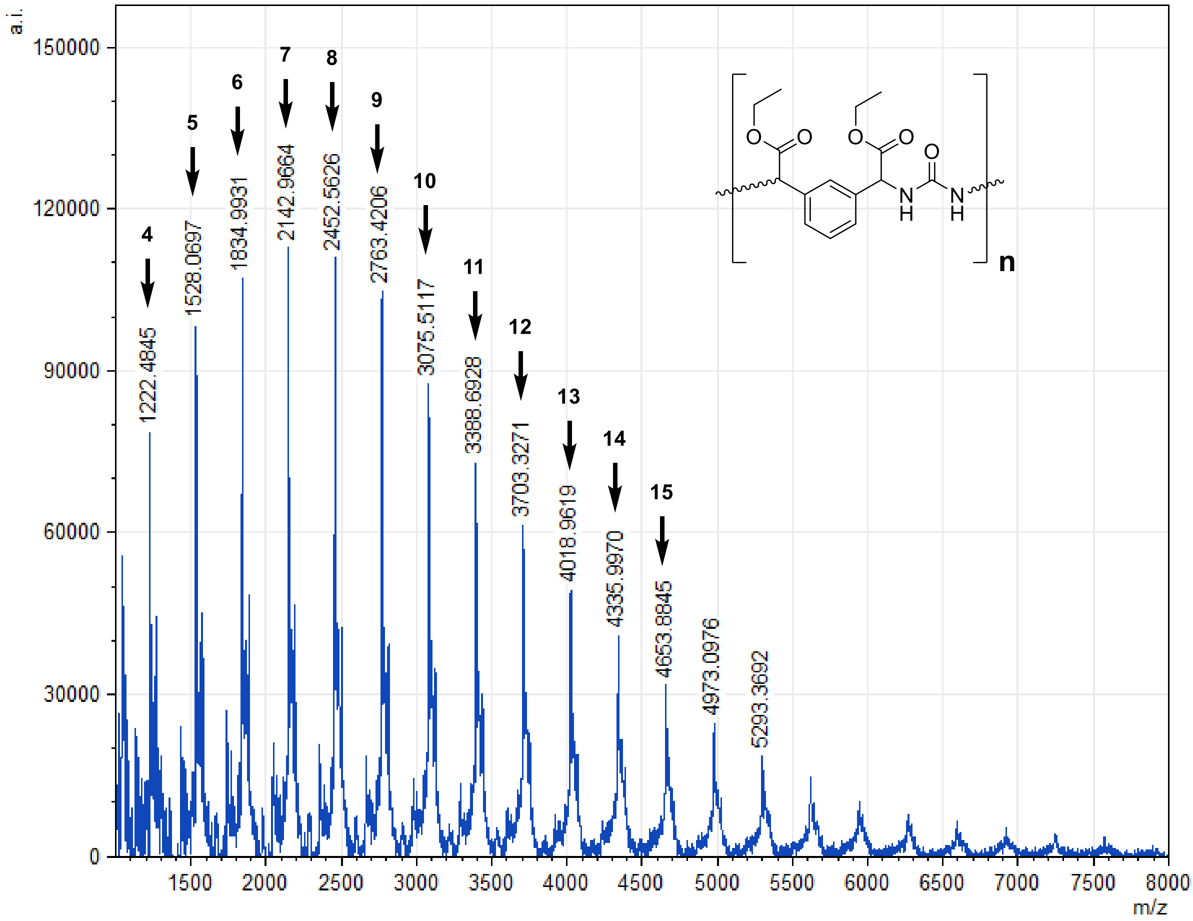

Figure S33. MALDI-TOF Mass spectrum of polymer 3e, arrows indicating m/z = n*($C_{15}H_{18}N_2O_5$).

NMR Spectra

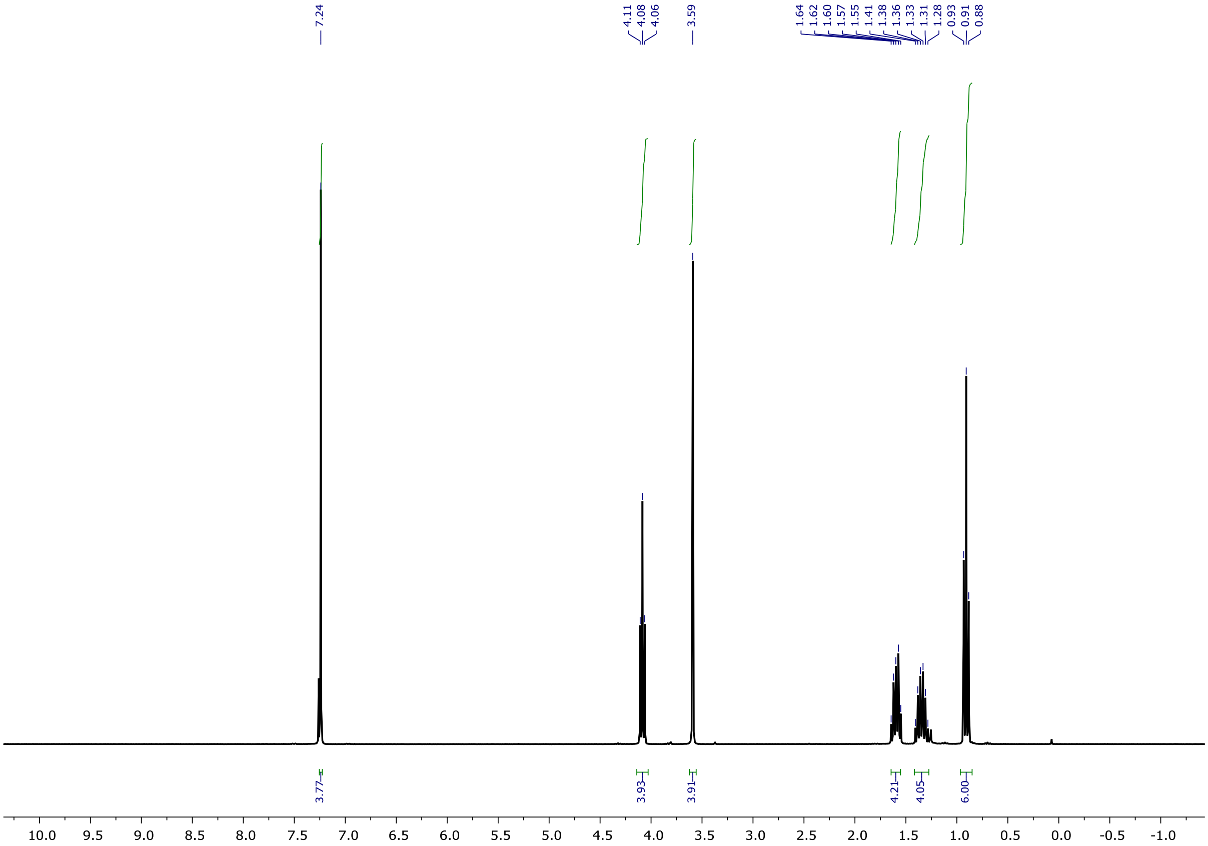

Figure S34. ^{1}H-NMR spectrum of ester 1b.

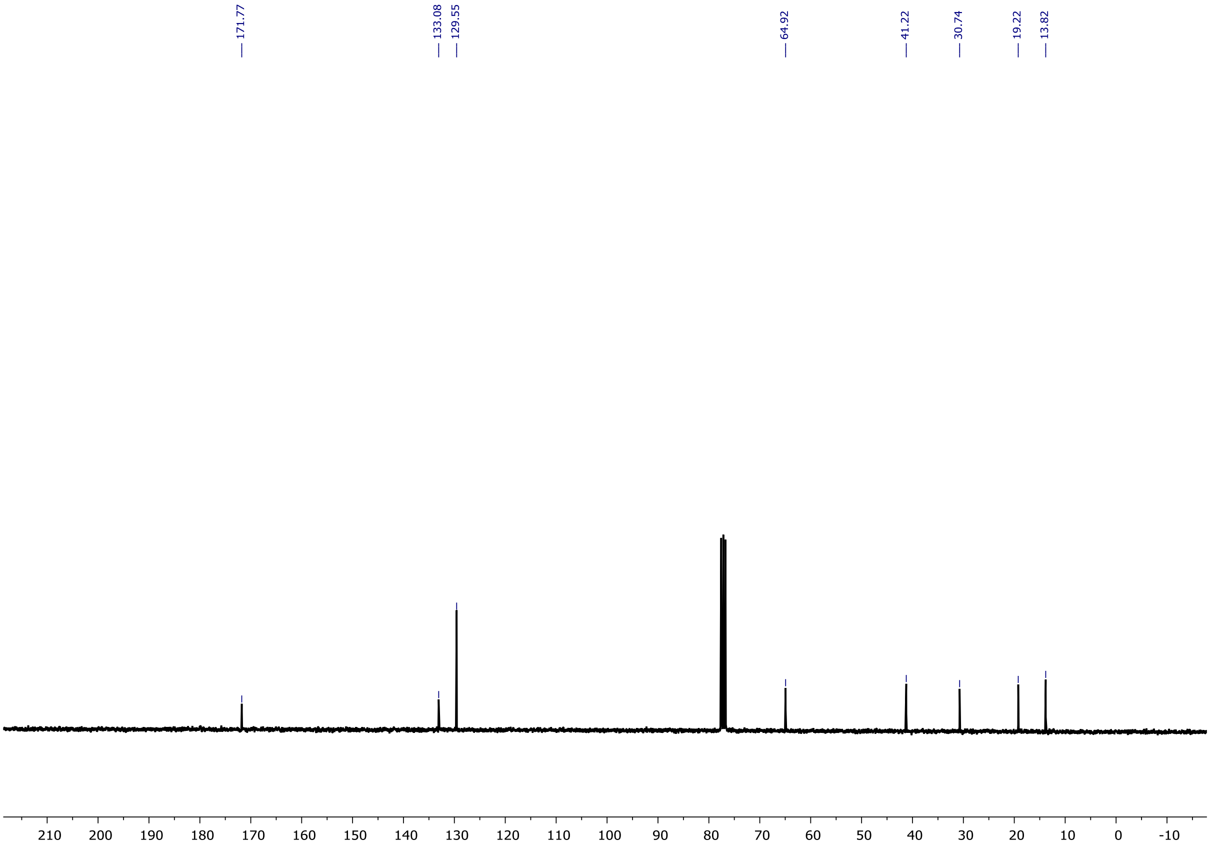

Figure S35. ^{13}C-NMR spectrum of ester 1b.

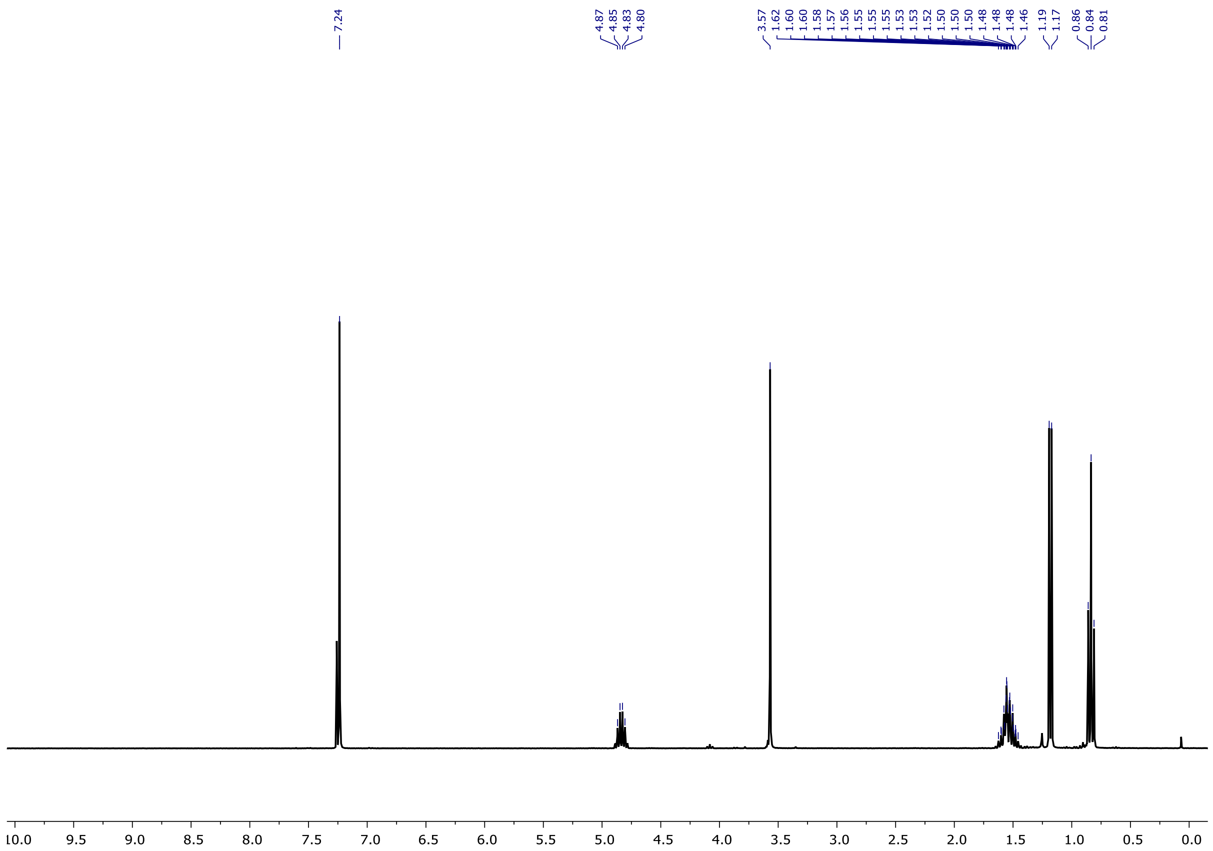

Figure S36. ^{1}H-NMR spectrum of ester 1c.

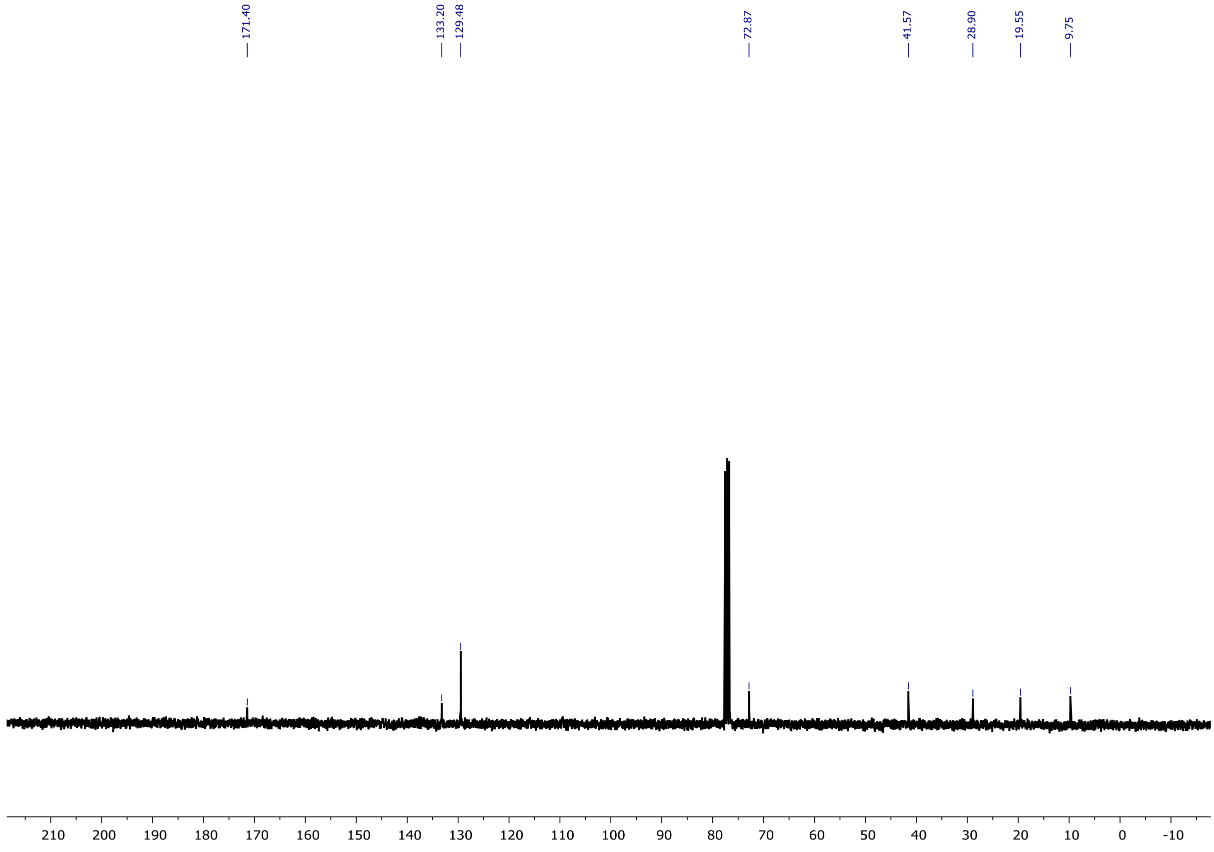

Figure S37. ^{13}C-NMR spectrum of ester 1c.

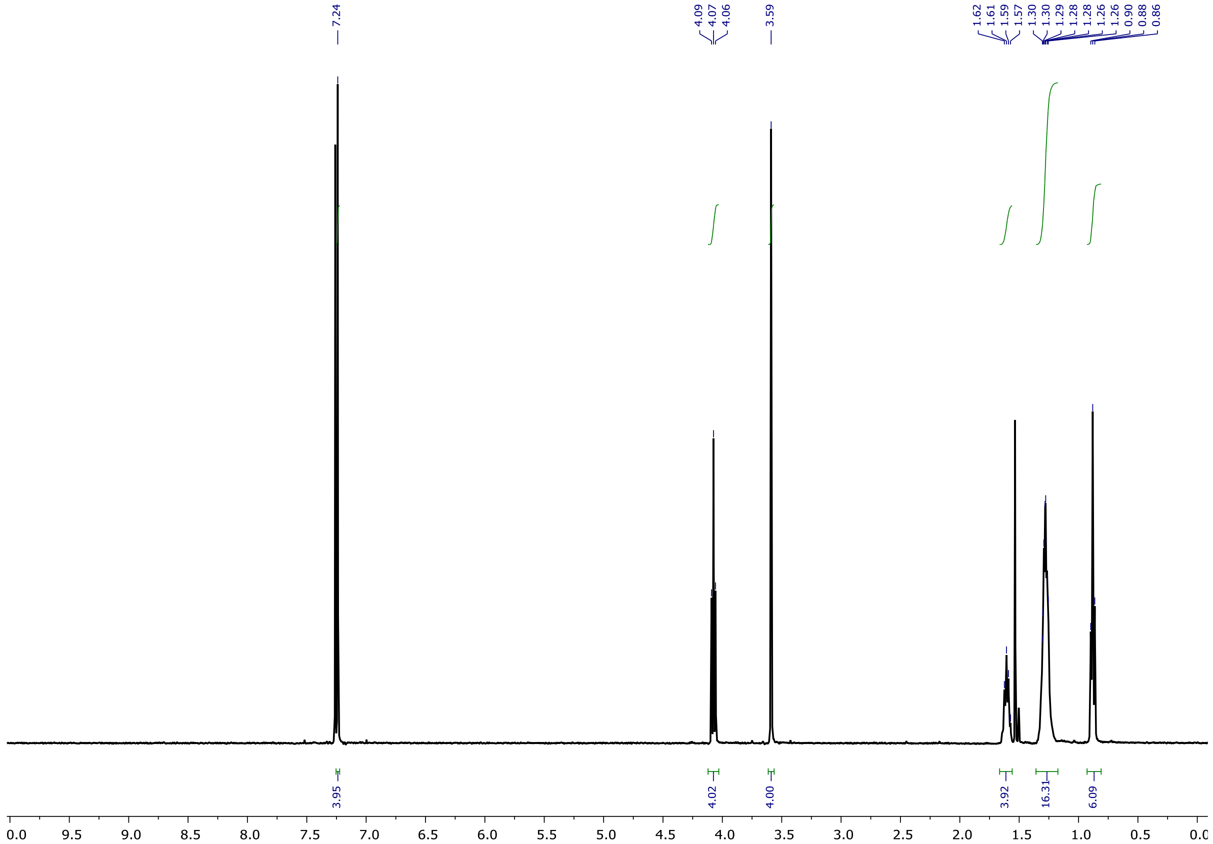

Figure S38. ¹H-NMR spectrum of ester 1d.

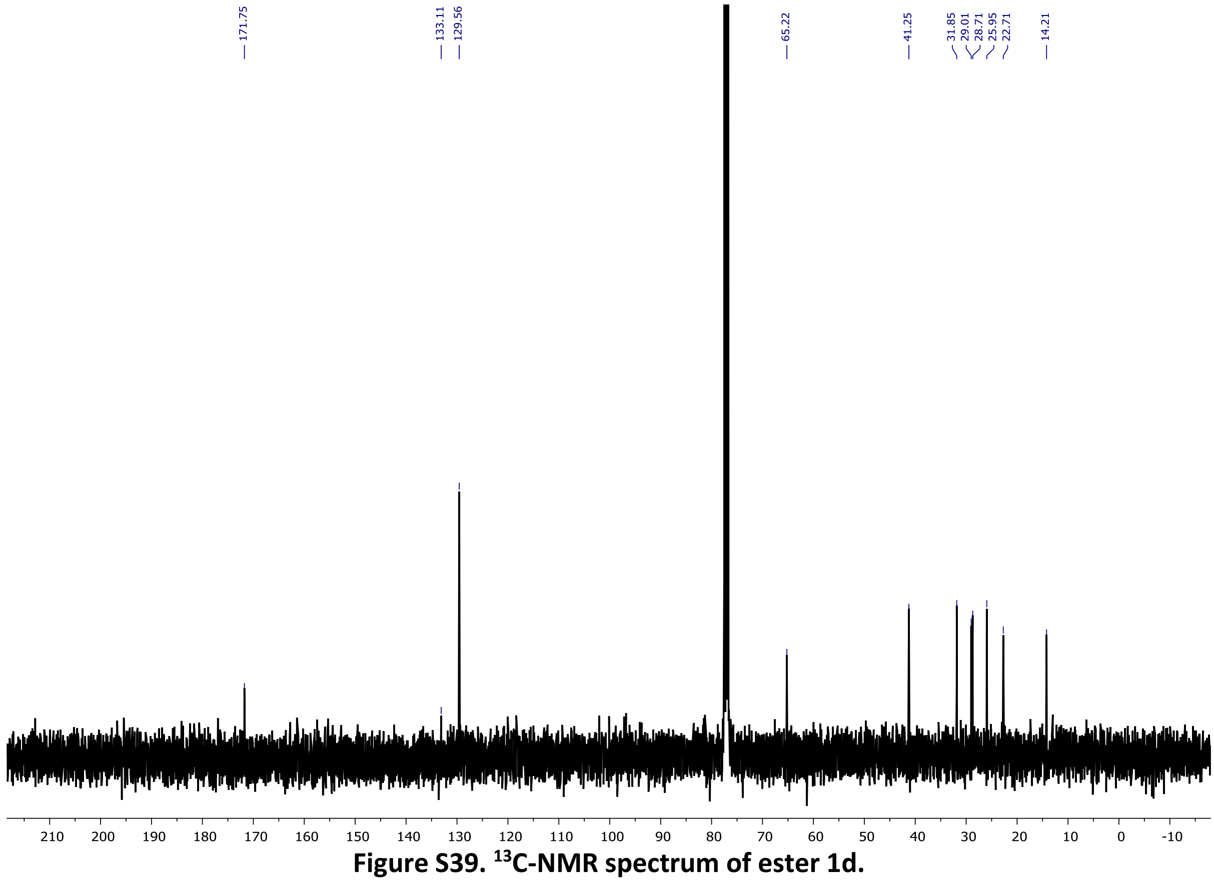

Figure S39. ¹³C-NMR spectrum of ester 1d.

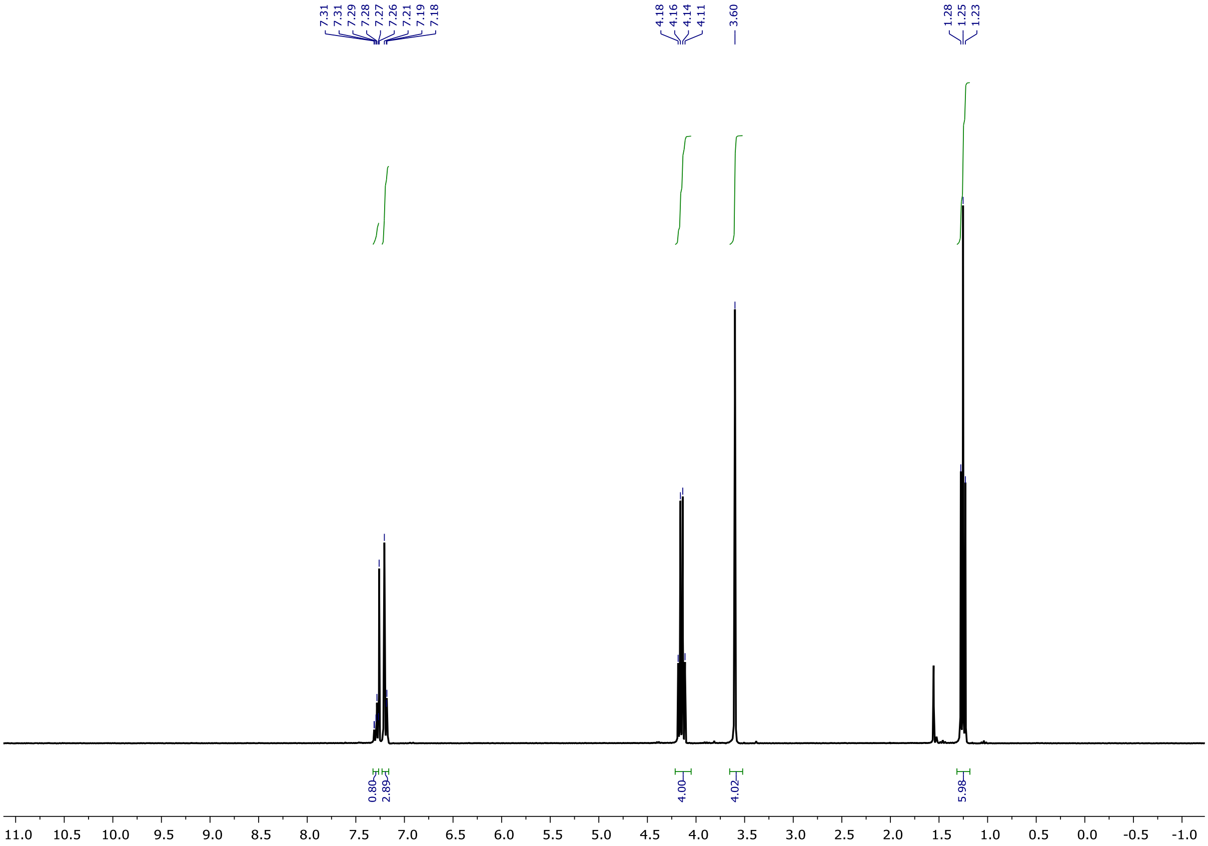

Figure S40. ^{1}H-NMR spectrum of ester 1e.

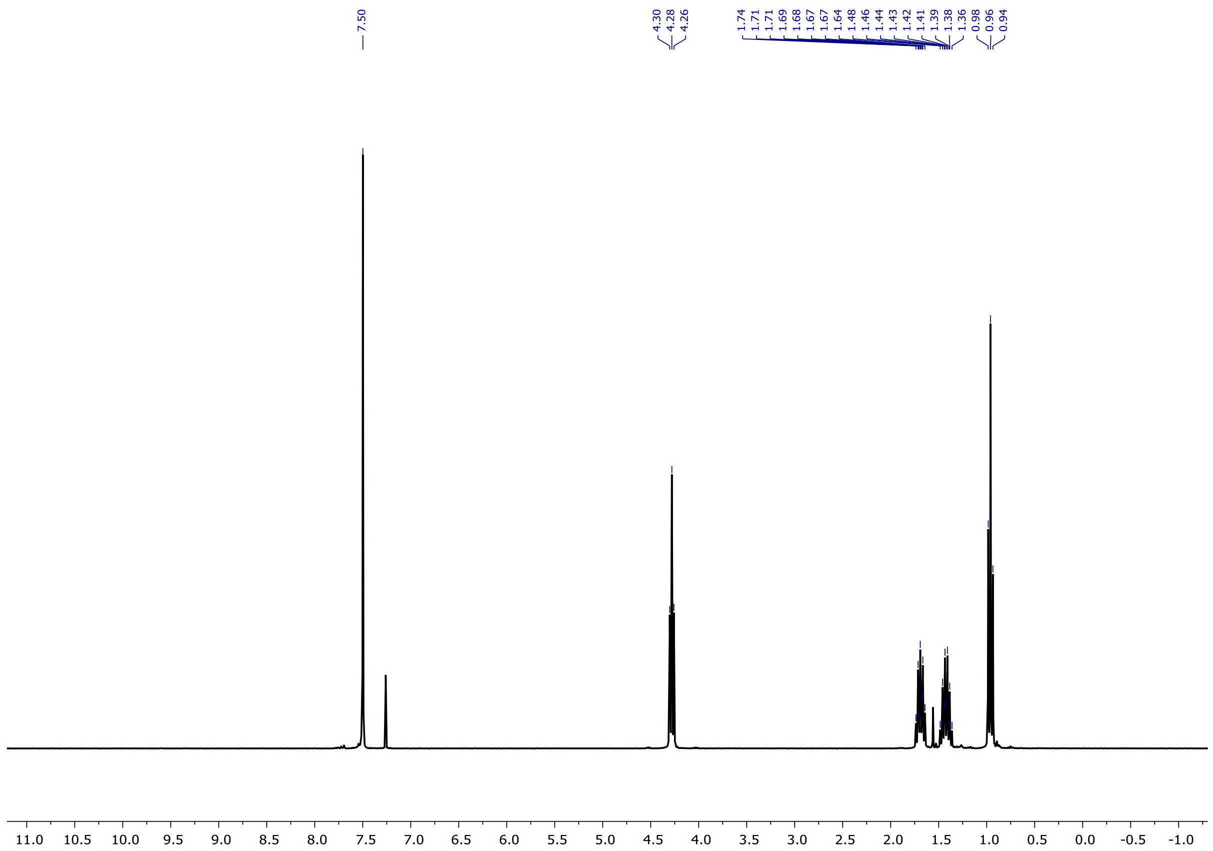

Figure S41. ^{1}**H-NMR spectrum of diazo 2b.**

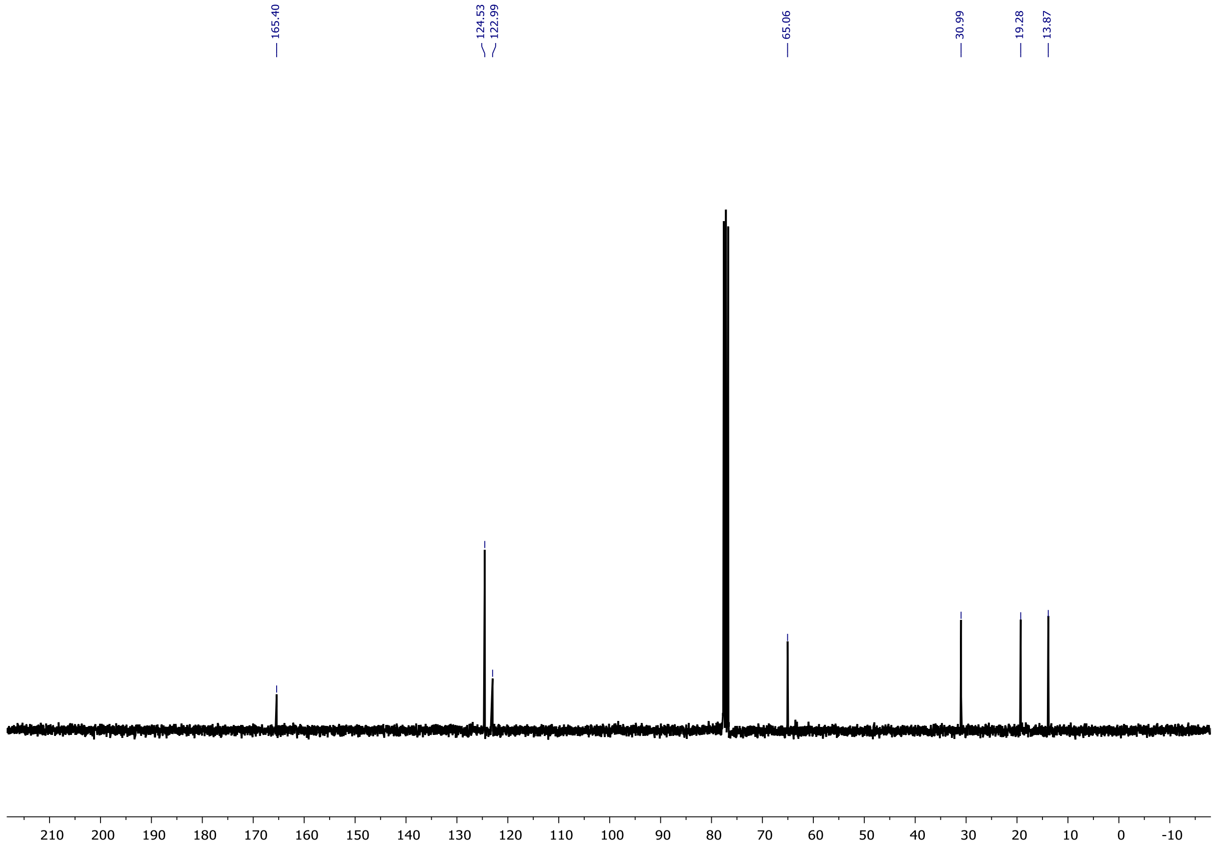

Figure S42. ^{13}**C-NMR spectrum of diazo 2b.**

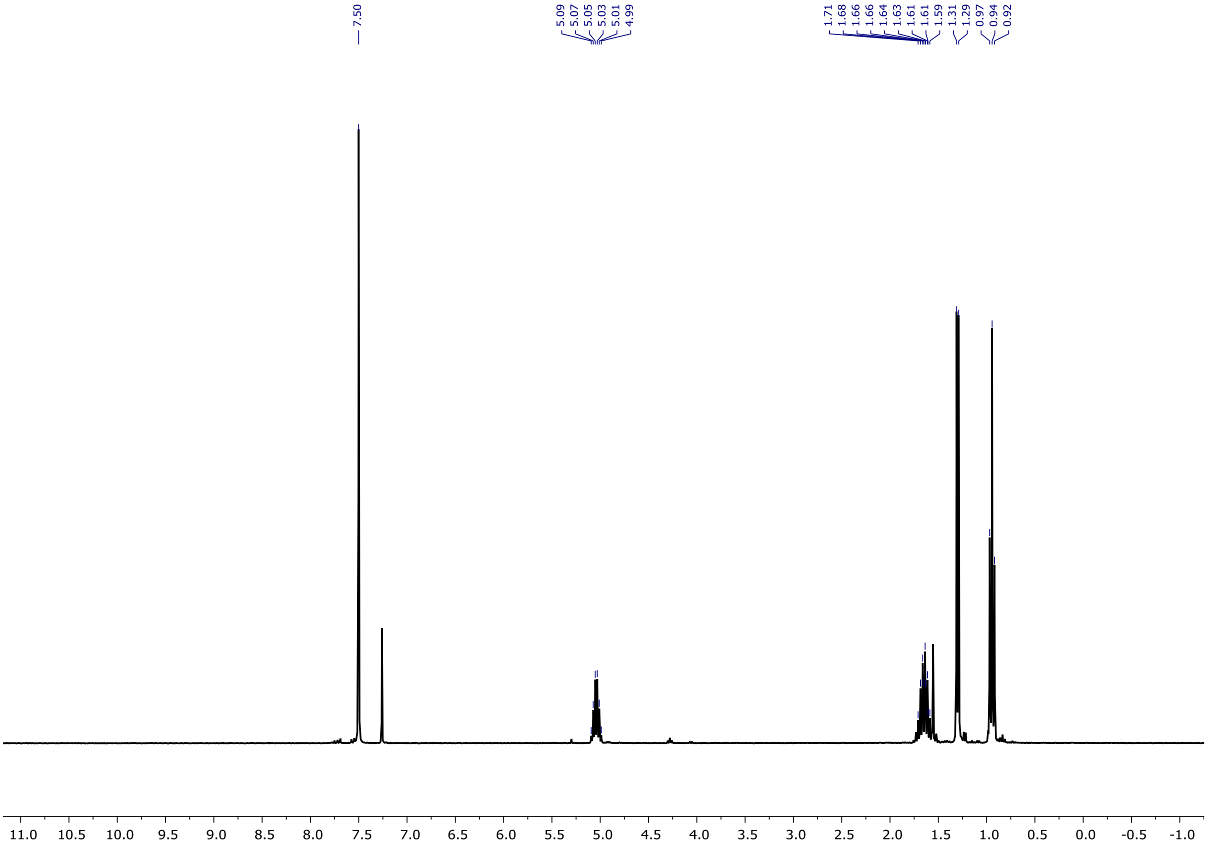

Figure S43. **¹H-NMR spectrum of diazo 2c.**

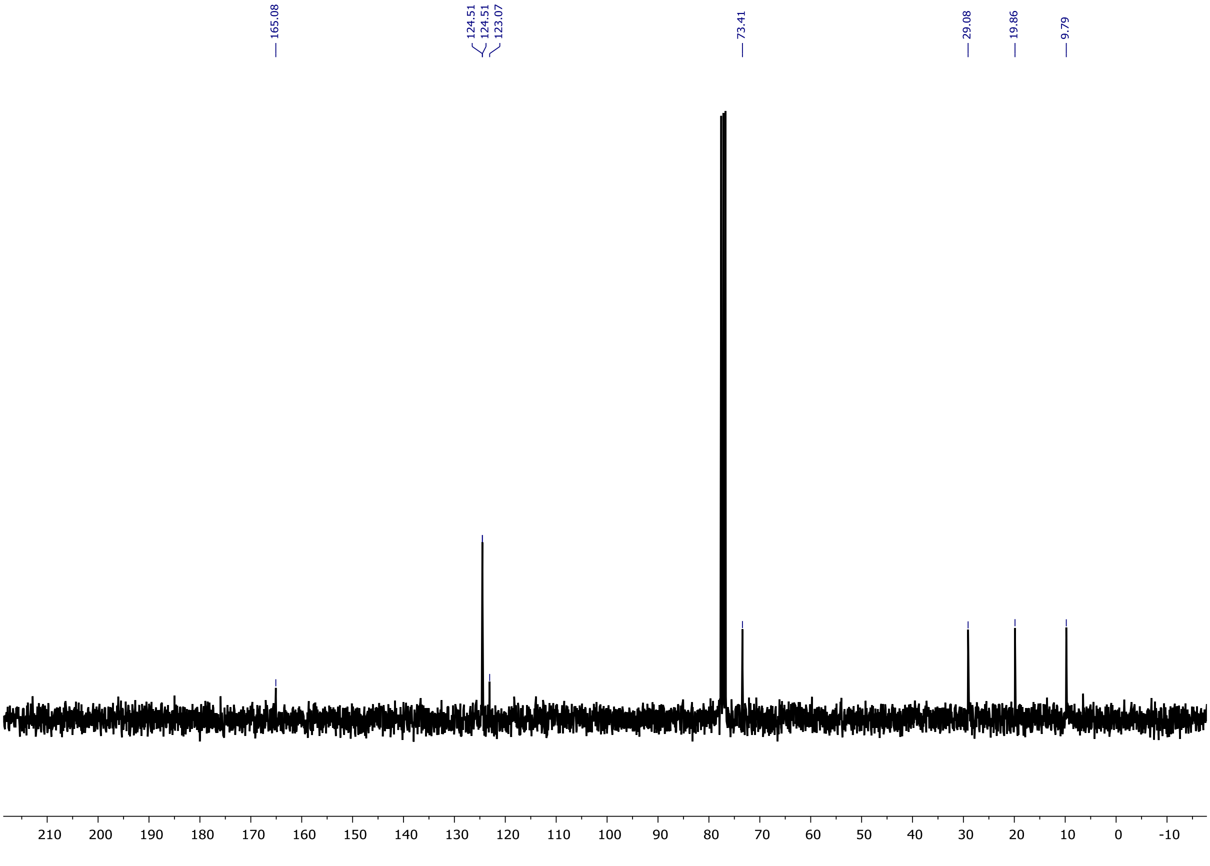

Figure S44. **¹³C-NMR spectrum of diazo 2c.**

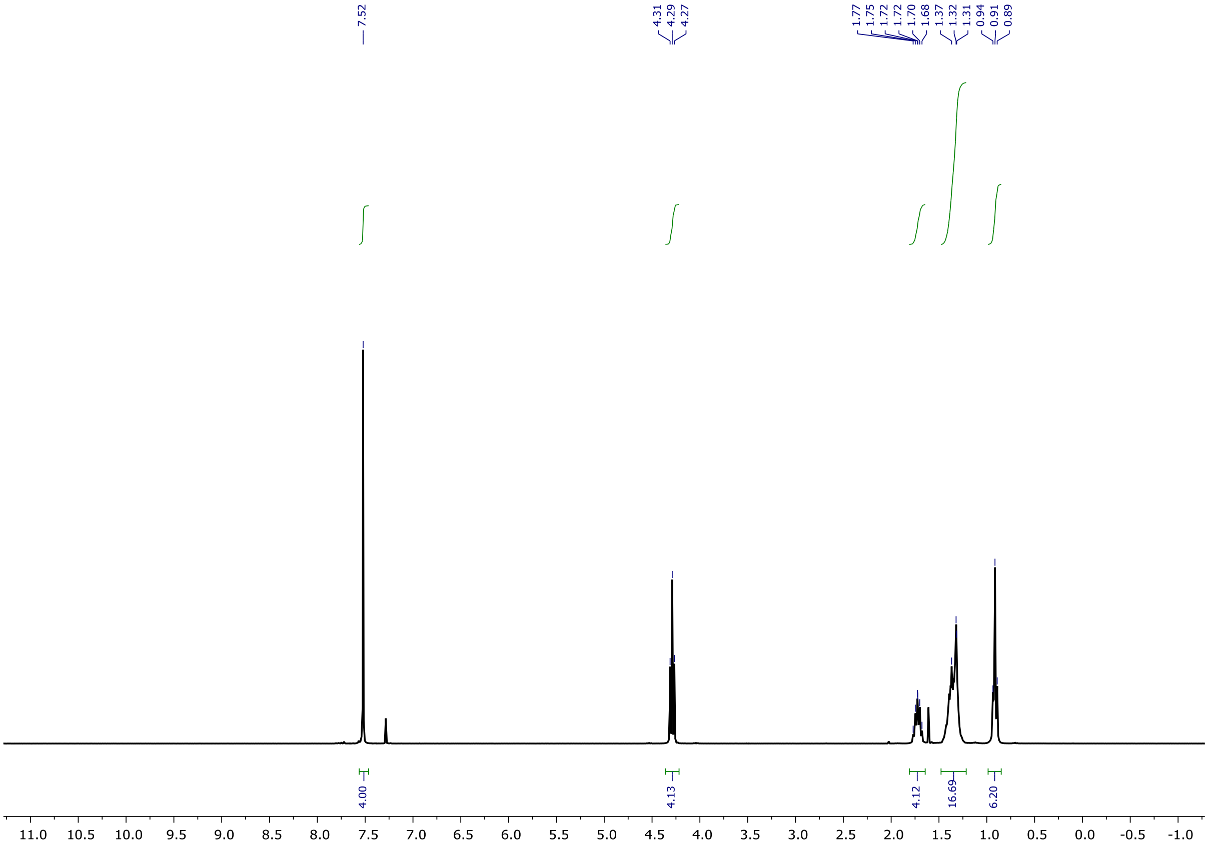

Figure S45. ^{1}H-NMR spectrum of diazo 2d.

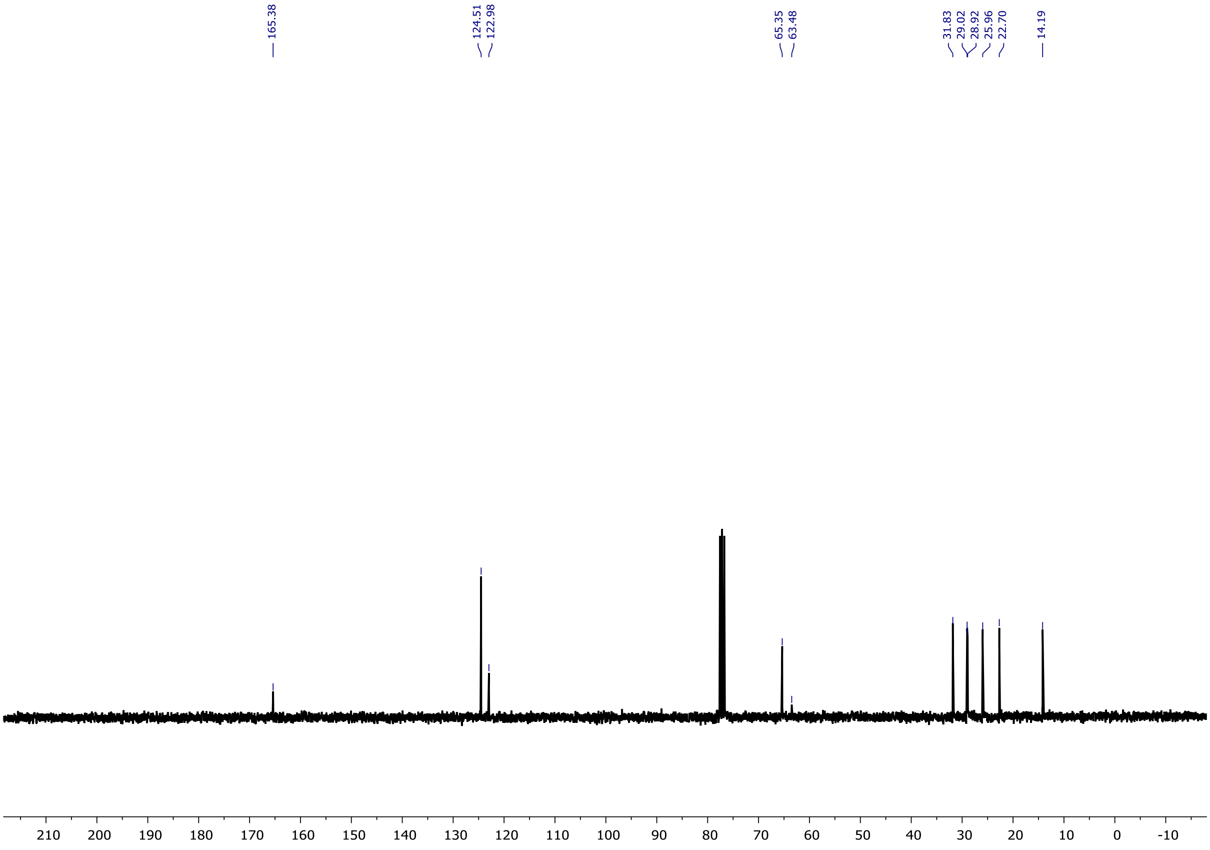

Figure S46. ^{13}C-NMR spectrum of diazo 2d.

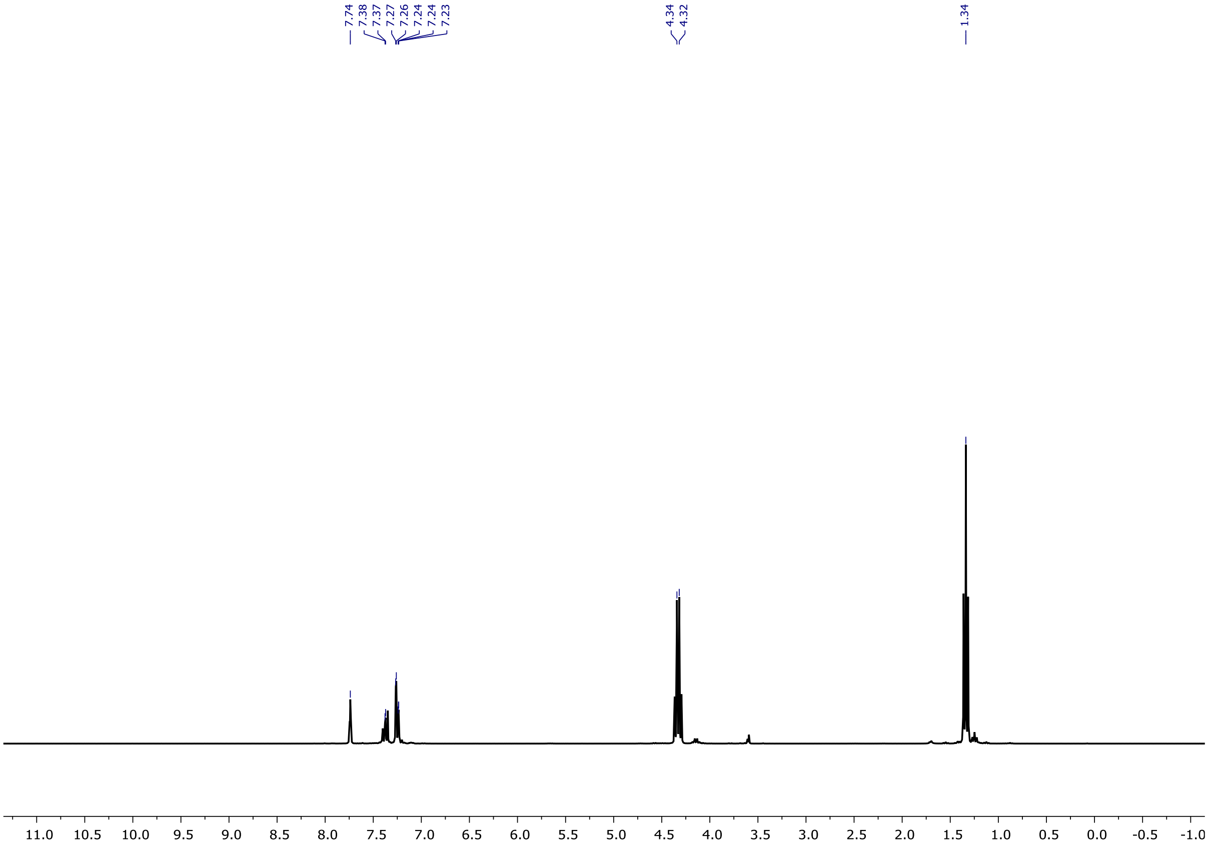

Figure S47. ^{1}H-NMR spectrum of diazo 2e.

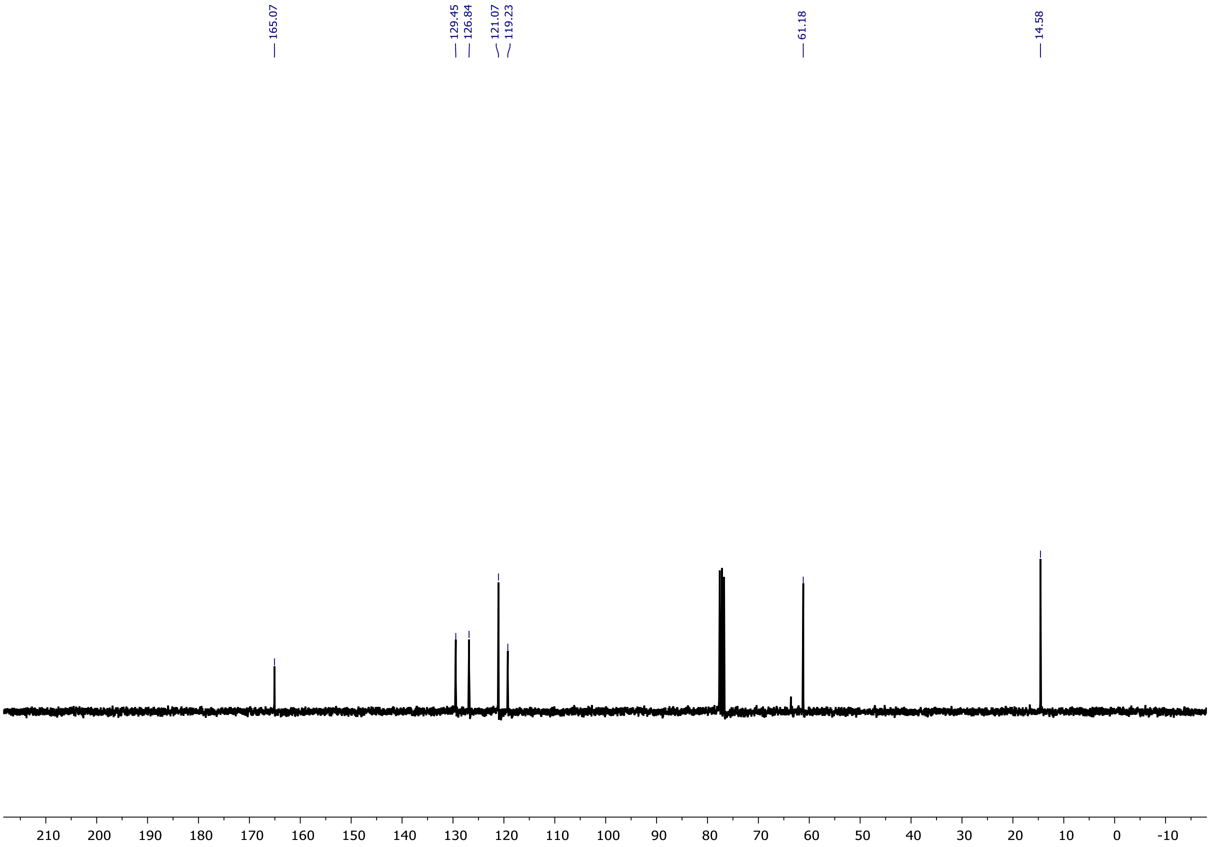

Figure S48. ^{13}C-NMR spectrum of diazo 2e.

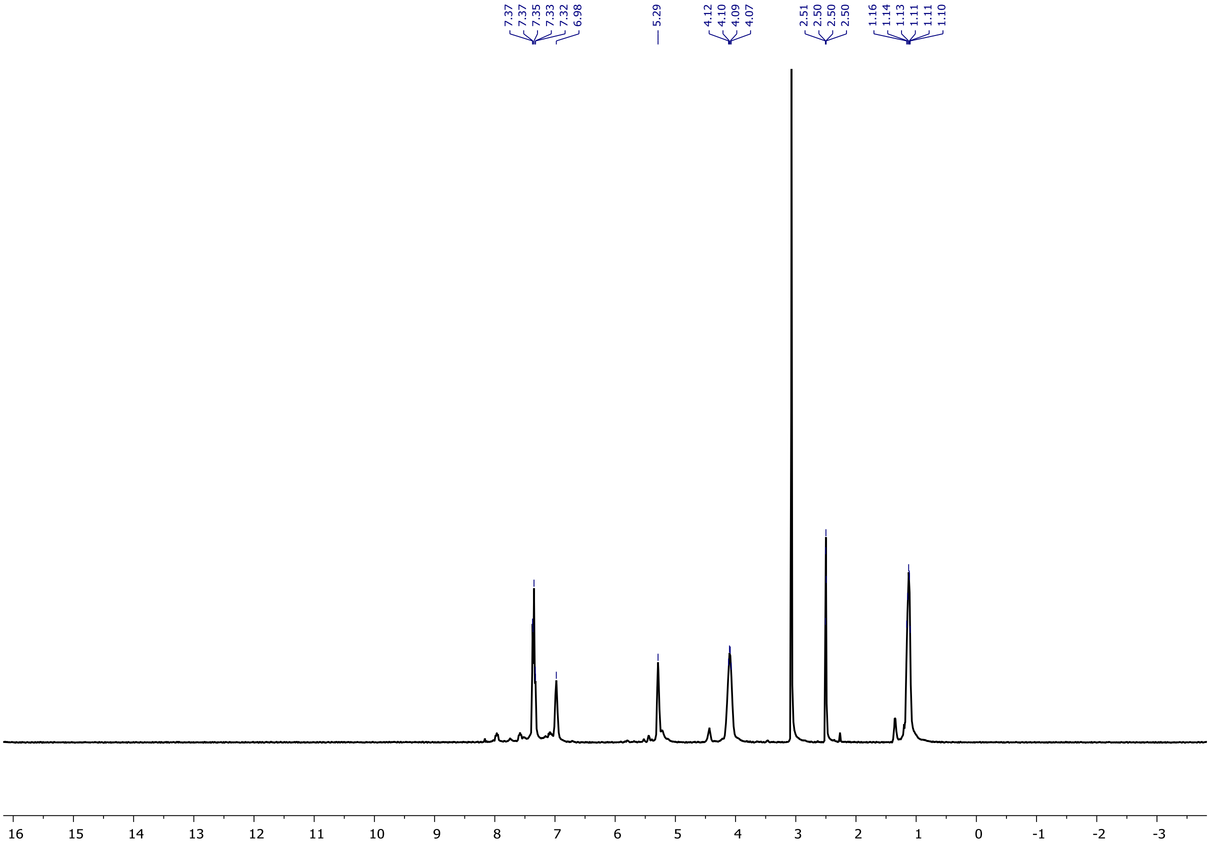

Figure S49. ^{1}H-NMR spectrum of polymer 3a.

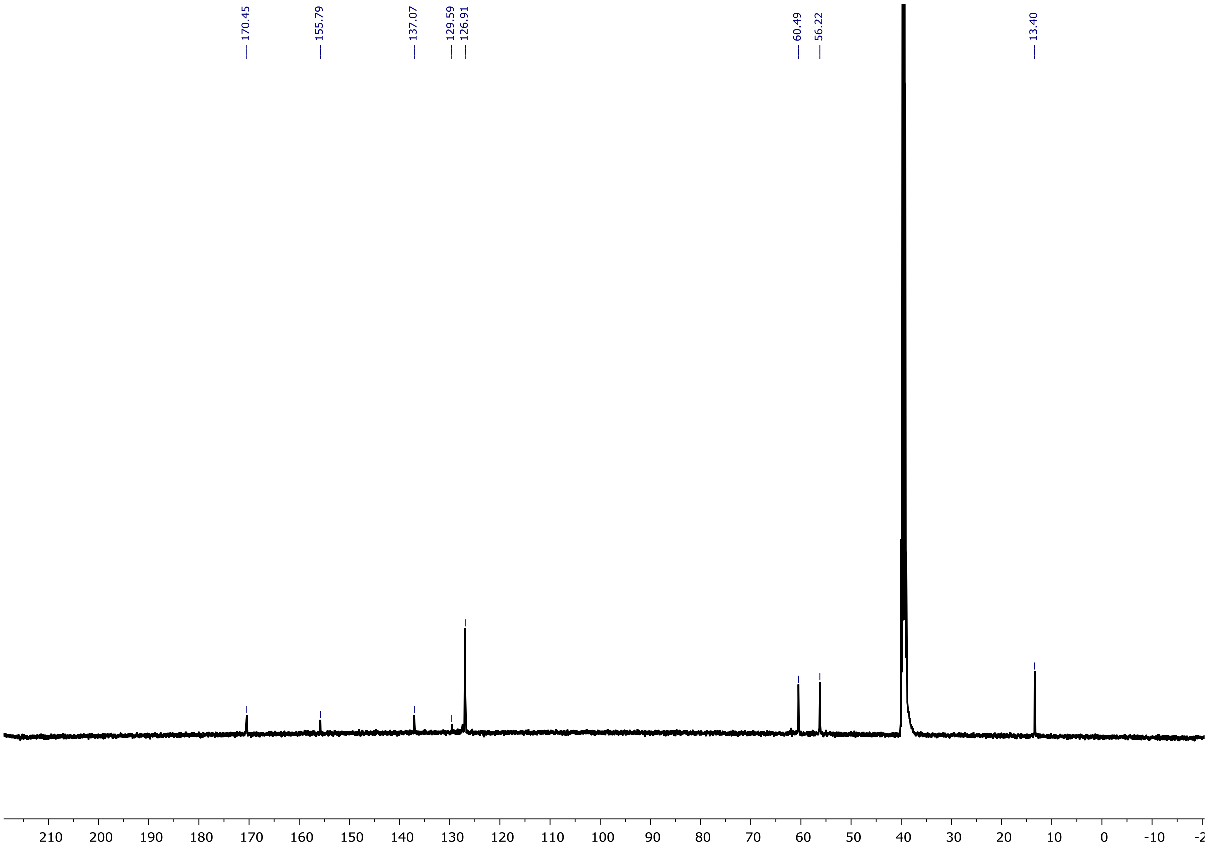

Figure S50. ^{13}C-NMR spectrum of polymer 3a.

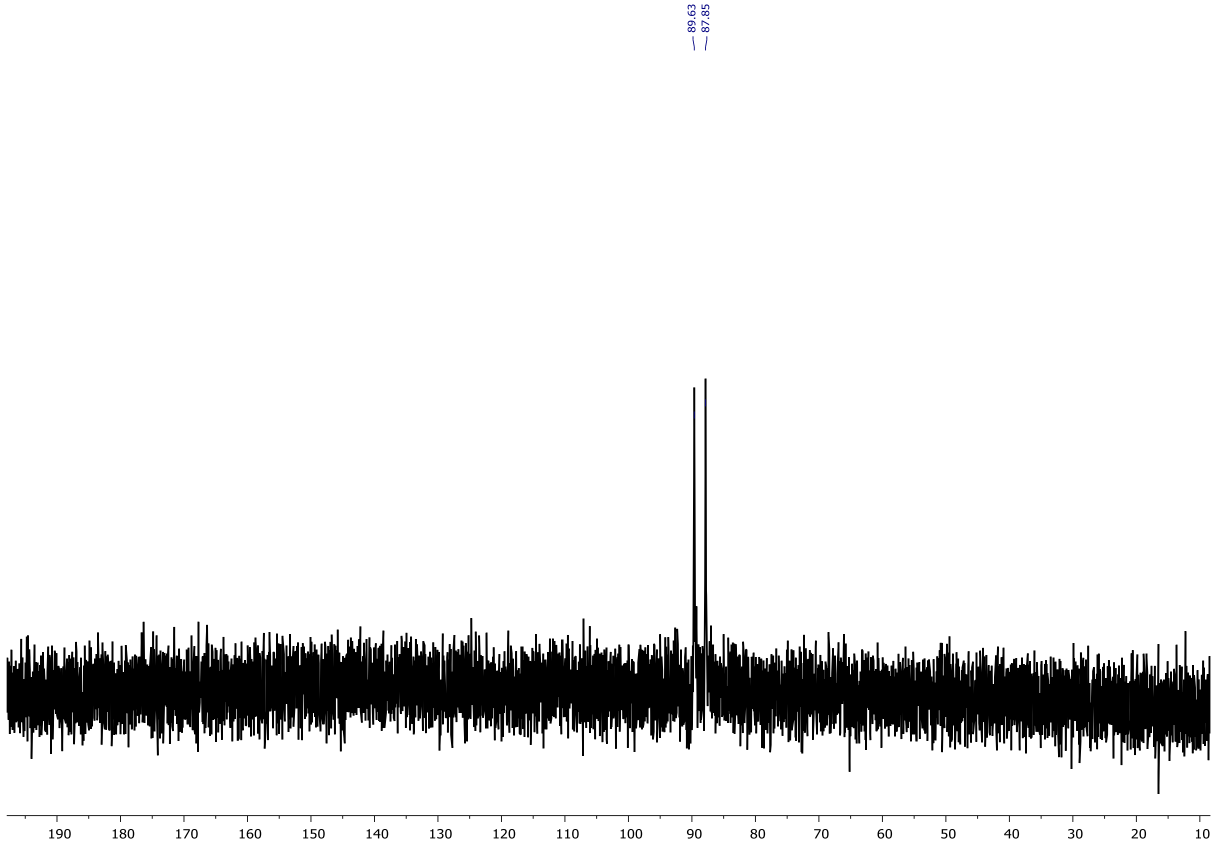

Figure S51. ^{15}**N-NMR spectrum of polymer 3b.**

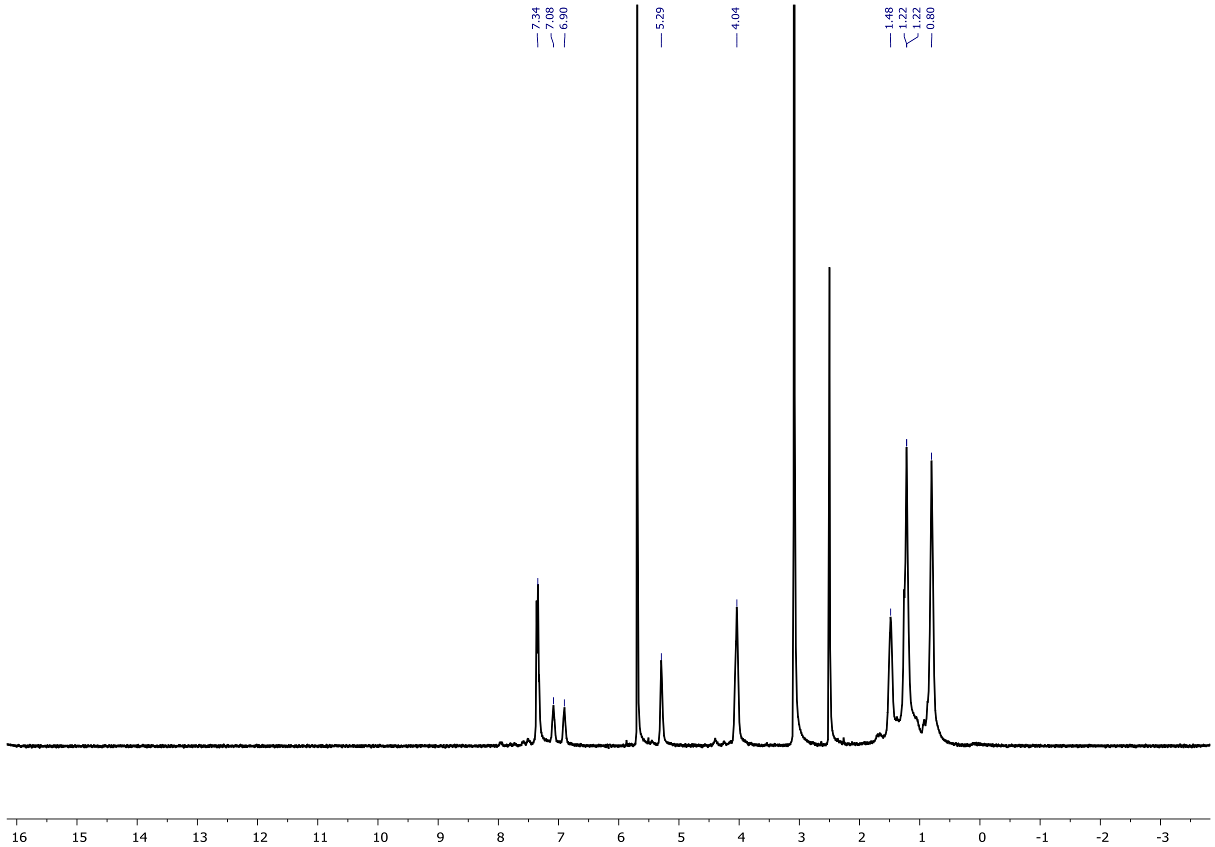

Figure S52. ^{1}**H-NMR spectrum of** ^{15}**N-urea labeled polymer 3b.**

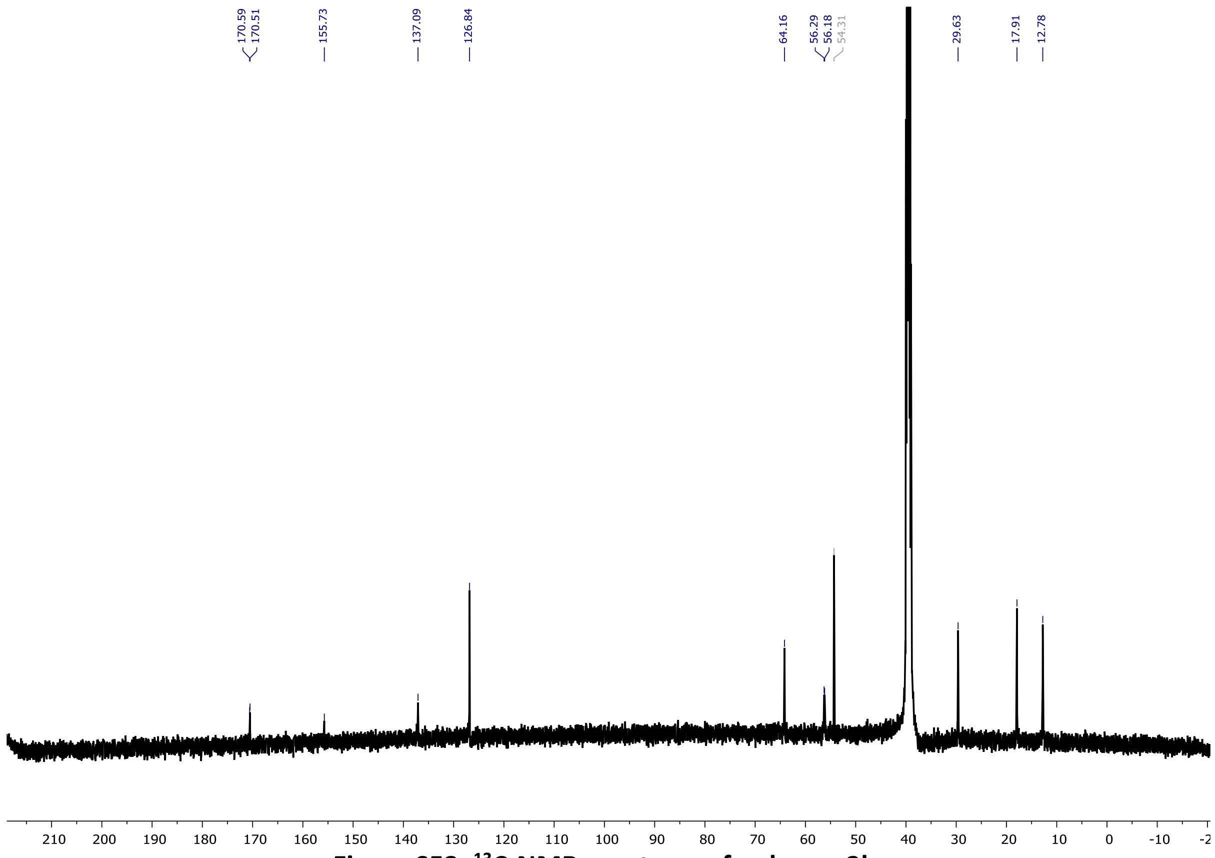

Figure S53. ^{13}**C-NMR spectrum of polymer 3b.**

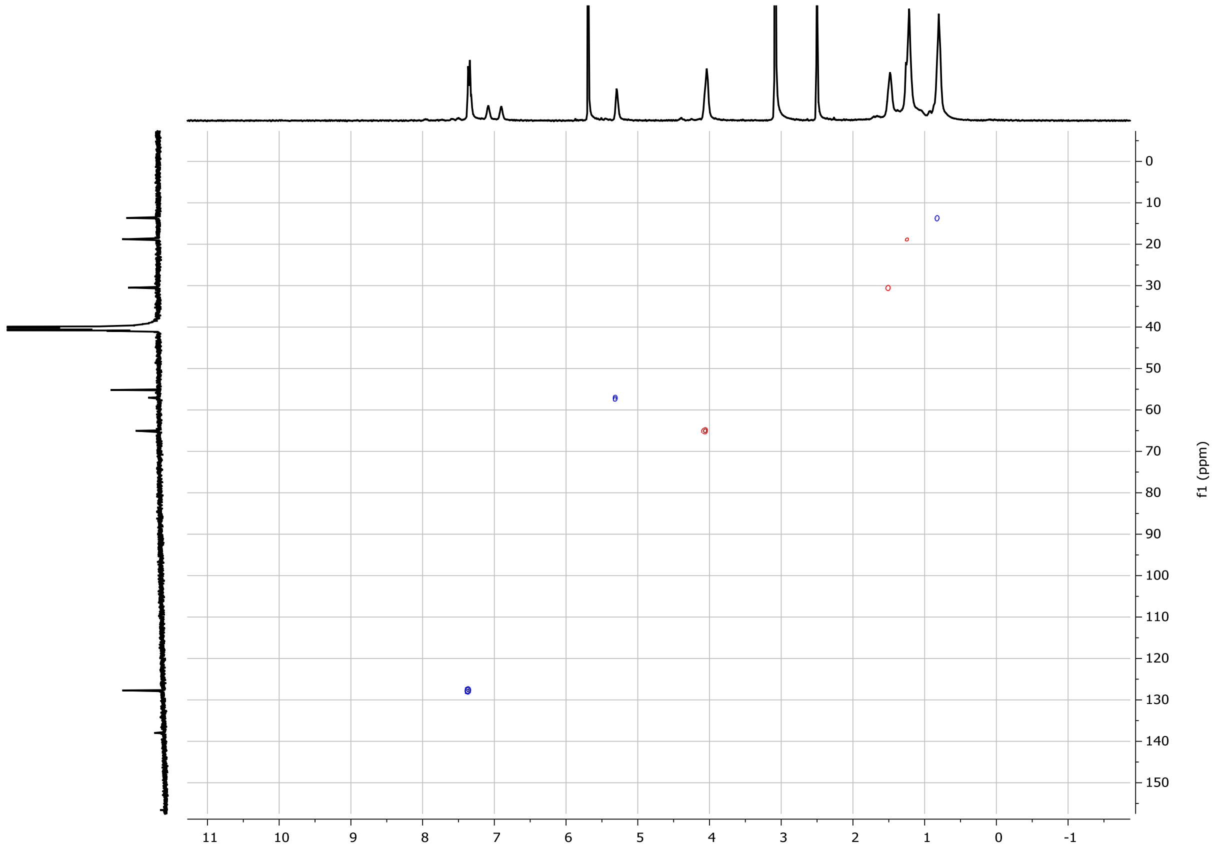

Figure S54. ^{1}**H-**^{13}**C-HSQC NMR spectrum of polymer 3b.**

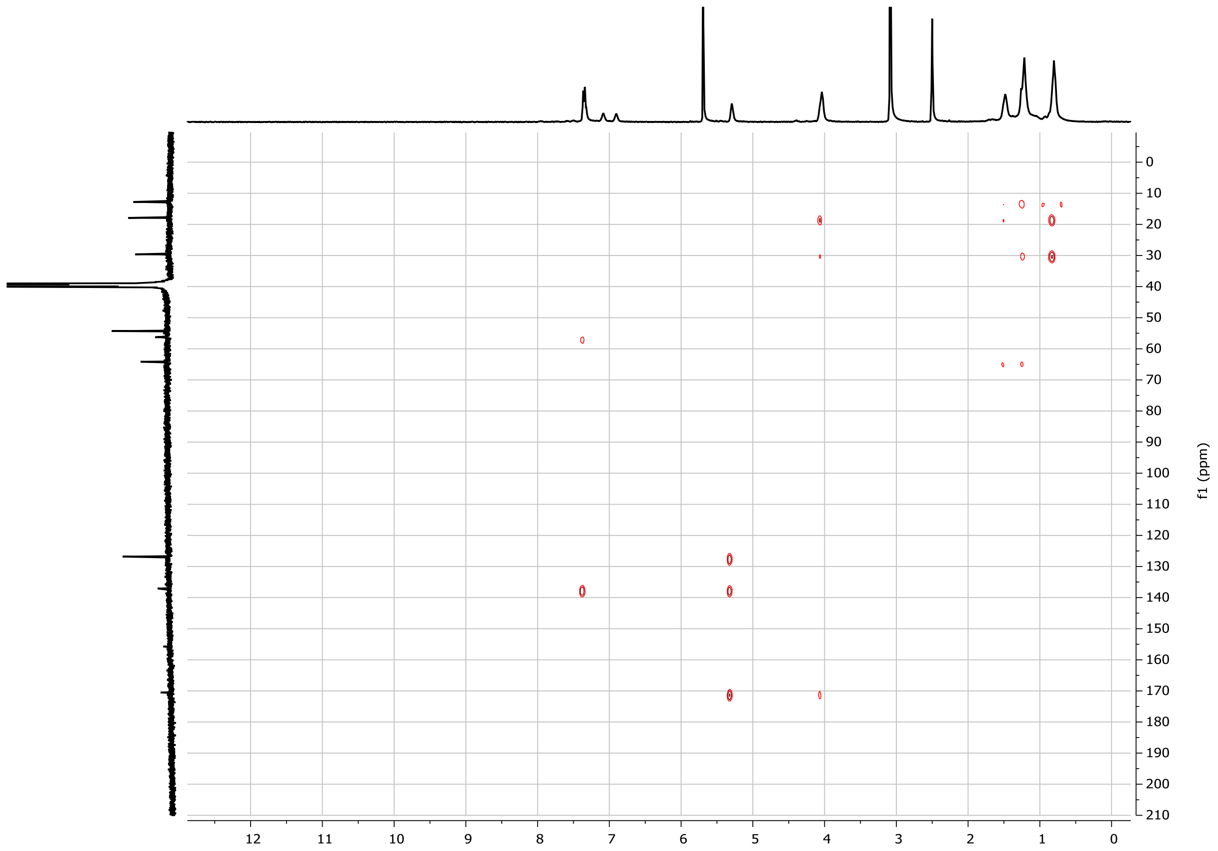

Figure S55. ^{1}H-^{13}C**-HMBC NMR spectrum of polymer 3b.**

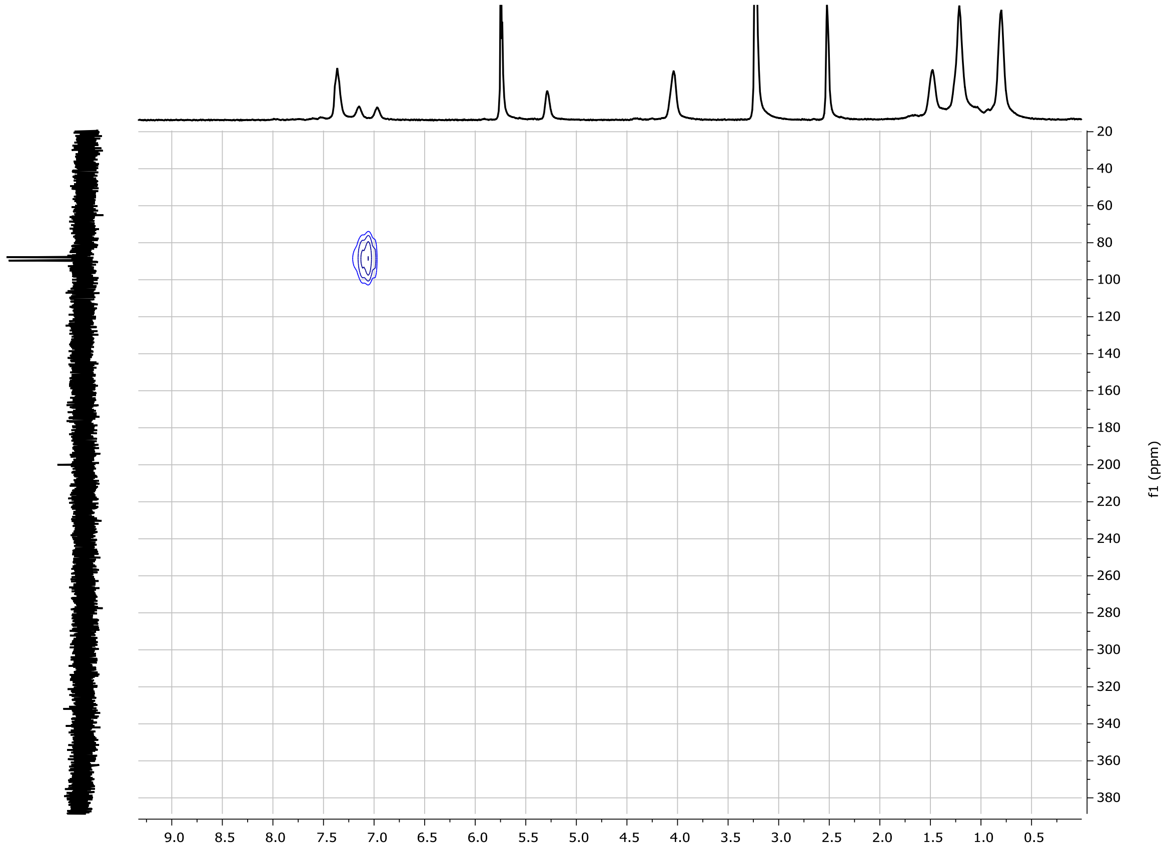

Figure S56. ^{1}H-^{15}N**-HSQC NMR spectrum of polymer 3b.**

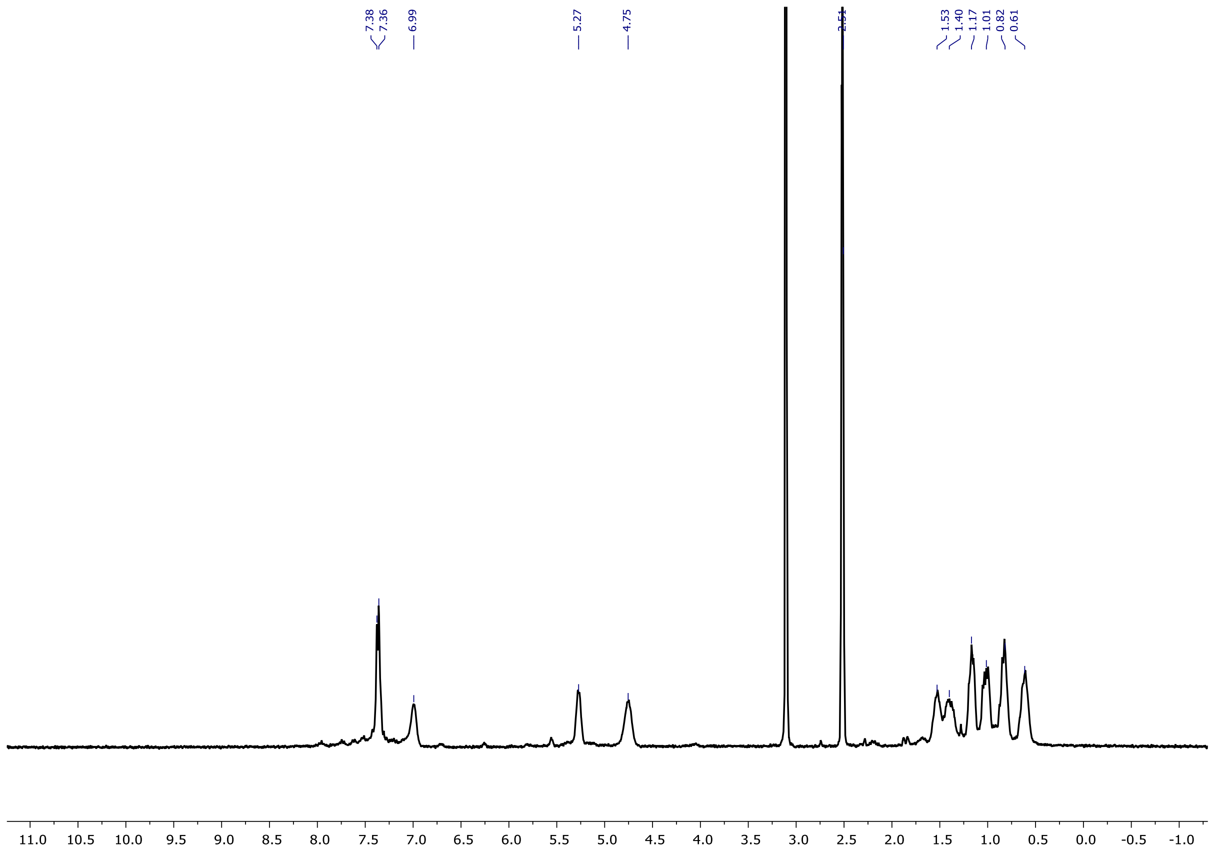

Figure S57. **^{1}H-NMR spectrum of polymer 3c.**

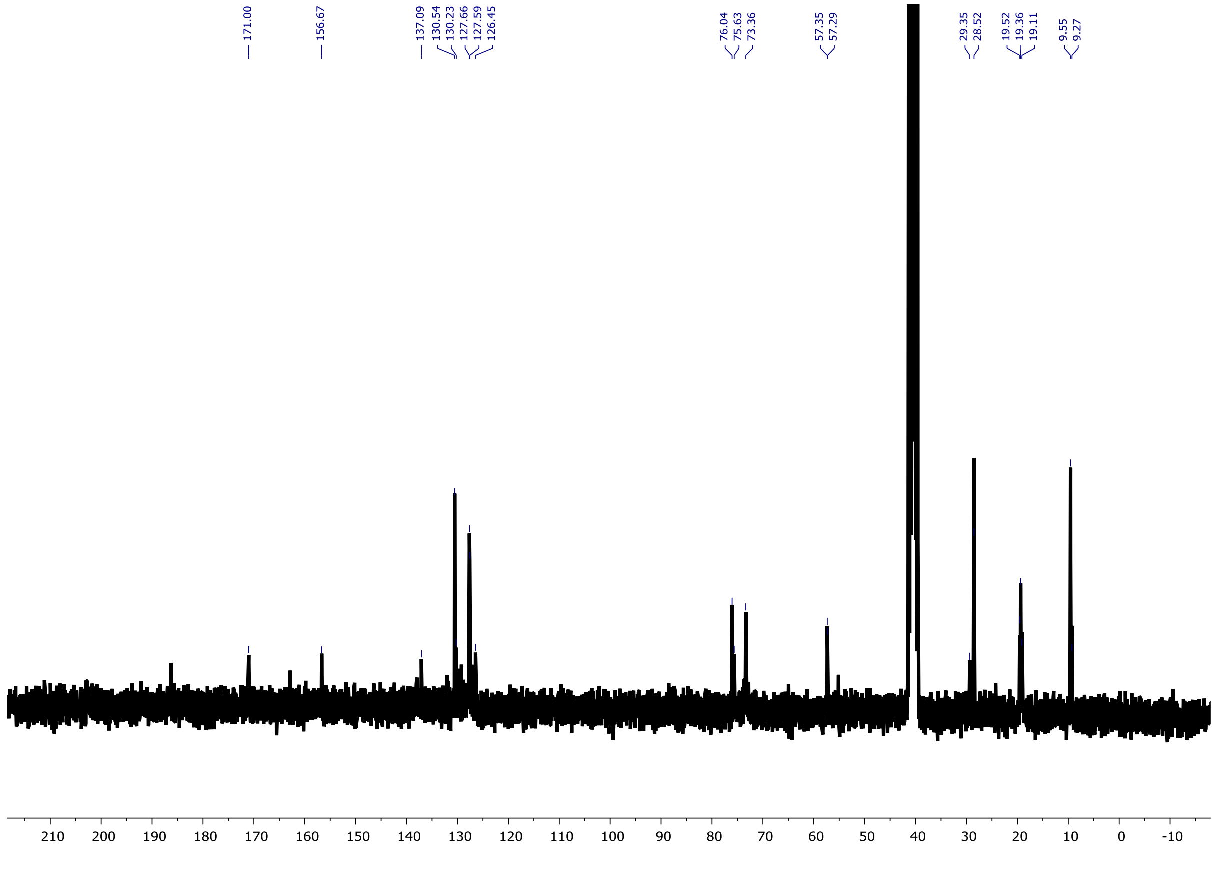

Figure S58. **^{13}C-NMR spectrum of polymer 3c.**

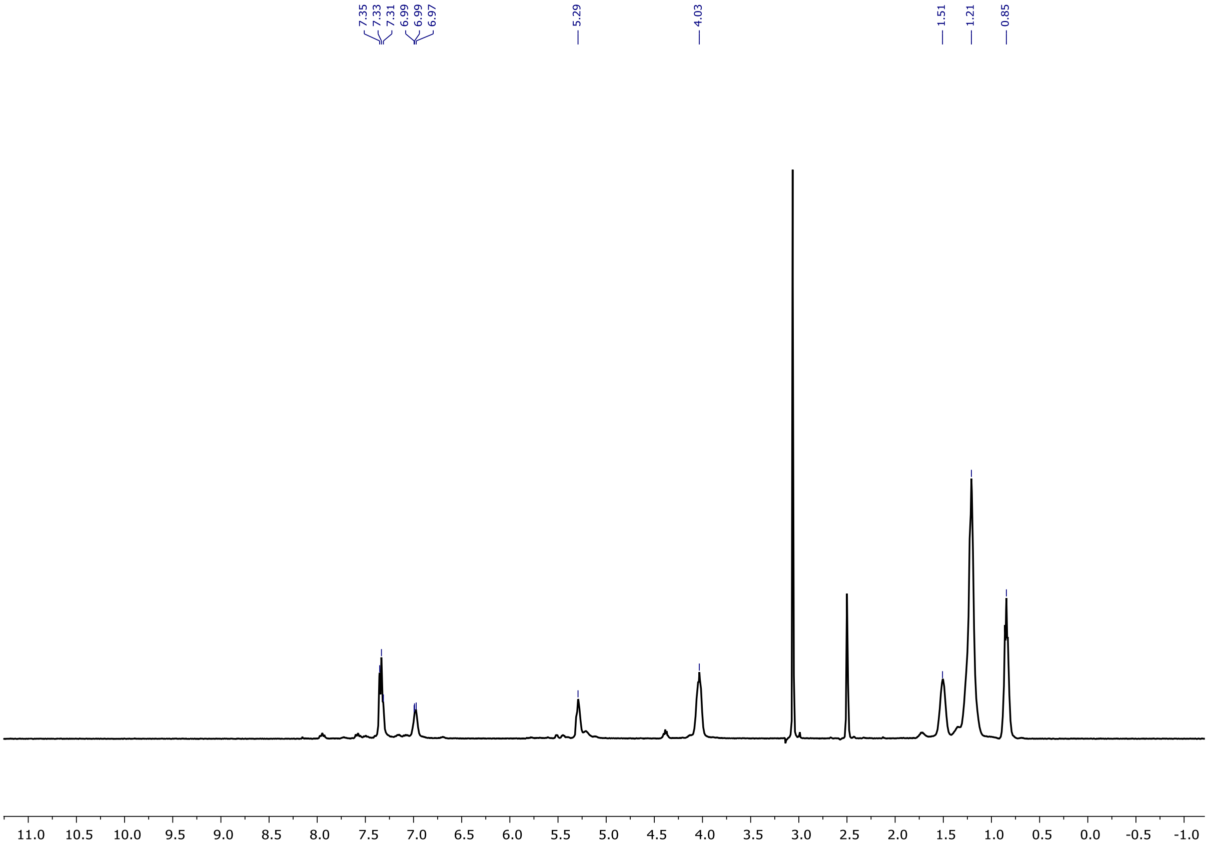

Figure S59. ^{1}H-NMR spectrum of polymer 3d.

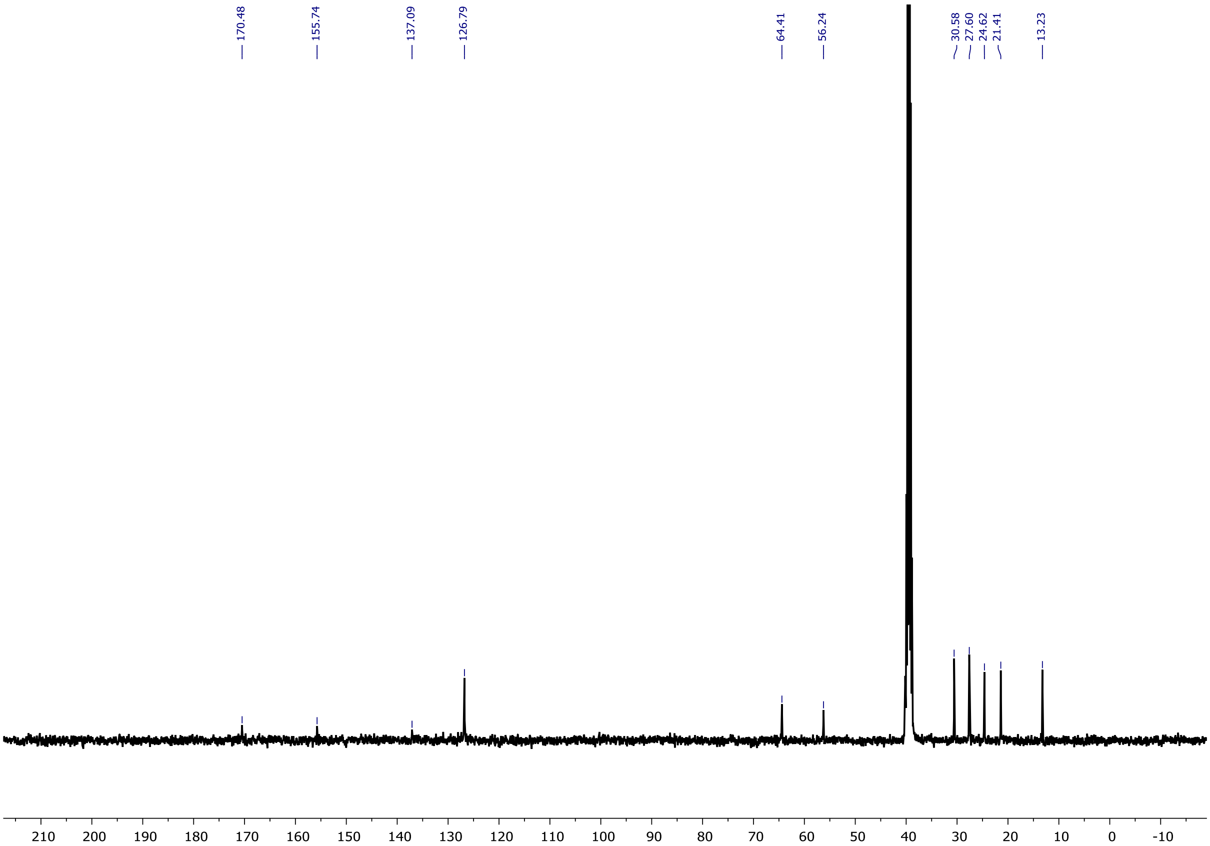

Figure S60. ^{13}C-NMR spectrum of polymer 3d.

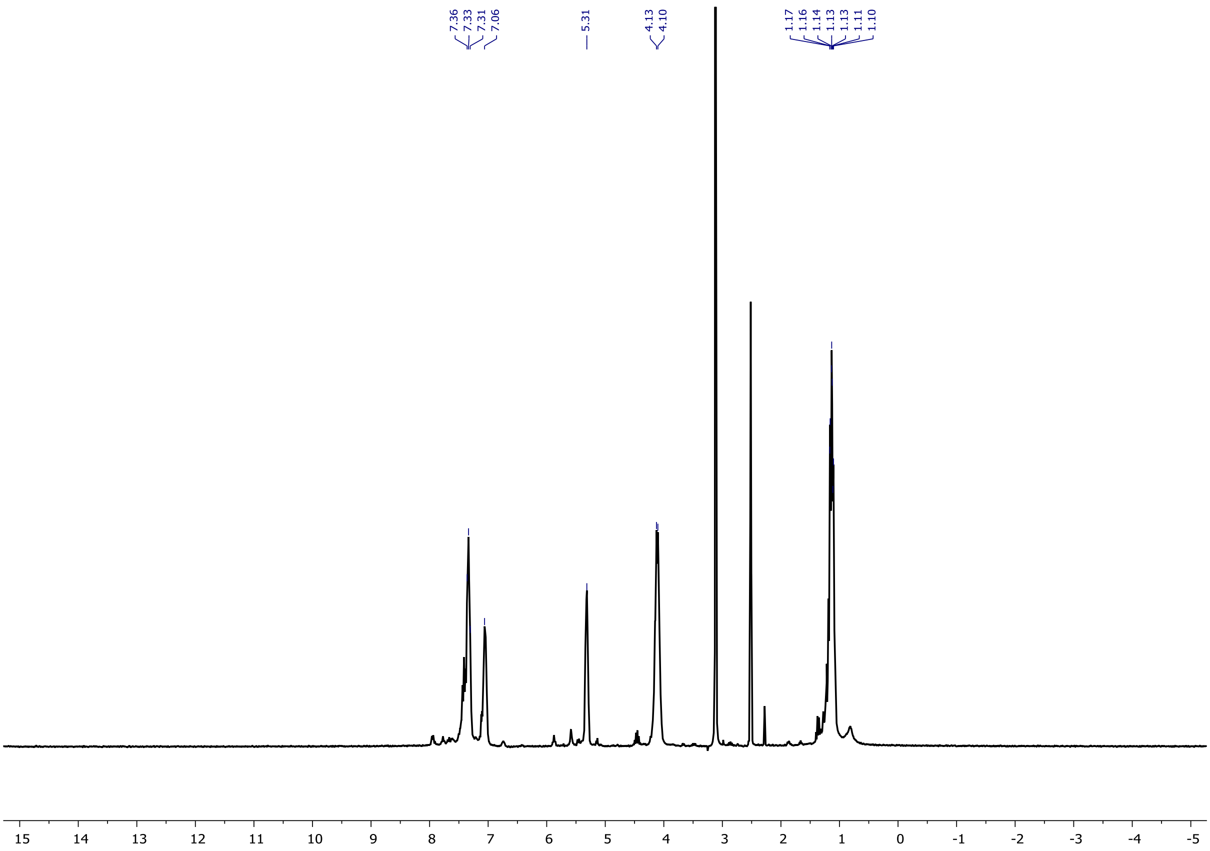

Figure S61. ^{1}H-NMR spectrum of polymer 3e.

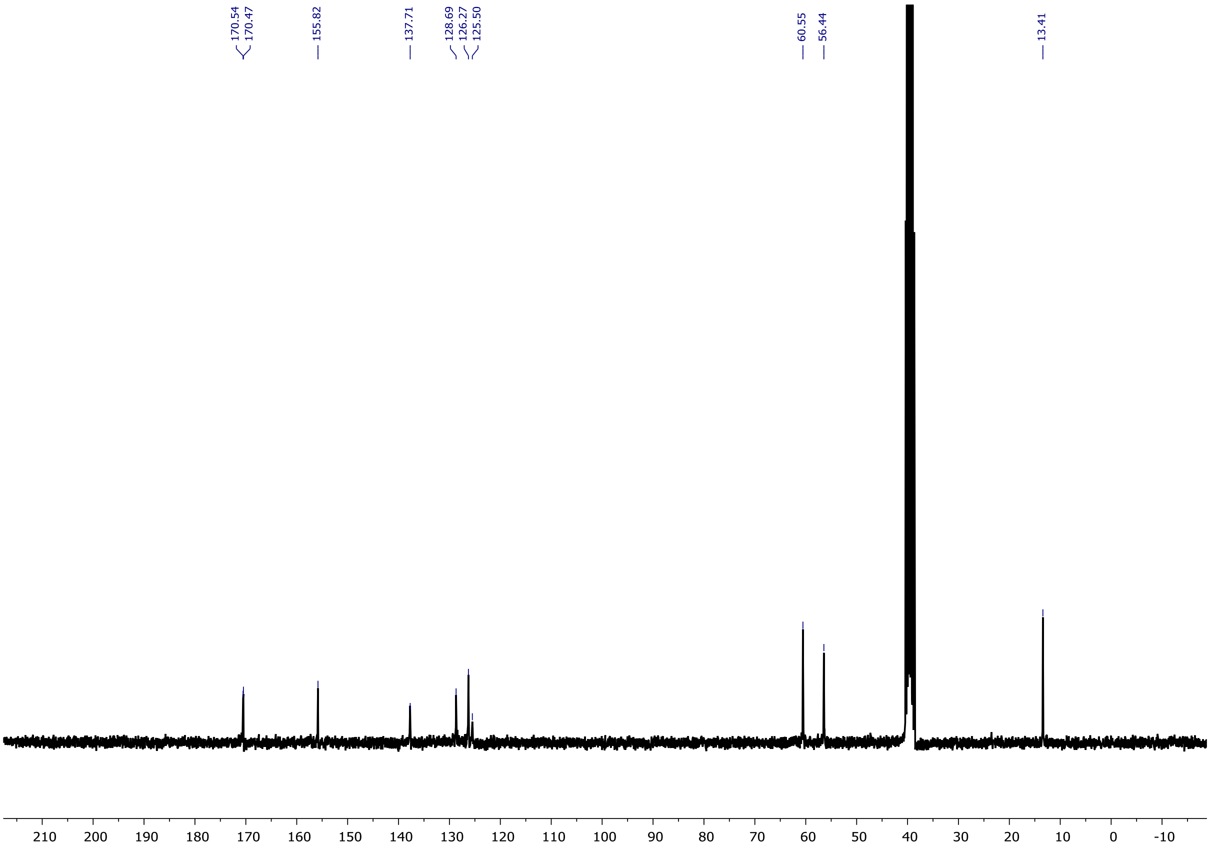

Figure S62. ^{13}C-NMR spectrum of polymer 3e.

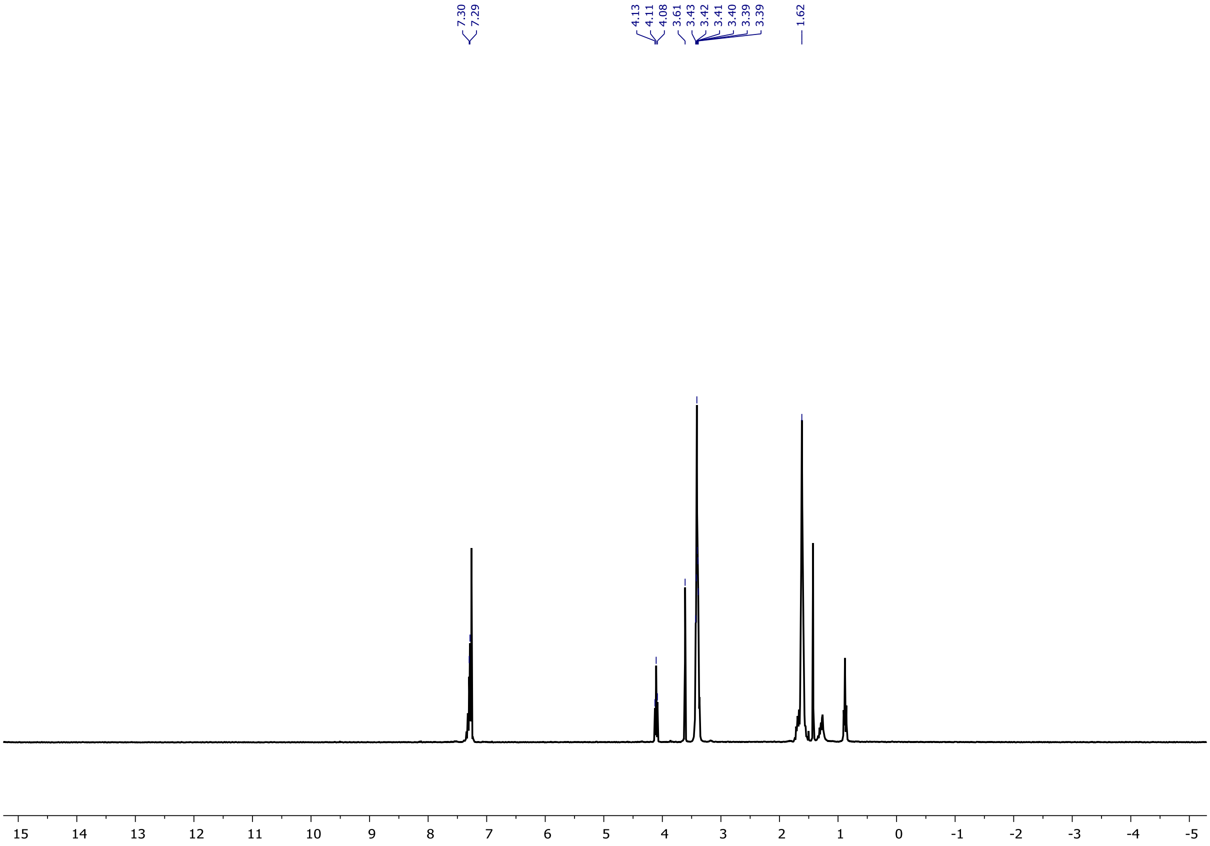

Figure S63. ^{1}H-NMR spectrum of 2-phenylacetyl functionalized polytetramethylene oxide.

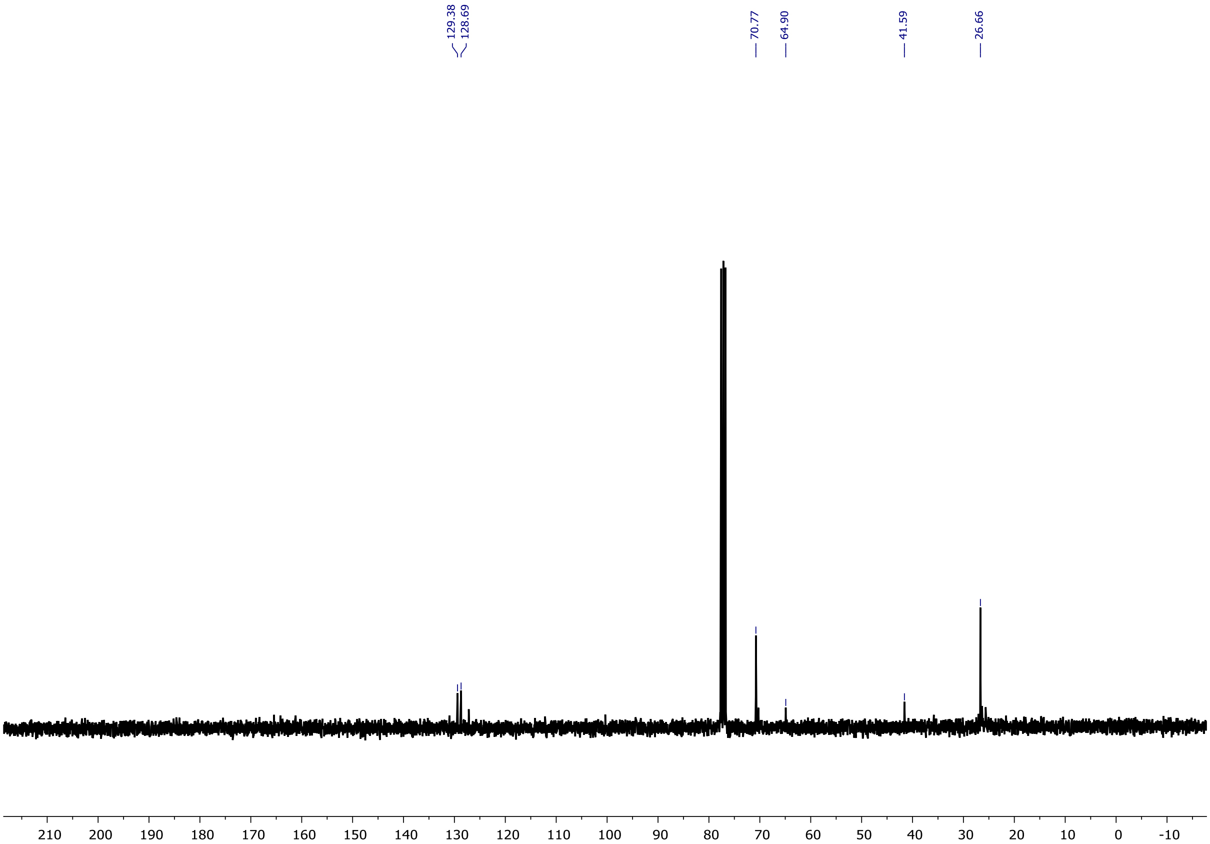

Figure S64. ^{13}C-NMR spectrum of 2-phenylacetyl functionalized polytetramethylene oxide.

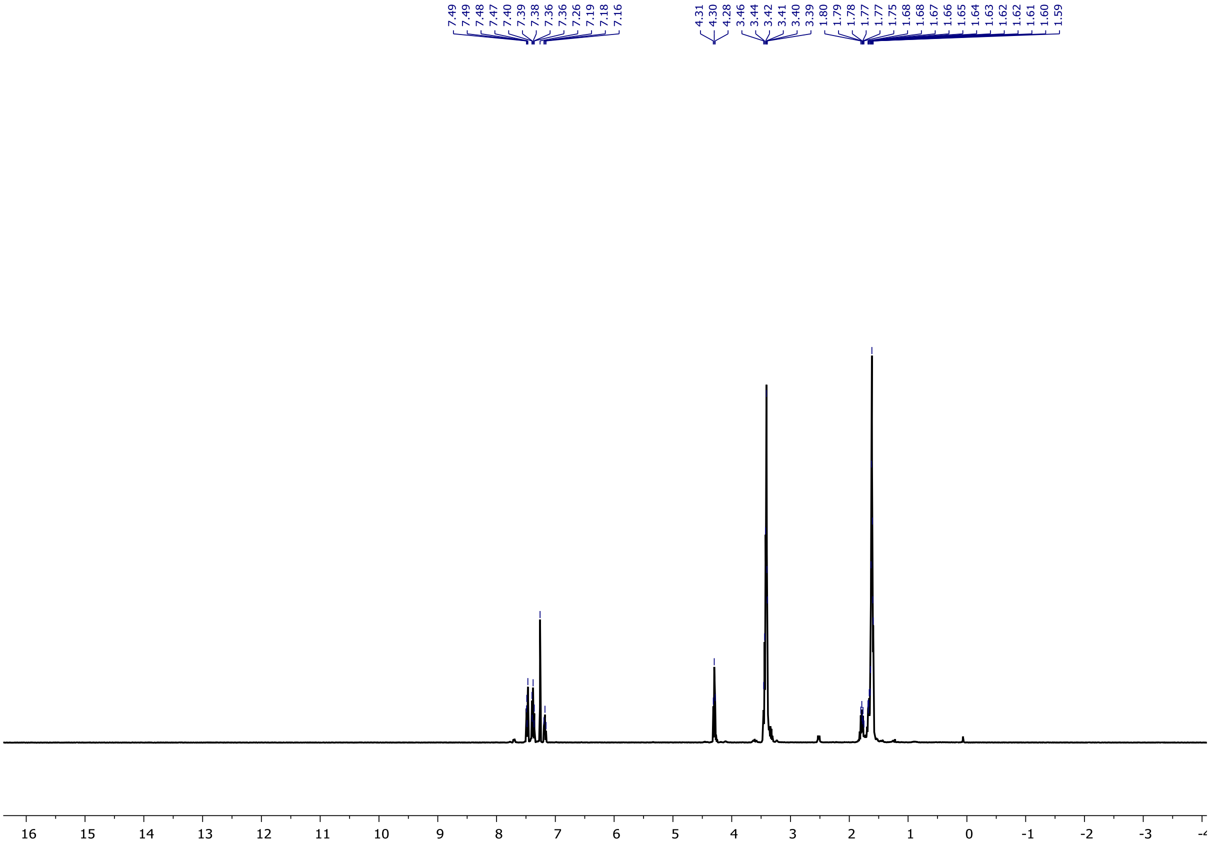

Figure S65. ^{1}**H-NMR spectrum of 2-diazo-2-phenylacetyl functionalized polymer 2f.**

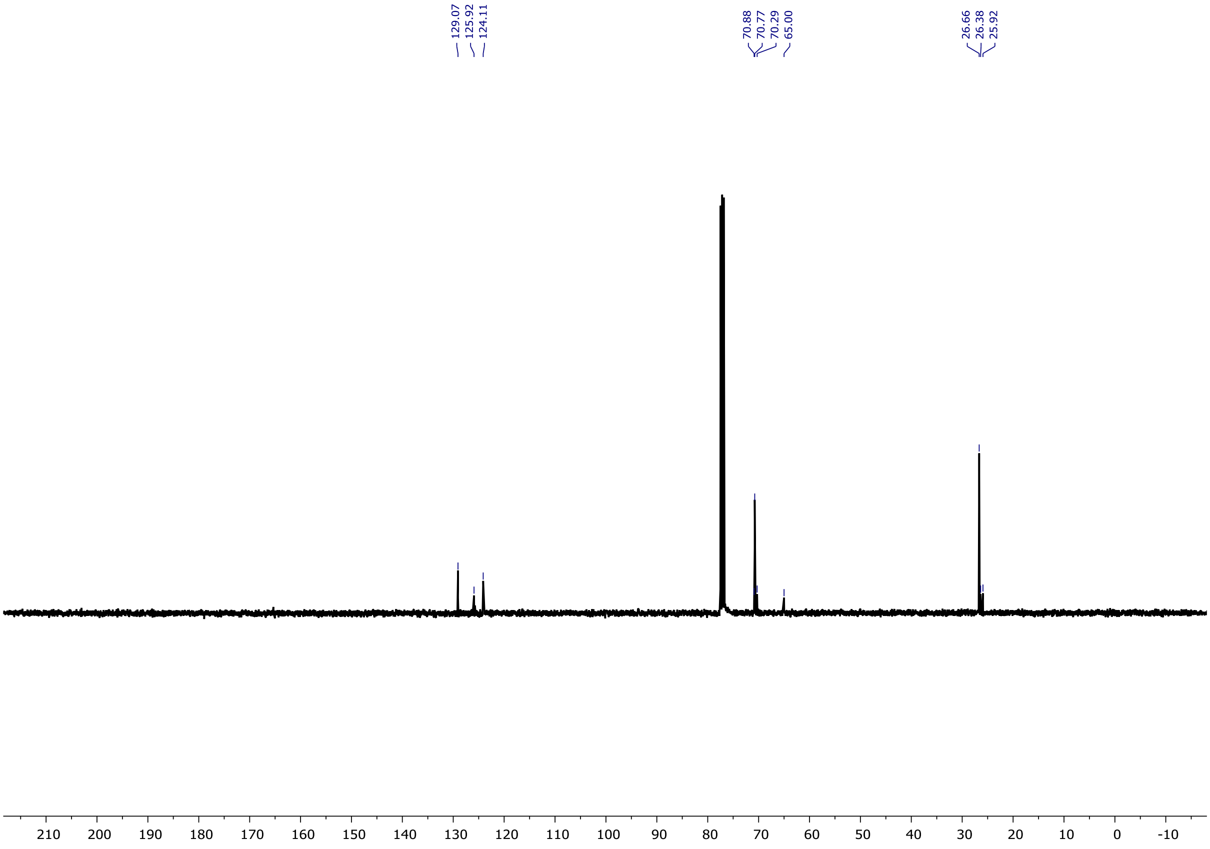

Figure S66. ^{13}**C-NMR spectrum of 2-diazo-2-phenylacetyl functionalized polymer 2f.**

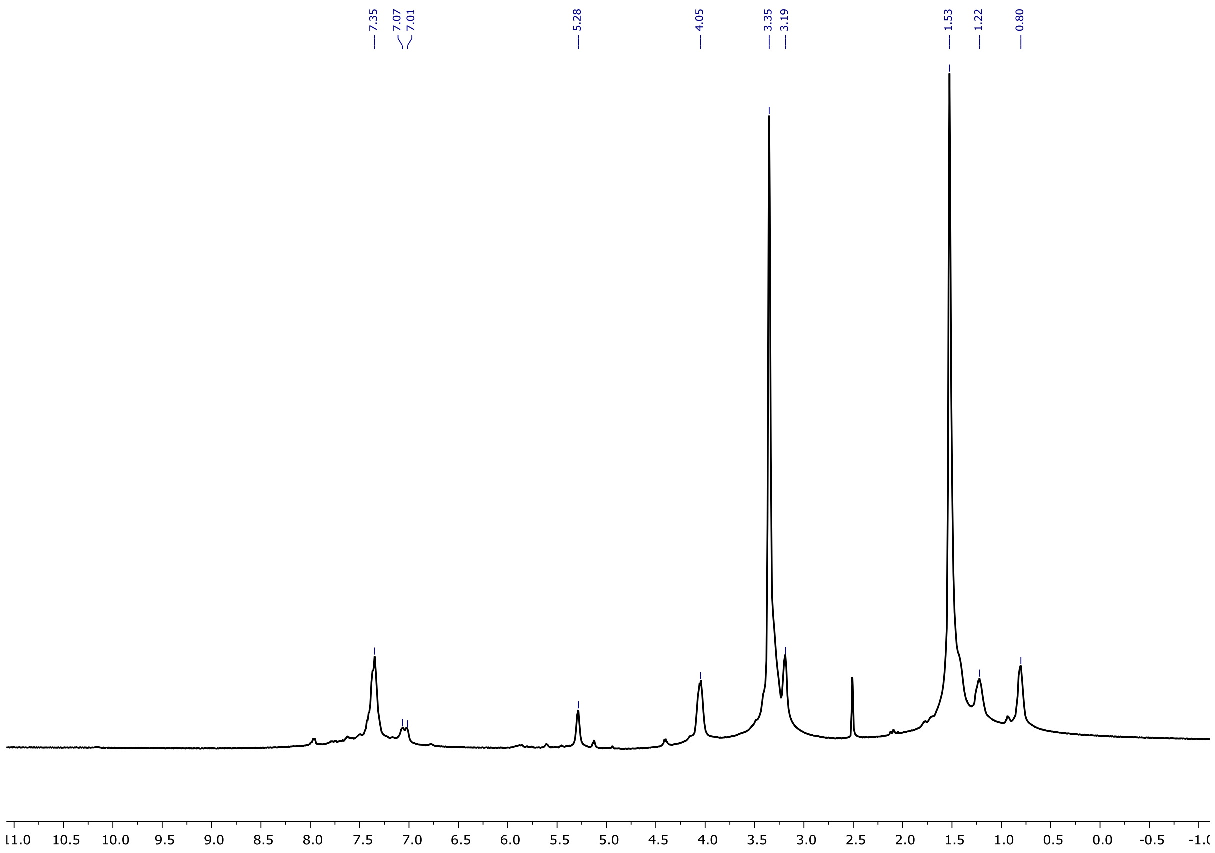

Figure S67. ^{1}H-NMR spectrum of 2a-2f copolymer 3g.

^{1}H NMR integration shows that the ratio of monomer incorporation into copolymer **3g** is consistent with the feed ratio (Figure S67). The methyl signal at 1.22 (unique to monomer **2a**) integrates in a 3:2 ratio to the N-H signal at 7.04 ppm or the benzylic C-H signal at 5.28 ppm (100% **2a** would give a ratio of 6:2, and 100% **2f** would give 0:2).

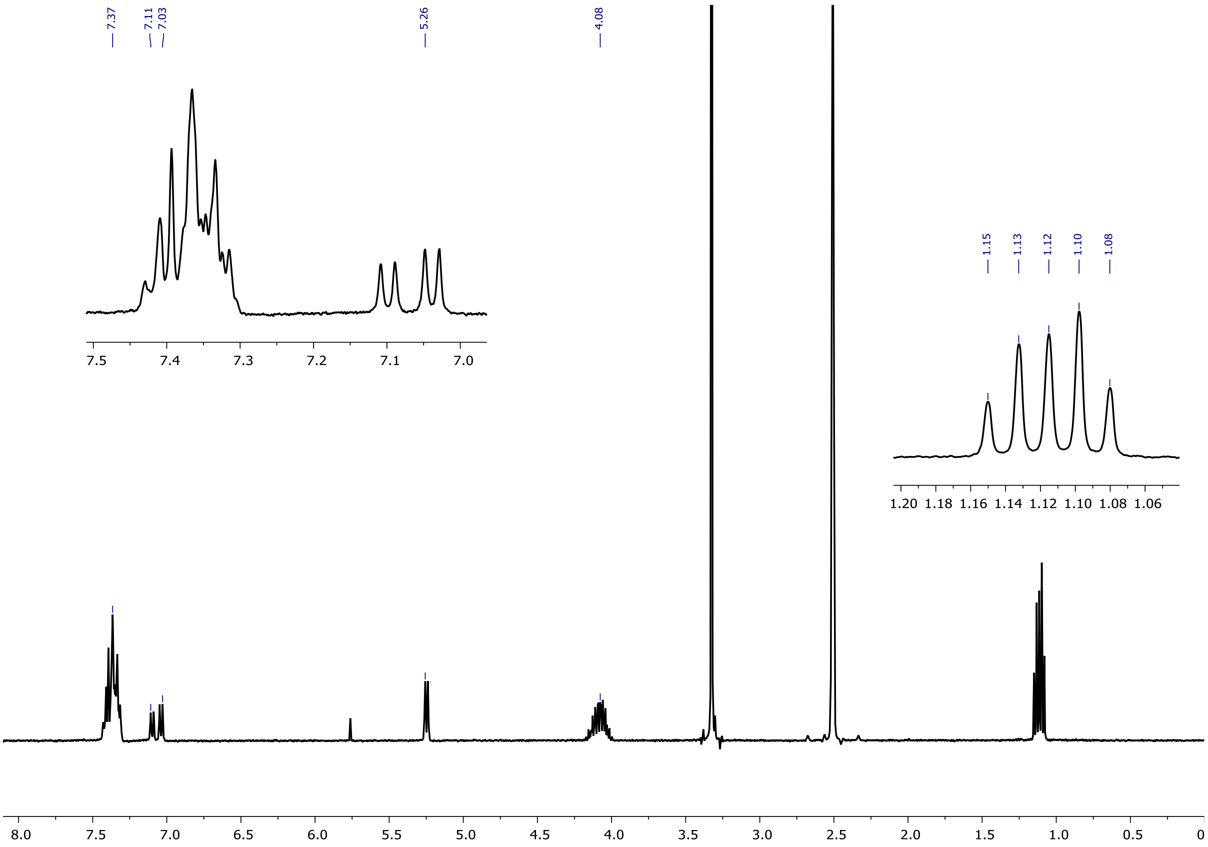

Figure S68. ^{1}H-NMR spectrum of diastereomers 5.

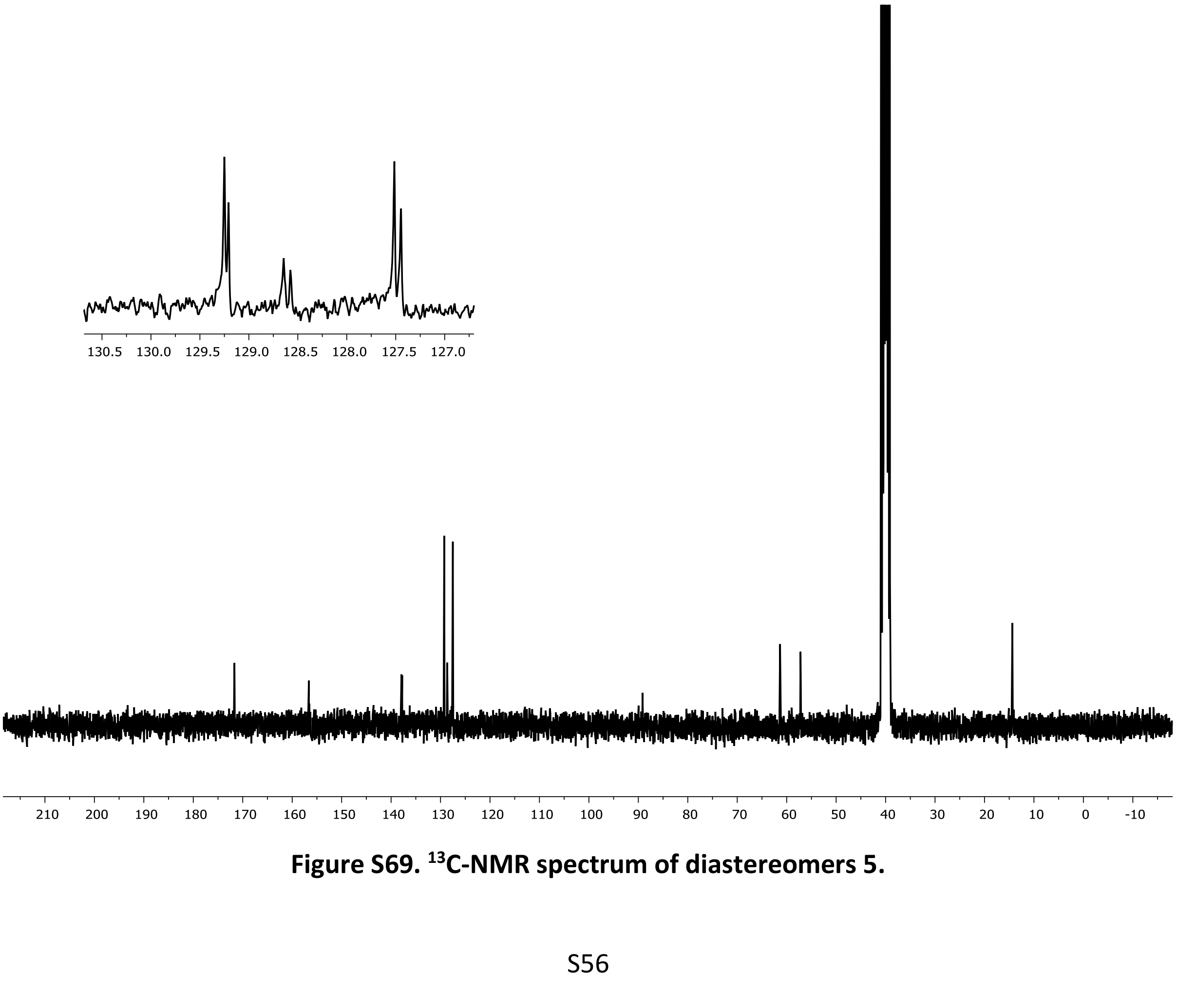

Figure S69. ^{13}C-NMR spectrum of diastereomers 5.

DFT

DFT geometry optimizations were performed on a simplified model of the catalyst (benzene instead of p-cymene) using ORCA 4.1.2.[15] The calculations were performed at the BP86[16]/def2-TZVP[17] level of theory on an m4 grid using Grimme's version 3 ("zero damping") dispersion corrections.[18] All minima (no imaginary frequencies) were characterized by numerically calculating the Hessian matrix. Transition states (saddle points) were characterized by one imaginary frequency along the reaction coordinate in the numerically calculated Hessian. The kinetic isotope effect was calculated by recomputing the vibrational corrections to the Gibbs free energy of the relevant minima and transition states using a mass of 2.014 amu for the deuterated positions.

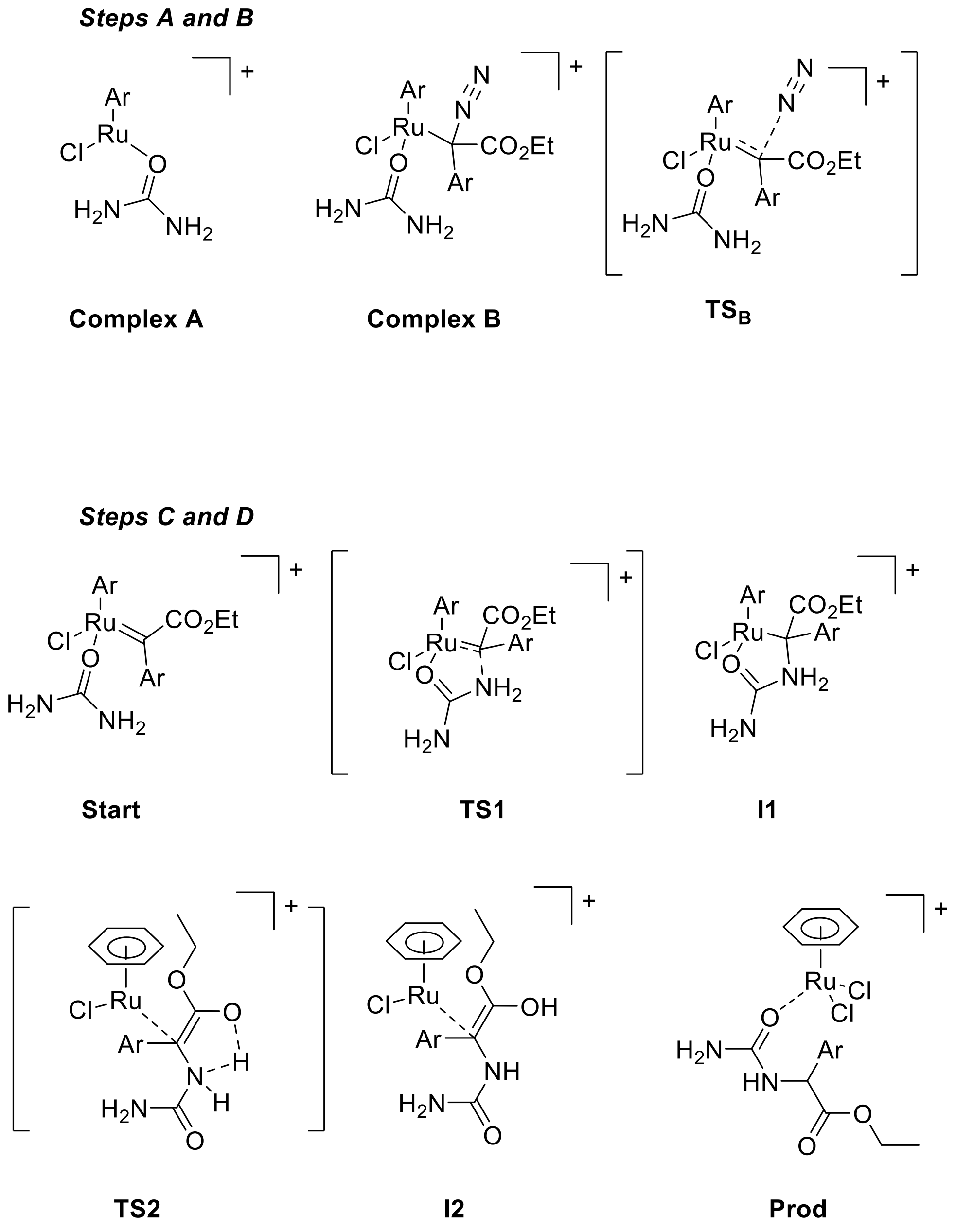

Figure S70. Structures of DFT calculated species.

	ΔG (kcal/mol)	ΔΔG (kcal/mol)	ΔΔG (Overall, kcal/mol)
**Start**	-972894.54	0.00	-38.98
**TS1a**	-972883.02	11.53	
**TS1b**	-972882.27	12.27	
**I1a**	-972885.87	8.67	
**I1b**	-972887.94	6.60	
**TS2**	-972881.08	13.46	
**I2**	-972886.50	8.05	
**Prod**	-972896.02	-1.48	-41.46
**TS_B**	-1041619.61		3.73
**Complex B**	-1041625.65		-2.30
**Complex A**	-635525.66		
**N_2**	-68767.78		
**Phenyl-2-diazoacetate**	-406097.68		
**Complex A + Phenyl-2-diazoacetate**	-1041623.34		0.00

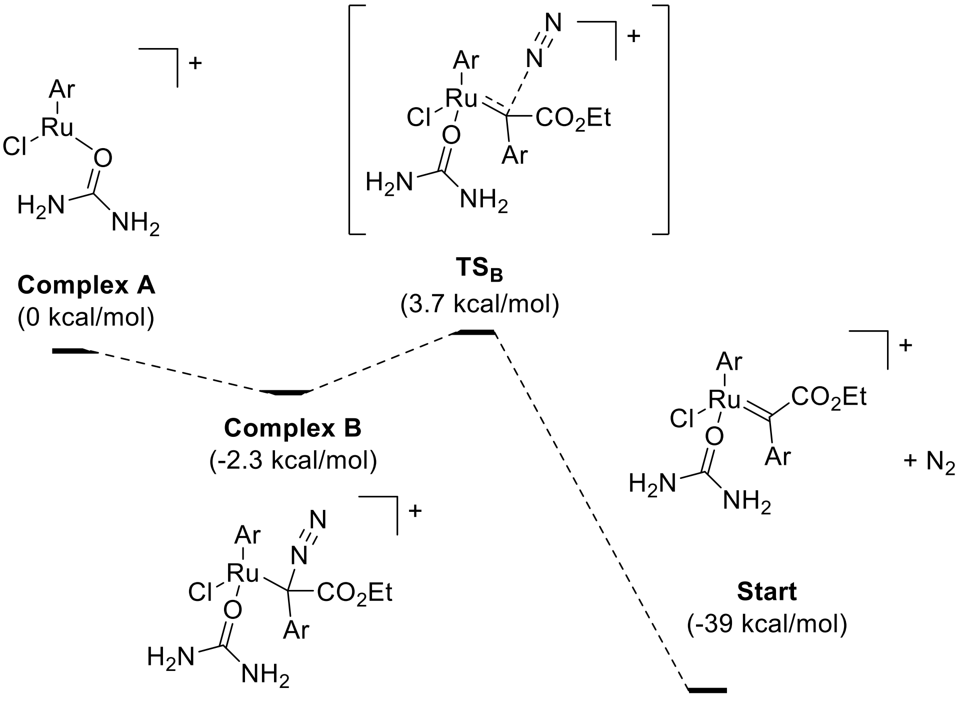

Figure S71. Calculated Minimum Energy Reaction Pathway Steps A and B.

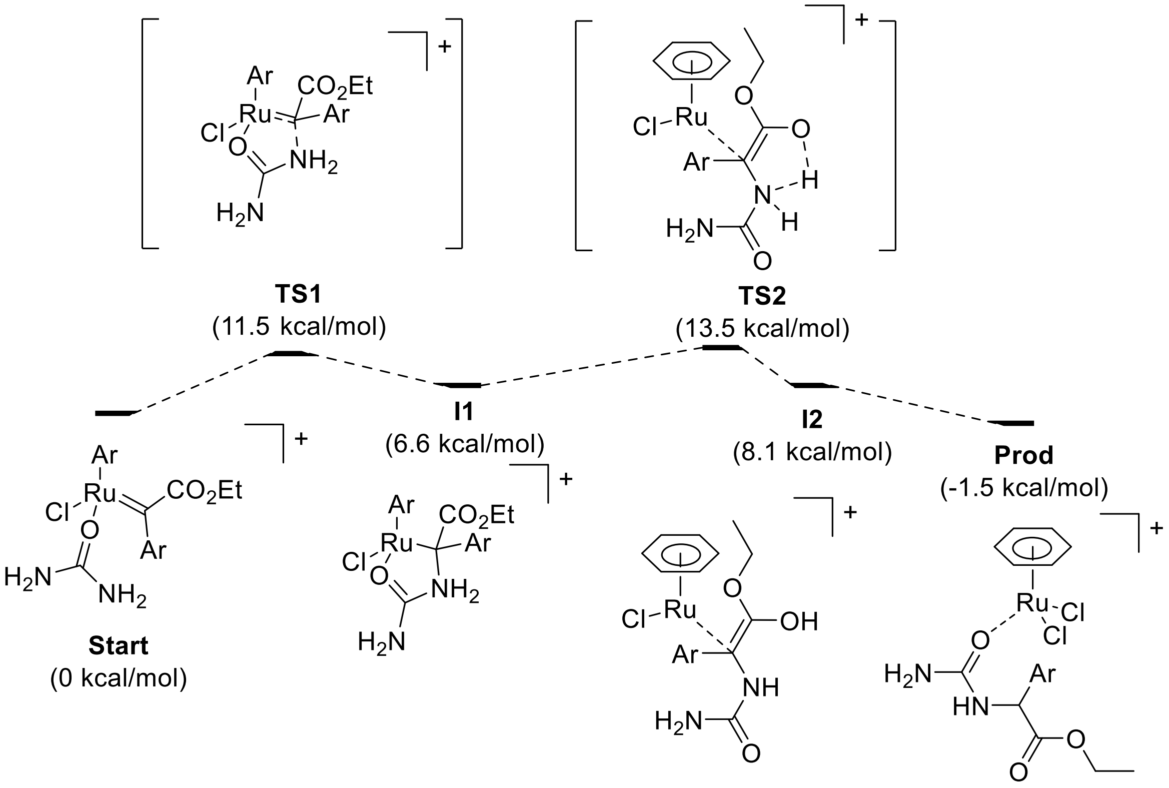

Figure S72. Calculated Minimum Energy Reaction Pathway Steps C and D.

Coordinates of all structures

Start

Ru	-3.26319527681513	-8.02777386974730	-3.03797389748552
Cl	-1.38371487930729	-8.30216538402027	-1.60121181528266
C	-3.21017779596175	-5.81695422643922	-3.81541754132658
H	-2.39367855611065	-5.31901433614246	-4.33509773859324
C	-4.13281871990227	-6.63226356529268	-4.54544339612359
C	-5.25400520710374	-7.26684443350977	-2.46162550452346
H	-6.00437090967249	-7.85373427967812	-1.93350955948917
C	-4.26046204858233	-6.58423868902745	-1.71810376524126
C	-3.25140592597848	-5.83286028487268	-2.41525952964411
H	-2.46413875415814	-5.34799853153828	-1.84043037909555
C	-5.19878406380172	-7.29935990456745	-3.88983666821689
H	-5.92262308046700	-7.87932258351262	-4.45846900294617
C	-4.72033186883856	-10.66630019677113	-3.36810259706462
C	-6.23239914216922	-12.41515115436328	-4.97302115307109
C	-4.79555648508331	-10.48327385584209	-4.77459864900669
C	-5.42201624257835	-11.76197866118556	-2.79481798000787
C	-6.17215327698761	-12.61813072643359	-3.58866972168278
C	-5.53757116625266	-11.35017542166136	-5.56317837583724
H	-4.23993673386537	-9.66385586863319	-5.22615627316533
H	-5.37155576614117	-11.93039789555477	-1.71935040005723
H	-6.71310021348913	-13.44794651353174	-3.13402807048567
H	-5.57964234071030	-11.20557935745048	-6.64287383041431
H	-6.82079240168249	-13.09068844624175	-5.59507624965072
C	-3.96965764989657	-9.76737992957326	-2.53262569633495
C	-3.75853118994905	-10.19475805242668	-1.11718306114134
O	-2.95612760752919	-11.08018689344192	-0.87080991520589
O	-4.50075851843544	-9.52101467515007	-0.22907827194138
C	-4.24987339375319	-9.82658540184231	1.19257602135955
H	-3.98950429006600	-10.88922840080438	1.27192208678041
H	-5.21918077141217	-9.64618467440855	1.67151622737718
C	-3.15493902402484	-8.93350714001338	1.73613212804888
H	-2.21450171976382	-9.09139531698587	1.19421463362634
H	-2.99142873907899	-9.16774136023171	2.79764947648596
H	-3.43087378252688	-7.87298741755816	1.66197866874536
O	-2.10757191635491	-8.85002515166872	-4.59885189141535
C	-1.45319980939282	-9.94681269290115	-4.63720415834352
N	-1.05383148086275	-10.38817880921822	-5.85960968033568
H	-1.20942525355777	-9.75962071315227	-6.63934471897084
H	-0.28417396048970	-11.04235515152698	-5.94510211379277
N	-1.20691094797047	-10.69815667214275	-3.55836871330142
H	-1.28650276004814	-10.25349188104992	-2.63540639924661
H	-0.72845170855845	-11.58796607383096	-3.63088125149983
H	-4.25198001852097	-6.63867114523663	-0.63282842819561
H	-4.02326460214847	-6.73251626081838	-5.62485584428658

TS1a

H	0.23360415024834	1.02051666644230	2.06611509267161
C	-0.35564768433624	0.47863673789451	1.32723022851443
C	-1.70476446631707	-0.83016287875855	-0.78982959065097
C	0.08105557009448	-0.83619448107414	0.90685341354418
C	-1.45946992239929	1.10575270553116	0.73767940381496
C	-2.06321159310250	0.47894011913856	-0.40024569763968
C	-0.60100177771728	-1.49051962773732	-0.13019534131794
H	0.93922888782456	-1.29388846173037	1.39324005207313
H	-1.74212464777052	2.12262217976519	1.00089442948243
H	-2.82015679830918	1.00365324113327	-0.98035352069451
H	-0.24333891592899	-2.45998840026635	-0.47184879320925
H	-2.20847240480270	-1.32353272107910	-1.61830148214759
Ru	0.08573913257000	0.46619522430433	-0.91053087762377
Cl	-0.54517198718446	2.14676749117500	-2.47838340403499
O	1.86278664072757	1.47649202734578	-0.29908566114242
C	2.32152853945646	2.65684916683015	-0.36152230637911
N	1.64964883993009	3.68541200183666	-0.91150112738979
H	2.13810283479042	4.54281572673979	-1.14535962916287
H	0.85899096091881	3.42290716184674	-1.51829715606446
N	3.51646619846180	2.89018398260134	0.24649152221593
H	4.02389847011445	3.75156155554610	0.07991236743827
H	4.06441914168794	2.06867880680432	0.47641845962606
C	1.14550590938571	-0.42530356034380	-2.27367721951243
C	2.46946596922234	-0.91402323998782	-1.76847629788310
O	3.49555605946268	-0.27272658464529	-1.90895813993314
O	2.35579006804803	-2.10878096387667	-1.15877672652589
C	3.58468459480177	-2.69596761768339	-0.60340604634535
H	4.28028174743341	-1.87679057885022	-0.38539567028351
H	4.01827863818965	-3.32413127764052	-1.39313165292488
C	3.20979897526258	-3.49893512913097	0.62731121958760
H	3.97276113355794	-4.26515791424217	0.81880564286041
H	2.25274620406151	-4.01714615652227	0.47913727129220
H	3.13482778456875	-2.86493366964055	1.52054012994770
C	0.87414859363052	-0.63822336204304	-3.66168051085539
C	0.36200739390361	-1.04899231434426	-6.39660567686936
C	-0.43142318639505	-0.46094696221594	-4.19467731187011
C	1.91555486170779	-1.04147259029501	-4.54761085736215
C	1.66113079943185	-1.22251089173410	-5.89733397016268
C	-0.68270546386253	-0.67919447278186	-5.54109227887119
H	-1.23583190300876	-0.16853092260146	-3.52628216998861
H	2.92849043484771	-1.16342306005674	-4.16602497551416
H	2.47066021981859	-1.50552009578410	-6.56991770123973
H	-1.69148389089753	-0.55260532160333	-5.93343992539153
H	0.16464588787159	-1.20638153826556	-7.45768751407670

TS1b

Ru	-3.45343672471802	-7.97227749926195	-3.16026322215348
Cl	-3.05015218268931	-7.16707673125006	-1.00511104824568
C	-3.06041394605347	-6.16126779966627	-4.85207337477690
H	-2.16511932101018	-5.65046998288321	-5.20412395070290
C	-3.37027037492628	-7.48994451555407	-5.34332679063540
C	-5.44433192143704	-7.51305239811259	-4.05439903890407
H	-6.37323600629321	-7.99726674017485	-3.75887746023555
C	-4.95781172465438	-6.38667205044386	-3.33776704524682
C	-3.84643232289518	-5.61413163296551	-3.86045153318039
H	-3.58699646711501	-4.66575876924603	-3.39221756632485
C	-4.64081562684622	-8.06637470500508	-5.08063673423688
H	-4.94709124291903	-8.98437167038525	-5.57854042335791
C	-5.28238682448533	-10.33607815009936	-2.87247488369224
C	-7.69107144742887	-11.64683929641142	-3.44557614282932
C	-5.40033724571975	-11.12408265832363	-4.03023496456210
C	-6.37224423932988	-10.22846403090932	-1.98793085808010
C	-7.57183982264504	-10.87434647791727	-2.28634550704860
C	-6.60167068387393	-11.77653799443002	-4.31048332363869
H	-4.54283350942347	-11.23410053355128	-4.69517934848592
H	-6.26984730785174	-9.63220949256403	-1.08101052440711
H	-8.41764353032770	-10.77516696606544	-1.60532212626958
H	-6.68489879201698	-12.39020000425150	-5.20794303812163
H	-8.62972128037169	-12.15326797998041	-3.67008677847686
C	-4.01871314990123	-9.64995198928195	-2.53920140991253
C	-3.17015254386194	-10.45972729959622	-1.60599247713546
O	-2.30184070644097	-11.20295476794776	-2.06090600966634
O	-3.50088599809527	-10.33007799919120	-0.33831287089805
C	-2.69966713683908	-11.08626643141216	0.64662759341267
H	-1.64422679569104	-10.84583334747200	0.46341058152837
H	-2.85770725053648	-12.15522130141459	0.44952931656034
C	-3.16779660113680	-10.66172192502999	2.01709010459369
H	-3.02928613573494	-9.58273409858869	2.16197529510619
H	-2.57904828839521	-11.19179692153988	2.77852918491511
H	-4.22636124580627	-10.90862332505481	2.17052817525304
O	-1.39362177226834	-8.33887818339643	-3.08996315904932
C	-0.66892112469940	-9.06685762451572	-3.83937718940168
N	0.57102558374381	-8.61048264635426	-4.14125620566257
H	0.85526547239517	-7.74418779071479	-3.69579739039437
H	1.30571642642583	-9.24296349722447	-4.43828179547441
N	-1.10530715087774	-10.23101385342853	-4.37427478247197
H	-1.74615195710026	-10.74772165888428	-3.74704085080962
H	-0.43726584940440	-10.80381705081352	-4.88230316698428
H	-5.50767505321771	-6.01515698222055	-2.47535833416043
H	-2.70189617752433	-7.96882522646142	-6.05766192573423

I1a

Ru	-3.14147317233334	-7.83257695863129	-3.13734726687366
Cl	-1.05544469027033	-8.06725789236557	-1.94780174078780
C	-3.35166222292562	-5.68775857221255	-3.83292741495119
H	-2.64291005640230	-5.09941796861579	-4.41237623701184
C	-4.27658817105814	-6.56145284640494	-4.49312346888750
C	-5.09731255094978	-7.38920522447526	-2.33623530359734
H	-5.69981580648276	-8.09032992044656	-1.76228090783294
C	-4.11688944930899	-6.59966244622909	-1.67238338051853
C	-3.26918056742909	-5.72198320562412	-2.43007166649916
H	-2.47982072388465	-5.17137064353840	-1.92029822974176
C	-5.19677481087067	-7.35801866026573	-3.75786166803374
H	-5.89655960402292	-8.01220392607286	-4.27333040971747
C	-4.40849583659027	-10.71085670308391	-3.48849401423817
C	-6.35115102013360	-12.23410349067005	-4.85575472283896
C	-4.73539910124246	-10.43117225137571	-4.82891668569118
C	-5.07000437317089	-11.77653823322761	-2.85383413635726
C	-6.03831603701020	-12.52382973735988	-3.52723491431308
C	-5.69079519474201	-11.18790234235044	-5.50613127605264
H	-4.25310870447337	-9.60060970567189	-5.34225262415316
H	-4.83378671282920	-12.03127177591464	-1.82047625976403
H	-6.54571014016611	-13.33798051828667	-3.00928122769229
H	-5.93017946386582	-10.95130312835909	-6.54343465805265
H	-7.10651218928328	-12.81771118457699	-5.38216353565781
C	-3.33290111385703	-9.93293534514867	-2.76336009325128
C	-3.24788915928818	-10.28320894265510	-1.28646907686754
O	-2.35434973255838	-10.97968055246990	-0.82794456829544
O	-4.26357992698553	-9.76864272444067	-0.58165144419327
C	-4.24195299393512	-10.03678185037134	0.86503960522486
H	-3.84922682606014	-11.04905002014927	1.02046122195187
H	-5.29932827873449	-10.00052270592574	1.15163859248397
C	-3.41057035218095	-8.99375763300522	1.58264420652674
H	-2.37243652404636	-9.00637627162630	1.22727542911837
H	-3.40816539637619	-9.20767996818342	2.66058842035996
H	-3.82644008468010	-7.98689222156991	1.44092185605636
O	-2.04248175203954	-8.72799399797789	-4.81357866723687
C	-1.67877982909229	-9.89444755370786	-4.60349656019777
N	-1.05148272786638	-10.67413807760295	-5.48241516385833
H	-0.82898731101308	-10.29415674081590	-6.39861761913975
H	-0.75886439868682	-11.62102251905821	-5.26814480018928
N	-1.92737929355044	-10.42617928560767	-3.27809822306656
H	-1.27667704202301	-9.84355330726737	-2.61139618327787
H	-1.78599815991632	-11.43750605612846	-3.13887188254106
H	-3.97837429386437	-6.69526450287597	-0.59779633733653
H	-4.26136420379854	-6.63046438765052	-5.58112996300569

I1b

H	0.91644100296724	-1.23883300634858	1.24207892075608
C	-0.01867999541811	-0.83029260198099	0.86637416920639
C	-2.39105468199131	0.35475628827484	-0.17619923840737
C	-0.61424987039672	-1.35516492202445	-0.31745496206733
C	-0.59813646369170	0.31864594123013	1.47867639846201
C	-1.81979164053441	0.88306776708601	1.00096258965662
C	-1.79515377290382	-0.76129592720078	-0.84783319115699
H	-0.11670225946799	-2.15383833068257	-0.86551676501640
H	-0.07236946110579	0.80145823630292	2.30266730109063
H	-2.23002406376103	1.77977354636368	1.46052375185374
H	-2.21646191986360	-1.12293141897449	-1.78307439093073
H	-3.23709072865591	0.86203630353866	-0.63833869639043
Ru	-0.26200908128664	0.75181341931669	-0.62887080968434
Cl	-0.97016979629743	2.35055283152394	-2.29705789841679
O	1.15200945491752	2.30684283303347	-0.08900584132313
C	1.99175933087113	2.56045938440981	-0.96438758788282
N	1.83809725413782	1.84634073277284	-2.23918335005476
H	2.65547234225979	1.90650476468727	-2.86375865003847
H	0.92981208962241	2.25328760431154	-2.67768801394421
N	2.90347938714095	3.52852120164224	-0.89807041356924
H	3.67444685781452	3.58537103290428	-1.55440643252897
H	3.00001405401115	4.02408171303571	-0.01554855783529
C	1.39442702024853	0.37933220779684	-1.95609672118677
C	2.52620459714608	-0.19473647040366	-1.14012024119852
O	3.44013883067293	0.49359903063332	-0.69088763104761
O	2.41581827129286	-1.51588959656790	-0.96365550917889
C	3.48162090276155	-2.15644137027857	-0.18243031584237
H	3.56432879997384	-1.63260899072387	0.78011618334661
H	4.42630141990375	-2.01640001554847	-0.72568034160796
C	3.11227203293802	-3.61327555452625	-0.02990394157134
H	3.89890244856938	-4.13060753656692	0.53591273973320
H	3.01599771245883	-4.10247469605766	-1.00786131320223
H	2.16777960943931	-3.73256754947087	0.51842764244209
C	1.21945156232331	-0.30802123068119	-3.29068763824479
C	1.01320705546293	-1.54650891742790	-5.80788022723005
C	-0.02815281131943	-0.46084139774958	-3.90890952133257
C	2.36554826572127	-0.77907904665966	-3.95932623005556
C	2.26555274449227	-1.39205276693680	-5.20726356773330
C	-0.12977057481485	-1.07843351695314	-5.15651081659287
H	-0.91654131891385	-0.07230443417355	-3.41705900227580
H	3.34976596020772	-0.68228337440442	-3.49290107638424
H	3.16337638194010	-1.75655904145931	-5.70692080734843
H	-1.10882241255797	-1.19016392009010	-5.62319653987029
H	0.92995546368491	-2.03083920497244	-6.78105345539580

TS2

H	0.81392024406436	-1.29634765043068	1.32196810795994
C	-0.11480399559081	-0.90227875978465	0.91653816234858
C	-2.47409210207361	0.23645332319531	-0.21300491474368
C	-0.68177210609635	-1.46160813730892	-0.26535129130774
C	-0.71420963511901	0.25811923456377	1.48357989534757
C	-1.92818153035630	0.80383140310353	0.95847284669374
C	-1.84602585847575	-0.88713334812301	-0.84759983739729
H	-0.17683983204163	-2.27981058193047	-0.77402111522979
H	-0.21103138716439	0.76929510657042	2.30466542166302
H	-2.34995617415476	1.71376843680587	1.38064229114339
H	-2.22308955987128	-1.25934969898255	-1.79826148815449
H	-3.31027449629733	0.72725311638365	-0.70916977773925
Ru	-0.34610776287347	0.64494958147335	-0.61885325877104
Cl	-1.06104468189436	2.10943819716016	-2.40309185747378
O	0.95596728151841	2.30221351259359	-0.05993863884912
C	1.81431900453276	2.58113034651296	-0.90715746729330
N	1.75293397551978	1.82995465887408	-2.16547350783813
H	2.59239178898199	1.94316852432541	-2.75309200322803
H	0.82140337425598	2.17524797295780	-2.63262107067461
N	2.66942029159431	3.59890096352814	-0.83541124672586
H	3.48097352982906	3.65606574228877	-1.44178875080910
H	2.69361911533797	4.13108265972870	0.03061271453336
C	1.41606291246758	0.36401998038113	-1.87581808674945
C	2.59171954424981	-0.11757624249355	-1.05492448846012
O	3.50469372139737	0.64633404314727	-0.74975562037599
O	2.52135783681532	-1.40310245355651	-0.70119969701866
C	3.67596724825384	-1.99197913220025	-0.00144133709023
H	3.28803760875569	-2.32105236464997	0.97195933947171
H	4.42079605037597	-1.20377705468525	0.15483865211473
C	4.20552290102612	-3.14662939052655	-0.82340078025663
H	5.04731617489021	-3.61248881812690	-0.29215015822566
H	4.56751726462673	-2.80195002760880	-1.80117255487268
H	3.43689217163705	-3.91474300437842	-0.98146389503742
C	1.16884963725121	-0.35638018690888	-3.17728006764784
C	0.62503942243969	-1.73129144022286	-5.59500130031377
C	0.98238573158223	0.33763897495482	-4.38813375344413
C	1.09706064564179	-1.76257995492058	-3.21729212884197
C	0.82474752541339	-2.43599902608645	-4.40554256879503
C	0.71306647690595	-0.34110284603660	-5.57823765133659
H	1.01639110147193	1.42504532900021	-4.44217831336526
H	1.27575335355489	-2.33961929603401	-2.31558743766128
H	0.77360921288388	-3.52530144352825	-4.39972973021519
H	0.56799228278147	0.23132217527399	-6.49460565578420
H	0.40870169195089	-2.26113242429852	-6.52252597954671

I2

H	1.52360766287085	-1.29969153485286	1.38376278800344
C	0.55695221848671	-0.98277069710985	0.99684333411513
C	-1.93829668183785	-0.08525645338409	-0.05647900605668
C	-0.01147476123942	-1.61086525570506	-0.15467668242966
C	-0.09446307021430	0.14600423326108	1.55444136226058
C	-1.36694509785678	0.57855489714384	1.05224583173234
C	-1.25470193074769	-1.17406529952550	-0.68466471078696
H	0.54315916528419	-2.38915699832601	-0.67539506638970
H	0.39417802308593	0.71791335953131	2.34291717971561
H	-1.83687671120062	1.47500859383141	1.45234072473680
H	-1.63844836473711	-1.60545773211767	-1.60670207528490
H	-2.83006504757908	0.32611755050062	-0.52607167802851
Ru	0.10849083014594	0.50492172598966	-0.58425021142797
Cl	-0.90284283339313	1.71520846978019	-2.35087682852424
O	1.05278968604552	2.38819781848751	-0.25764488600863
C	1.71357085498132	2.77090031645202	-1.26295932134456
N	2.32961067541940	1.84080783277164	-2.06898460494658
H	3.64166570921143	1.36640298450746	-0.69269995850329
H	2.67258908509716	2.13958490197904	-2.98188146524040
N	1.93785220591935	4.07584022855576	-1.48441149724187
H	2.43754229265030	4.41488013303382	-2.29778403025734
H	1.40623444510296	4.74105306038171	-0.93360913842872
C	1.90977414099460	0.43460697066381	-1.98740497401909
C	2.83998815037187	-0.24921791668050	-1.10936122137067
O	3.71132512980797	0.42615924522449	-0.38225861591862
O	2.89911361058387	-1.54758586275456	-1.01513701686398
C	3.90672309156158	-2.16935622705936	-0.12033500748810
H	3.78199214666010	-1.73588436638276	0.88052428932214
H	4.89584893201818	-1.89071454650256	-0.50562096463990
C	3.66260927500249	-3.65684070390830	-0.15380220745988
H	4.41399369864631	-4.15325159448225	0.47534009440993
H	3.75742995145968	-4.05202014230127	-1.17312163481697
H	2.67014888899255	-3.91240564709192	0.24057946627950
C	1.53278351260123	-0.17798100943499	-3.30616208268651
C	0.82134433408409	-1.26024464655081	-5.82068008616793
C	1.25605382096275	0.65469078112202	-4.40497081339811
C	1.42318556152268	-1.56848135587410	-3.49235803139891
C	1.07318011059972	-2.09872815666042	-4.73275626285756
C	0.91245108472524	0.11948691822548	-5.64683223887681
H	1.24555037471629	1.73791407624304	-4.29576749649673
H	1.60714014448297	-2.25053518982276	-2.66852642947360
H	0.99657432617175	-3.18057694838788	-4.84688760877244
H	0.69871506863963	0.79377328185413	-6.47627149881030
H	0.54700294689921	-1.67832969262452	-6.78902629315915

Prod

H	-1.76976455263920	-0.61100985665691	-2.84743112056225
C	-2.05477232605200	-0.18753852321690	-1.88353917700679
C	-2.58825857643500	1.15287095219470	0.58941960770885
C	-2.98093281149631	0.90196174112779	-1.82203697763933
C	-1.35875286683584	-0.57216981588872	-0.71745947853031
C	-1.58121626003109	0.13441899591749	0.50480017930001
C	-3.29561588547002	1.53372976089305	-0.57455071013780
H	-3.41408724331041	1.29127486395952	-2.74376042570298
H	-0.53023812821173	-1.27399082899479	-0.78905876164039
H	-0.93364660777114	-0.05785351146630	1.36016206931051
H	-3.95834670810063	2.39745000566801	-0.55264942265063
H	-2.70386729465447	1.72608976317314	1.50831703031116
Ru	-1.16103698035783	1.60458758015142	-0.96961170428623
Cl	-0.79433217004032	3.80886720185684	-0.49648673635354
O	0.58703949405277	1.39332941352176	-1.99996328011076
C	1.75705422082553	1.88918761062735	-2.04671929465942
N	2.75073369955730	1.10395523397774	-2.54153526122965
H	3.68352895157489	1.49939988535482	-2.59675716257696
N	2.02830146266533	3.15827845612632	-1.70702320300570
H	2.97674371728356	3.50496605814628	-1.62187832702205
H	1.25316634554465	3.71194727485610	-1.32571964710888
C	0.85150375470964	-2.06667809063779	-3.17375706113577
C	-0.03762703179356	-0.56377603359192	-5.34658120914982
C	1.47067636205470	-0.84656323000153	-3.46776990449217
C	-0.20953759783401	-2.53080585800973	-3.95692844710278
C	-0.66169261156958	-1.77817318585829	-5.04311053869918
C	1.02374352543485	-0.10416781749008	-4.56712825592862
H	-0.67620324008787	-3.48749911503617	-3.71980892373399
H	-1.48510073247962	-2.14193156851127	-5.65830241901766
H	1.50205667096326	0.84513659716105	-4.81014988534793
H	-0.37403831823446	0.02518745923924	-6.20038713216068
H	1.20463268995334	-2.67816795037412	-2.34237040909387
C	2.65735312123659	-0.35352306613675	-2.64063838900944
H	3.58013499037886	-0.67449755895621	-3.14652988034573
C	2.66976758824754	-0.98409158725814	-1.23150214757389
O	2.05609260089991	-0.54842229712037	-0.27610420969353
O	3.44792417032762	-2.07593377424486	-1.22494129936305
C	3.53076802195457	-2.80816585607077	0.04760577358267
H	2.52112755046646	-3.16642125565968	0.29525595448860
H	3.83952421939260	-2.09573020264041	0.82445348389130
C	4.51737612646399	-3.93648198658809	-0.14051504127210
H	4.60050550486997	-4.50565707580507	0.79553835042952
H	5.51380156809937	-3.55299576087575	-0.39611205891369
H	4.19151158644754	-4.62439304686187	-0.93173454676457

TS$_B$

Ru	-3.35830106690901	-7.90472828811115	-3.02906409652410
Cl	-1.58939729699893	-8.44079539562950	-1.48877769057481
C	-2.86579431577809	-6.17374807583055	-4.36315285370541
H	-2.11609608285275	-6.17673259524943	-5.15281017080955
C	-4.19080101938575	-6.65176902609369	-4.64283999986514
C	-4.76360493887293	-6.43285210749650	-2.25501733533801
H	-5.46044674088084	-6.56888547228003	-1.43032910948420
C	-3.44145772934065	-6.00827853329654	-1.98322439133980
C	-2.49667976574292	-5.83557585532298	-3.05251556766171
H	-1.46462222445705	-5.57969520925640	-2.82235413376783
C	-5.14231537702889	-6.75326479546995	-3.60241820755183
H	-6.14260185430971	-7.12820129817005	-3.81977734955595
C	-4.76854788454136	-10.68373765362460	-3.45280189481338
C	-5.53281299336639	-12.50673146757459	-5.45363179913688
C	-5.20929279306119	-10.23191340663878	-4.71208094983080
C	-4.73542159747666	-12.07046413310694	-3.20728497150955
C	-5.09809350377831	-12.96939810304244	-4.20962682170066
C	-5.59559639324488	-11.13082486529827	-5.69951637561561
H	-5.24101356814876	-9.16228039891897	-4.90883129124208
H	-4.40311862251812	-12.44405747394552	-2.24227073478616
H	-5.05630164014229	-14.04010511946185	-4.00855651858198
H	-5.94580773349522	-10.76117855403912	-6.66371137508803
H	-5.83127547727795	-13.21482233593355	-6.22731868449153
C	-3.86349920312119	-10.21150551474546	-1.07985719174495
O	-3.08244938445246	-11.13806380987066	-1.01229162377512
O	-4.32976164244487	-9.50405458156654	-0.04089386208423
C	-3.73002112258232	-9.82267689953821	1.26359936866481
H	-2.64065492740390	-9.77930561731364	1.13610525009571
H	-4.01479271033874	-10.85156741761059	1.52282331371750
C	-4.24371980348746	-8.80746097444708	2.25643911765690
H	-3.94407333774602	-7.79031307311166	1.97112240794810
H	-3.81641389664127	-9.02219909432638	3.24545056380795
H	-5.33774167471109	-8.84712417797103	2.34391104078753
O	-2.47524781700628	-9.12105823835502	-4.54460927082039
C	-1.89752157049281	-10.25763808985073	-4.54100371457444
N	-1.92333905566546	-10.97438309796738	-5.69597672009246
H	-2.36567923073750	-10.53560285104771	-6.49409965432176
H	-1.23137384103437	-11.69183768942179	-5.87824183908394
N	-1.27505777551920	-10.77479558155603	-3.47717566761238
H	-1.26646479054907	-10.21925619108935	-2.61057868238588
H	-1.03706627959796	-11.75819208145040	-3.43489507330618
H	-3.12062800726179	-5.86302623925274	-0.95376669941794
H	-4.44565477741775	-6.97703526602275	-5.65044332331044
C	-4.39237677469386	-9.68055180068249	-2.40829699278733
N	-5.93980006453735	-9.18192899457409	-1.91176273284460
N	-6.98419269294679	-8.80362155443271	-1.87756169154019

Complex B

Ru	-3.33960050782868	-7.84325189355860	-2.99925039607410
Cl	-1.70774015290331	-8.45602928784088	-1.38315026197904
C	-2.85245891568464	-6.28104823139648	-4.41555506069218
H	-2.11615498310475	-6.38874775746625	-5.21104256496984
C	-4.21101792077623	-6.66417422197825	-4.65292180076488
C	-4.70050401641325	-6.31807429057889	-2.25430736886994
H	-5.38406541043694	-6.41296393996060	-1.41161082124843
C	-3.36540325741914	-5.90891783053919	-2.03221267822018
C	-2.43062747539100	-5.88676027247076	-3.12039769789692
H	-1.37862532243979	-5.68625639615649	-2.92672685877417
C	-5.12274090635689	-6.69079957533804	-3.57447816356875
H	-6.13886204074146	-7.05217656152647	-3.73402730394047
C	-4.89150171258572	-10.73890537394656	-3.47015385643103
C	-5.34029540213711	-12.43190939935392	-5.66733638798181
C	-5.46640621216867	-10.24219064619571	-4.65121711331536
C	-4.54904482076262	-12.09669667020953	-3.39557893617240
C	-4.76839571172188	-12.92997134524797	-4.49593485531441
C	-5.69344268289280	-11.08027915185724	-5.73979544303370
H	-5.72629687039574	-9.18661287420702	-4.72452563827234
H	-4.10646096215445	-12.49517192566259	-2.48720675021708
H	-4.50393784977019	-13.98546999301939	-4.42246921364053
H	-6.14964292886686	-10.67856326999716	-6.64506618039778
H	-5.52214961155752	-13.09246217205235	-6.51549057551325
C	-4.02354365706658	-10.27997324332121	-1.01138900795447
O	-3.23872171758185	-11.20057451029698	-0.97616103649722
O	-4.43008830693747	-9.54199769584575	0.02671361378912
C	-3.76700984371067	-9.82208602346912	1.31087963179902
H	-2.68568123826322	-9.82300142865332	1.12472320553985
H	-4.07425611441476	-10.82706243456008	1.63095594000448
C	-4.19257534565152	-8.74405792822213	2.27816052060390
H	-3.87030890617624	-7.75399542358661	1.92929172294647
H	-3.72393262193124	-8.92976785188131	3.25420162709374
H	-5.28155745569186	-8.73748988780586	2.41950506976126
O	-2.58965842313362	-9.19479148230995	-4.43444008053110
C	-1.94846427617740	-10.29770210706411	-4.43591640297828
N	-1.96021257579902	-11.00919226554238	-5.59181386378225
H	-2.69396650283805	-10.78300938581515	-6.25353554635008
H	-1.57644147598091	-11.94558039801050	-5.62863145741923
N	-1.23601822810108	-10.74083898449700	-3.39617848737416
H	-1.24233690861387	-10.17729561010285	-2.53587913135853
H	-0.83701548831409	-11.67102992176140	-3.38032382416042
H	-3.01483827298554	-5.72345937503245	-1.01869308814597
H	-4.50749964711341	-7.03811251223580	-5.63163942323837
C	-4.69873933479805	-9.80169442681497	-2.29782467274711
N	-5.88483546243006	-9.20065632080419	-1.98961626017314
N	-6.86385352177928	-8.69243670180545	-1.76184912153848

Complex A

Ru	-3.06750847726723	-7.71482587668600	-3.13365541547032
Cl	-1.97341493837029	-8.70588860059187	-1.38966487858356
C	-3.16277470931079	-5.70571069890402	-3.89374889253789
H	-2.33247005171872	-5.17787386214413	-4.36295621776545
C	-4.05510254200262	-6.47270806837222	-4.69234465819844
C	-5.10458209025108	-7.33138444819244	-2.62943365524146
H	-5.76086419731102	-8.05412962099813	-2.14465337569308
C	-4.29573245802343	-6.46944946338661	-1.83415255681453
C	-3.29753630242314	-5.68504186589742	-2.46362787351726
H	-2.56651272283636	-5.14944142598190	-1.85871871830345
C	-4.99847846108981	-7.31673212048957	-4.06089737246530
H	-5.57669099850772	-8.02742055176398	-4.65078453086110
O	-2.21686114555083	-8.90233622827076	-4.59402085334554
C	-1.39737767554106	-9.88754480270054	-4.68943572403227
N	-1.14672897785396	-10.34616381548435	-5.93285621065283
H	-1.62047302947186	-9.90570243176168	-6.71158364273760
H	-0.52410763921422	-11.12327872360332	-6.11650906670901
N	-0.80176977190935	-10.45596547208506	-3.63731266286602
H	-1.00500222232150	-10.07520521715310	-2.70549455250595
H	-0.15799622690596	-11.23220904406195	-3.73649630405939
H	-4.32480278986149	-6.54513144058128	-0.74850452220713
H	-3.89542257225745	-6.54330322088942	-5.76705031543229

N2

N	-5.21101402712164	-9.54061669224862	-2.11746631384995
N	-6.25118597287836	-9.40841330775138	-1.77608368615005

Phenyl-2-diazoacetate

C	-5.31597697803260	-10.99341728137827	-3.22944701707387
C	-6.34734829022806	-12.59195057657332	-5.31171242735672
C	-5.66294083915705	-10.42621907931311	-4.47235780573638
C	-5.49512974669294	-12.37825836886006	-3.04675420452106
C	-6.00666150025060	-13.15998109267424	-4.08255668468659
C	-6.17136969006128	-11.21840341183481	-5.49864338745409
H	-5.53924397538196	-9.35599583850554	-4.64478256232909
H	-5.23423894567574	-12.82645991247988	-2.09156668031678
H	-6.13864928202275	-14.23094180006937	-3.92120720846230
H	-6.43299003765116	-10.75608027785094	-6.45142550734627
H	-6.74603953499545	-13.21086942912715	-6.11604709760773
C	-4.43871858219047	-10.56809478285216	-0.77920579183018
O	-4.57338297272960	-11.69551891903463	-0.33324593598464
O	-3.95079329458016	-9.51999887556481	-0.05531761734901
C	-3.59457976550322	-9.82690858918977	1.32052566951257
H	-2.84981676530038	-10.63592255740791	1.31732100868732
H	-4.49033069561250	-10.19777093659954	1.83974631984752
C	-3.05761333953948	-8.55418630447121	1.93965001395310
H	-2.16896150075016	-8.19858139961419	1.40082625834020
H	-2.77461612802719	-8.74384670452133	2.98454908643100
H	-3.81482900598131	-7.75857895943607	1.92634786137330
C	-4.77360780120091	-10.15061878947617	-2.14983912885310
N	-4.56098671454855	-8.87660157672274	-2.41700562524103
N	-4.38589561388638	-7.78058753644261	-2.67386453599616

References

1 de Koning, P. D.; Gladwell, I. R.; Morrison, N. A.; Moses, I. B.; Panesar, M. S.; Pettman, A. J.; Thomson, N. M.; Yazbeck, D. R. Enzymatic Desymmetrization Route to Ethyl [3-(2-Amino-2-Methylpropyl)Phenyl]Acetate. *Org. Process Res. Dev.*, **2011**, *15*, 871–875.

2 Shimomoto, H.; Mukai, H.; Bekku, H.; Itoh, T.; Ihara, E. Ru-Catalyzed Polycondensation of Dialkyl 1,4-Phenylenebis(Diazoacetate) with Dianiline: Synthesis of Well-Defined Aromatic Polyamines Bearing an Alkoxycarbonyl Group at the Adjacent Carbon of Each Nitrogen in the Main Chain Framework. *Macromolecules* **2017**, *50* (23), 9233–9238.

3 Davies, H. M. L.; Grazini, M. V. A. Aouad, E. Asymmetric Intramolecular C−H Insertions of Aryldiazoacetates. *Org*. *Lett*. **2001**, *3*, 1475.

4 Bruker, SAINT V8.40B, Bruker AXS Inc., Madison, Wisconsin, USA, 2001.

5 SADABS-2016/2 - Bruker AXS area detector scaling and absorption correction: Krause, L., Herbst-Irmer, R., Sheldrick G.M. & Stalke D., J. *Appl. Cryst.* **2015**, *48*, 3–10.

6 Sheldrick, G.M. SHELXT—Integrated space-group and crystal-structure determination. *Acta Crystallogr. A* **2015**, *A71*, 3–8.

7 Sheldrick, G. M. Crystal structure refinement with SHELXL. *Acta Crystallogr. C Struct. Chem.* **2015**, *C71*, 3–8.

8 Spek, A. L. (2001). PLATON. Utrecht University, The Netherlands. (http://www.Cryst.Chem.uu.nl)

9 Slot, T. K.; Shiju, N. R.; Rothenberg, G. A Simple and Efficient Device and Method for Measuring the Kinetics of Gas-Producing Reactions. *Angewandte Chemie - International Edition* **2019**, *58* (48), 17273–17276.

10 Slot, T. K.; Riley, N.; Shiju, N. R.; Medlin, J. W.; Rothenberg, G. An Experimental Approach for Controlling Confinement Effects at Catalyst Interfaces. *Chemical Science* **2020**, *11* (40), 11024–11029.

11 Haque, F.M., Grayson, S.M. The synthesis, properties and potential applications of cyclic polymers. *Nat. Chem.* **2020**, *12*, 433–444.

12 Hashimoto, T.; Yamamoto, K.; Maruoka, K. Development of a Practical Synthetic Method for *N-Tert*-Butoxycarbonyl α-Ketimino Esters. *Chem. Let.* **2011**, *40*, 326–327.

13 O'Connor, N. R.; Bolgar, P.; Stoltz, B. M. Development of a Simple System for the Oxidation of Electron-Rich Diazo Compounds to Ketones. *Tetrahedron Lett.* **2016**, *57*, 849–851.

14 Éll, A. H.; Samec, J. S. M.; Brasse, C.; Bäckvall, J.-E. Dehydrogenation of Aromatic Amines to Imines via Ruthenium-Catalyzed Hydrogen Transfer. *Chem. Commun.* **2002**, 1144–1145.

15 Neese, F. The ORCA program system. *Wiley Interdisciplinary Reviews: Computational Molecular Science*. **2012** *2* (1): 73–78.

16 (a) Becke, A. D. Density-functional exchange-energy approximation with correct asymptotic behavior. *Phys. Rev. A* **1988**, *38*, 3098–3110. (b) Perdew, J. P. Density-functional approximation for the correlation energy of the inhomogeneous electron gas. *Phys. Rev. B* **1986**, *33*, 8822–8824. (c) Perdew, J. P. Erratum: Density-functional approximation for the correlation energy of the inhomogeneous electron gas. *Phys. Rev. B* **1986**, *34*, 7406–7406.

17 (a) Weigend, F.; Ahlrichs, R. Balanced basis sets of split valence, triple zeta valence and quadruple zeta valence quality for H to Rn: Design and assessment of accuracy. *Phys. Chem. Chem. Phys.* **2005**, *7*, 3297−3305. (b) Weigend, F.; Haser, M.; Patzelt, H.; Ahlrichs, R. RI-MP2: optimized auxiliary basis sets and demonstration of efficiency. *Chem. Phys. Lett.* **1998**, *294*, 143−152.

18 Grimme, S.; Antony, J.; Ehrlich, S.; Krieg, H. A consistent and accurate ab initio parametrization of density functional dispersion correction (DFT-D) for the 94 elements H-Pu. *J. Chem. Phys.* **2010**, *132*, 154104−154119.
